# Resuscitation with blood products in patients with trauma-related haemorrhagic shock receiving prehospital care (RePHILL): a multicentre, open-label, randomised, controlled, phase 3 trial

**DOI:** 10.1016/S2352-3026(22)00040-0

**Published:** 2022-03-07

**Authors:** Nicholas Crombie, Heidi A Doughty, Jonathan R B Bishop, Amisha Desai, Emily F Dixon, James M Hancox, Mike J Herbert, Caroline Leech, Simon J Lewis, Mark R Nash, David N Naumann, Gemma Slinn, Hazel Smith, Iain M Smith, Rebekah K Wale, Alastair Wilson, Natalie Ives, Gavin D Perkins, RePHILL collaborative group

**Affiliations:** a NIHR Surgical Reconstruction and Microbiology Research Centre, Queen Elizabeth Hospital, University Hospitals Birmingham NHS Foundation Trust, Birmingham, UK; b NHS Blood and Transplant, Birmingham, UK; c Birmingham Clinical Trials Unit, Institute of Applied Health Research, University of Birmingham, Birmingham, UK; d Pharmacy Department, Queen Elizabeth Hospital, Birmingham, UK; e West Midlands Ambulance Service NHS Trust, West Midlands, UK; f Blood transfusion, The Royal Wolverhampton NHS Trust, Wolverhampton, UK; g The Air Ambulance Service, Blue Skies House, Butlers Leap, Rugby, UK; h Magpas Air Ambulance, Huntingdon, UK; i Midlands Air Ambulance and MERIT, West Midlands Ambulance Service NHS Trust, West Midlands, UK; j Institute of Inflammation and Aging, University of Birmingham, Birmingham, UK; k East Anglian Air Ambulance, Norwich, UK; l Warwick Clinical Trials Unit, Warwick Medical School, University of Warwick, Coventry, UK; m Critical Care Unit, Heartlands Hospital Birmingham, University Hospitals Birmingham NHS Foundation Trust, Birmingham

## Abstract

**Background:**

Time to treatment matters in traumatic haemorrhage but the optimal prehospital use of blood in major trauma remains uncertain. We investigated whether use of packed red blood cells (PRBC) and lyophilised plasma (LyoPlas) was superior to use of 0·9% sodium chloride for improving tissue perfusion and reducing mortality in trauma-related haemorrhagic shock.

**Methods:**

Resuscitation with pre-hospital blood products (RePHILL) is a multicentre, allocation concealed, open-label, parallel group, randomised, controlled, phase 3 trial done in four civilian prehospital critical care services in the UK. Adults (age ≥16 years) with trauma-related haemorrhagic shock and hypotension (defined as systolic blood pressure <90 mm Hg or absence of palpable radial pulse) were assessed for eligibility by prehospital critial care teams. Eligible participants were randomly assigned to receive either up to two units each of PRBC and LyoPlas or up to 1 L of 0·9% sodium chloride administered through the intravenous or intraosseous route. Sealed treatment packs which were identical in external appearance, containing PRBC–LyoPlas or 0·9% sodium chloride were prepared by blood banks and issued to participating sites according to a randomisation schedule prepared by the co-ordinating centre (1:1 ratio, stratified by site). The primary outcome was a composite of episode mortality or impaired lactate clearance, or both, measured in the intention-to-treat population. This study is completed and registered with ISRCTN.com, ISRCTN62326938.

**Findings:**

From Nov 29, 2016 to Jan 2, 2021, prehospital critical care teams randomly assigned 432 participants to PRBC–LyoPlas (n=209) or to 0·9% sodium chloride (n=223). Trial recruitment was stopped before it achieved the intended sample size of 490 participants due to disruption caused by the COVID-19 pandemic. The median follow-up was 9 days (IQR 1 to 34) for participants in the PRBC–LyoPlas group and 7 days (0 to 31) for people in the 0·9% sodium chloride group. Participants were mostly white (62%) and male (82%), had a median age of 38 years (IQR 26 to 58), and were mostly involved in a road traffic collision (62%) with severe injuries (median injury severity score 36, IQR 25 to 50). Before randomisation, participants had received on average 430 mL crystalloid fluids and tranexamic acid (90%). The composite primary outcome occurred in 128 (64%) of 199 participants randomly assigned to PRBC–LyoPlas and 136 (65%) of 210 randomly assigned to 0·9% sodium chloride (adjusted risk difference –0·025% [95% CI –9·0 to 9·0], p=0·996). The rates of transfusion-related complications in the first 24 h after ED arrival were similar across treatment groups (PRBC–LyoPlas 11 [7%] of 148 compared with 0·9% sodium chloride nine [7%] of 137, adjusted relative risk 1·05 [95% CI 0·46–2·42]). Serious adverse events included acute respiratory distress syndrome in nine (6%) of 142 patients in the PRBC–LyoPlas group and three (2%) of 130 in 0·9% sodium chloride group, and two other unexpected serious adverse events, one in the PRBC-LyoPlas (cerebral infarct) and one in the 0·9% sodium chloride group (abnormal liver function test). There were no treatment-related deaths.

**Interpretation:**

The trial did not show that prehospital PRBC–LyoPlas resuscitation was superior to 0·9% sodium chloride for adult patients with trauma related haemorrhagic shock. Further research is required to identify the characteristics of patients who might benefit from prehospital transfusion and to identify the optimal outcomes for transfusion trials in major trauma. The decision to commit to routine prehospital transfusion will require careful consideration by all stakeholders.

**Funding:**

National Institute for Health Research Efficacy and Mechanism Evaluation.

**Research in context**

**Evidence before this study**

We conducted a systematic review of studies which compared prehospital blood components (red blood cells, plasma, or whole blood), in both civilian and military settings, with other resuscitative fluids in patients with major traumatic haemorrhage (PROSPERO CRD42014013794). We searched MEDLINE, MEDLINE In Process, EMBASE, The Cochrane Library, UK Blood Services Transfusion Evidence Library, Defence Medical Library Service, Science Citation Index, British Library's ZETOC and ISI Proceedings and trial registries from database inception to July, 2015. A combination of alternative text and MeSH terms relating to the condition (haemorrhage), intervention (blood components) and setting (pre-hospital) were used to search the databases. No language restrictions were applied. Studies were assessed for risk of bias and the quality of evidence was assessed using GRADE methodology. The search identified low quality evidence from 27 observational studies (16 case series and 11 comparative studies). No definitive evidence of benefit was found for prehospital blood components and survival (odds ratio for mortality 1·29 [95% CI 0·84–1·96]), physiology, or in-hospital transfusion requirements. Transfusion reactions were rare, suggesting the short-term safety of prehospital blood components administration.

**Added value of this study**

This prospective multi-centre randomised controlled superiority trial did not show that prehospital packed red blood cells (PRBC) and lyophilised plasma (LyoPlas) resuscitation was superior to 0·9% sodium chloride for trauma related haemorrhagic shock in the civilian population studied. Although the point estimates for the individual components of the primary outcome and some of the secondary outcomes are in the direction of a potential benefit from allocation to PRBC–LyoPlas, the confidence intervals indicate the possibility of both benefit and harm. Subgroup analyses did not find evidence that the treatment effect was different in those with longer transport times.

**Implications of all the available evidence**

The decision to commit to routine prehospital blood transfusion in civilian practice will require careful consideration by all stakeholders. Future research should seek to identify if specific patient cohorts benefit and explore the effects of alternative transfusion strategies.

## Introduction

During the past two decades, changes in trauma resuscitation practice have seen substantial changes, much of which has been influenced by the lessons learnt during conflict. Treatment of haemorrhagic shock is now increasingly focused on the early use of haemorrhage control, tranexamic acid,^1^ and blood-based resuscitation with packed red cells and plasma.^2^, ^3^ The improved survival from these and other strategies within streamlined trauma systems have been reported in the military^4^ and civilian settings,^5^, ^6^ and have stimulated an interest in prehospital transfusion.

The early use of blood components during in-hospital trauma resuscitation of patients at risk of massive haemorrhage and shock is growing.^7^, ^8^ The practice is increasingly being transferred to civilian prehospital practice following some early reports of benefit during military casualty retrieval using red blood cells and prethawed plasma within an advanced medical retrieval capability.^9^, ^10^ O'Reilly and colleagues^11^ suggested a halving of mortality among recipients in a UK matched cohort military study after having corrected for potential confounders. However, a 2016 meta-analysis of outcomes following prehospital transfusion for both military and civilian patients with trauma suggested that any survival advantages were modest and limited to moderate injury severity.^12^ Subsequently, a US retrospective military analysis suggested survival advantage at both 24 h and 30 days with use of prehospital blood products.^13^ The only two large randomised trials to assess prehospital transfusion strategies randomly assigned participants to plasma in addition to standard care (0·9% sodium chloride). The trials produced discordant results: one favoured prehospital plasma^14^ and the other did not.^15^ A 2020 review of prehospital red blood cell transfusion highlighted the absence of randomised trials and was unable to demonstrate a survival benefit, but recommended further studies with plasma and individualised transfusion criteria.^16^

Extending the use of both packed red blood cells (PRBC) and plasma for major haemorrhage into the prehospital environment might seem intuitive but has implications for the transfusion and clinical communities. Transfusion specific concerns include sustaining the demand for universal blood products, blood product wastage, and regulatory compliance. Clinical concerns include speed and safety of administration and identifying which patients might benefit. Experts in the field have called for “prospective studies…to clarify the role of transfusion based prehospital haemorrhagic shock resuscitation in civilian practice” and have stressed the importance of prospective data collection.^17^, ^18^ Evidence is required to justify the logistical, training, and financial burdens of bringing a traditionally hospital-based treatment into the prehospital domain. We aimed to investigate the hypothesis that the use of prehospital PRBC and lyophilised plasma (LyoPlas) would improve tissue perfusion, as measured by lactate clearance, or reduce mortality in trauma participants with haemorrhagic shock compared with resuscitation with crystalloids.

## Methods

### Study design and participants

Resuscitation with Pre-Hospital Blood Products (RePHILL) was a multicentre, phase 3, allocation concealed, open-label, parallel group, randomised controlled trial. The study was based across four prehospital critical care services and their associated trauma networks in the UK National Health Service (appendix p 111). Prehospital critical care teams, typically comprising of a physician and critical care paramedic, were assigned to cases of suspected major trauma, travelling by helicopter or land-based rapid response vehicle. The study was approved by the South Central Research Ethics Committee (15/SC/0691) and the Medicines and Healthcare products Regulatory Agency. The study protocol has been published and is also available in the appendix (p 1).^19^

Participants were assessed for eligibility by prehospital medical teams. Adults (age ≥16 years) with traumatic injury and with hypotension (defined as systolic blood pressure <90 mm Hg or absence of palpable radial pulse) believed to be due to a traumatic haemorrhage were eligible for inclusion. The exclusion criteria were transfusion of prehospital blood products before assessment for eligibility, known refusal of prehospital blood products, pregnancy (known or apparent), isolated head injury without evidence of major haemorrhage, and prisoners (for regulatory reasons as we did not apply to the prisons service ethical review board). On Jan 25, 2017, the protocol was amended to allow treatment to be administered through the intraosseous route and to exclude participants with traumatic cardiac arrest where the cardiac arrest occurred before arrival of the prehospital emergency medicine team or the primary cause is not hypovolaemia.

Due to the nature of their injury, and urgent need to provide treatment, patients with traumatic haemorrhagic shock were unable to provide informed consent to participate in a clinical trial and so consent by participants was deferred until the emergency had passed, in accordance with the Medicines for Human Use (Clinical Trials) Regulations. We sought written informed consent to continue data collection after arrival in hospital from either the participant or a personal or professional representative. Participants who were later found to be ineligible, but who have received the trial intervention, remained in the trial as per protocol and were included in the analysis.

### Randomisation and masking

The randomisation process involved blood bank staff placing either PRBC–LyoPlas or 0·9% sodium chloride into sealed treatment boxes according to a randomisation schedule (1:1 ratio, block randomisation [variable block size], stratified by site) implemented through a central and secure trial database at the Birmingham Clinical Trials Unit. Boxes were otherwise externally identical in appearance and weight, thus ensuring allocation concealment. Boxes were issued to the relevant prehospital team and carried on the emergency vehicles for up to 48 h before being replaced if unused. Boxes had continuous temperature monitoring and returned blood products were placed back into the blood bank stock. When a participant met the trial eligibility criteria, they were enrolled by opening the sealed boxes, and at this point, participants were considered randomly assigned into the study. The prehospital teams were unaware of the treatment allocation before enrolling a participant. Once randomly assigned, health-care professionals administering the trial intervention were aware of group assignment. Clinicians assessing the outcomes were not informed of group assignments but might have been able to access them through hospital records. An independent data monitoring committee met on five occasions during the trial and were masked to the allocated treatment. The trial did not formally evaluate the success of masking.

### Procedures

Participants allocated to the intervention group received up to four units of blood products in one-unit boluses (up to two units of PRBC and up to two units of LyoPlas). Blood group O, Rhesus factor D negative, and Kell negative leucodepleted red cells in additive solution (SAG-M) were provided by the UK National Health Service Blood and Transplant. The mean volume of one unit of packed red blood cells was 282 mL (range 220–340). Lyophilised plasma (LyoPlas N-w [LyoPlas]; blood groups A or AB; DRK-Blutspendedienst West, Ratingen, Germany) was reconstituted in 200 mL water (total volume 213 mL) for injection immediately before administration. The protocol recommended alternating one unit of PRBC with one unit of LyoPlas in accordance with UK blood transfusion guidelines.^20^ Individuals allocated to the control group received up to four (250 mL) bags of 0·9% sodium chloride. Consequently, over the four boluses, similar volumes of fluid should have been administered in each trial group. Both the intervention and control fluids were delivered through a fluid warmer.

For both groups, the interventions were administered until either hospital arrival, a return of a systolic blood pressure to 90 mm Hg or more, or a radial pulse was palpable. If the blood pressure decreased on the way to the hospital, treatment was re-instigated. If all four units of intervention were given, non-trial sodium chloride was then given as per standard UK ambulance service practice. Following arrival to the hospital, further resuscitation and transfusion was at the discretion of the treating physicians.

A measure of capillary blood lactate was taken (Lactate Scout/Solo, HaB International, Southam, England, UK) before delivering the allocated intervention, and then a second lactate measurement was taken 2 h after randomisation. Participants were followed up until discharge from acute care, withdrawal from the trial, or death, whichever occurred first. Trial assessments took place at the scene, on arrival at the emergency department, at 2 h post randomisation, after emergency department arrival at hours 2, 6, 12, and 24, and during the participants hospital stay (through to day 30). Adverse events were collected and reported in accordance with the Medicines for Human Use (Clinical Trials) Regulations 2004 and subsequent amendments. Any other adverse or serious adverse events were recorded until the end of follow-up for each participant. The Principal Investigators assessed the seriousness and causality of all applicable adverse events. Serious adverse events of organ failure, multi-organ failure, acute respiratory distress syndrome, infection, venous thromboembolism, and transfusion reactions were captured on the case report forms. Any other serious adverse events were reported directly to the trial office. For participants who withdrew consent for continuing in the trial, data already collected up until the point of withdrawal were retained and included in the analysis. Data after the point of withdrawal were not collected. Participants were withdrawn from the study if ineligible and did not receive trial interventions, did not provide or withdrew consent, or were lost to follow-up.

### Outcomes

The primary outcome was reported by participating centres and comprised of a composite of episode mortality (death at any time between injury and discharge from the primary receiving facility to non-acute care) or a failure to reach lactate clearance (<20% per h in the first 2 h after randomisation), or both. The secondary outcomes were the individual components of the primary outcomes, all-cause mortality within 3 h and 30 days of randomisation, prehospital timings, type and volume of fluid administered before and after the intervention, vital signs, venous lactate concentration, haemoglobin concentration and trauma-induced coagulopathy, total blood product receipt, organ-failure free days, the incidence of acute respiratory distress syndrome, and transfusion related complications. Coagulation and platelet function tests will be reported separately.

### Statistical analysis

Few existing data for the composite primary outcome were available at the start of the trial. Based on extensive discussions between the investigators and a panel of international experts and informed by a systematic review of prehospital blood product transfusion,^12^ we anticipated that a 10% absolute difference in the primary outcome (20% control and 10% intervention) would be considered clinically meaningful. To detect a 10% difference between groups in the proportion of participants who met the primary outcome, with 80% power and type 1 error rate of 0·05, we required 438 participants (219 per group). Allowing for 10% attrition, this number increased to 490 participants. At a data monitoring committee meeting on May 3, 2018, the pooled event rate (65%) and the sample size assumptions were discussed, and they recommended that these were discussed with the Trial Steering Committee. Subsequently, it was agreed that the power calculations be framed in terms of a relative risk rather than an absolute risk, with the original sample size unchanged (protocol amendment 3.0, April 8, 2019; appendix p 47). This participant number gave 80% power to detect a relative risk of 0·82 (71·7% control and 58·3% intervention).

The data monitoring committee met before the trial opening and again once the first 25 patients had been entered into the study. The committee then met on four further occasions (annually) during the trial to review interim data and specifically to monitor patient safety and trial conduct. Before the study began, the committee members agreed that a difference of at least p<0·001 (similar to a Haybittle-Peto stopping boundary) in an interim analysis of a major endpoint was required to justify halting, or modifying, the study prematurely. By adopting this criterion, the exact number of interim analyses did not need to adhere to a prespecified fixed schedule. The committee reviewed data masked to treatment allocation.

All primary analyses of the primary and secondary outcomes followed the intention-to-treat principle (ie, analysis according to the randomisation schedule irrespective of treatment received). Participants withdrawn from the study were not assessable. The analyses used a model-based approach with prehospital critical care service included as a fixed-effect covariate in the model. Treatment effects are presented with two-sided 95% CIs. No adjustment for multiple comparisons was made. All analyses were undertaken in SAS (version 9.4). The statistical analysis plan is available in the appendix (p 59).

Both the relative effect and absolute effect were reported for binary outcomes (eg, primary outcome and the individual components). Binary outcomes were analysed using log-binomial regression models to obtain adjusted relative risks along with 95% CI. A relative risk of less than 1 favoured the PRBC–LyoPlas group. Adjusted risk differences along with 95% CI were estimated using a binomial regression model with identity link. A risk difference of less than 0 favoured the PRBC–LyoPlas group. Continuous data were analysed using linear regression models to obtain adjusted mean differences between groups along with 95% CI.

We planned, a priori*,* a Bayesian analysis of the primary outcome and its individual components using non-informative, sceptical, and informative priors. We also planned, a priori, various exploratory subgroup analyses according to: intervention delivery site, mode of transport (air *vs* ground), initial lactate concentration (≤2·2 mmol/L *vs* >2·2 mmol/L), time to hospital arrival from injury (≤1 h *vs* >1 h), mode of injury (blunt *vs* penetrating *vs* crush), volume of prehospital fluid given (total intervention of four boluses *vs* less than four boluses), age (<50 years, 50–70 years, >70 years), presence of head injury, compressible haemorrhage, previous history of anticoagulant or antiplatelet use (anticoagulant or antiplatelet medication *vs* no anticoagulant or antiplatelet medication), and cardiac arrest (arrested *vs* not arrested). The following sensitivity analyses were also done for the primary outcome: model adjusting for intervention delivery site, cardiac arrest, age, capillary lactate concentration, and Glasgow Coma Score at randomisation; differences in lactate timings; per-protocol analysis (the per-protocol population comprised those who received one or more dose of the randomised intervention or control, unless there was a clinical justification for withholding it); and influence of missing data. We used post-hoc analyses to compare total (prehospital and hospital) transfusion volume administered between groups, two additional subgroup analyses according to injury severity score and transport time from scene to hospital, and an additional analysis in the per-protocol population. We also modelled different scenarios had the trial achieved the intended sample size.

This study is registered with ISRCTN.com, ISRCTN62326938.

### Role of the funding source

The funder of the study had no role in study design, data collection, data analysis, data interpretation, or writing of the report.

## Results

**Figure 1:**
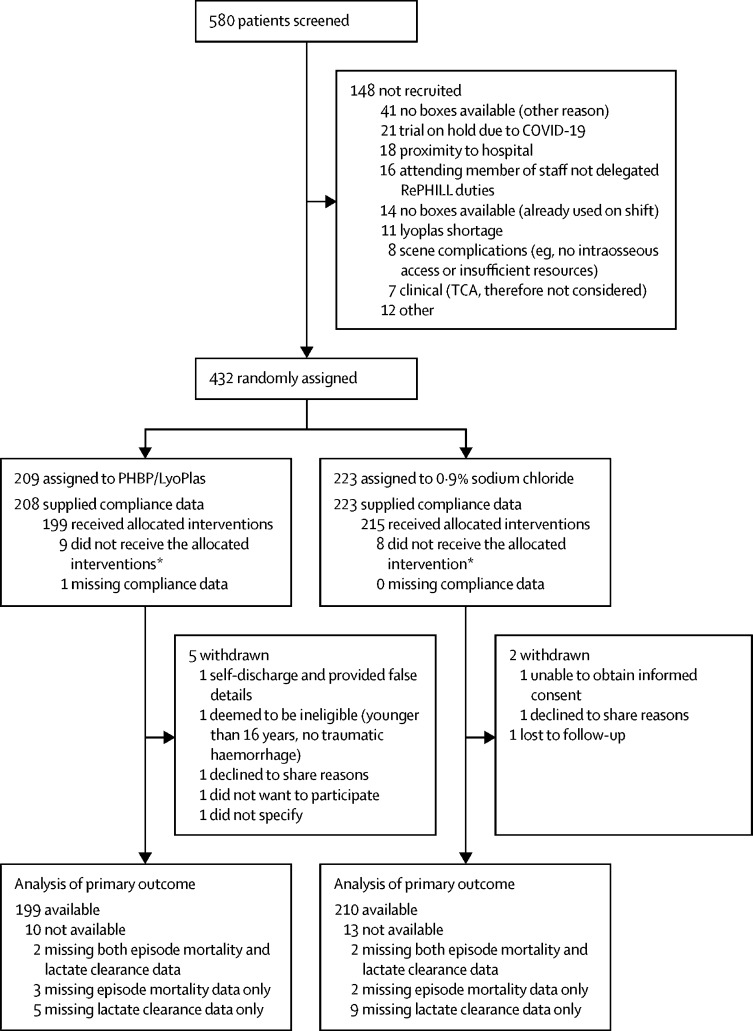
Trial profile Patients eligible were based on screening lists provided by each Intervention Delivery Site. *Reasons for participants not receiving any units of allocated intervention (with no clinical justification) were: nine due to equipment absence or failure (eg, of giving sets or lactate monitors), one due to complex scene conditions, one due to decision to stop resuscitation, one due to non-trial saline already being administered to patient, and five gave no reason.

From Nov 29, 2016 to Jan 2, 2021, prehospital medical teams assessed 580 participants for eligibility. The first participant was recruited on Dec 6, 2016, the final participant was recruited on Jan 1, 2021. Recruitment to the trial was stopped before the intended sample size was achieved due to the effect of the COVID-19 pandemic and the end of the funded recruitment period. This decision to stop the trial was approved by the independent Trial Steering Committee and sponsor, without any knowledge of the data or results of interim analyses. When the trial closed to recruitment, 432 participants had been randomly assigned to the PRBC–LyoPlas group (n=209) or to the 0·9% sodium chloride control group (n=223; figure 1). The median follow-up for all 432 participants was 8 days (IQR 0–34). Split by treatment group, the median follow-up was 9 days (IQR 1–34) for participants in the PRBC–LyoPlas group and 7 days (0–31) for the control group.

Most participants were White (62%) and male (82%), and the median age was 38 years (IQR 26–58; table 1). The most common causes for major trauma were from road traffic collision, stabbing, and falling. 61 (48%) of 128 had concurrent brain injury. The pattern of injury was mostly blunt and some penetrating trauma. Before randomisation, participants had received an average of 430 mL (SD 490) crystalloid fluids and tranexamic acid (90%). The average blood pressure was 73/46 mm Hg. The median injury severity score was 36 (IQR 25–50) and median new injury severity score was 43 (34–57). Most participants were transported to hospital by road ambulance (62%), arriving on a median of 83 min [IQR 65–101] after emergency call. Participants in the PRBC–LyoPlas group received on average 1·57 units (443 mL) of PRBC and 1·25 units (266 mL) of LyoPlas, while people in the 0·9% sodium chloride group received on average 2·55 units (638 mL) of 0·9% sodium chloride. In practice, whole units were administered as boluses.

**Table 1:** Baseline characteristics

		**Packed red blood cells and lyophilised plasma group (n=209)**	**0·9% sodium chloride group (n=223)**
**Stratification variable**			
Intervention delivery site			
	Site 1	68 (32%)	64 (29%)
	Site 2	37 (18%)	41 (18%)
	Site 3	60 (29%)	61 (27%)
	Site 4	44 (21%)	57 (26%)
**Demographic and other baseline variables**			
Sex			
	Male	170/208 (82%)	183 (82%)
	Female	38/208 (18%)	40 (18%)
Age, years		38 (27–57); n=196	39 (24–59); n=211
Ethnic group*			
	White	104/166 (63%)	104/168 (62%)
	Black	2/166 (1%)	3/168 (2%)
	Mixed	4/166 (2%)	5/168 (3%)
	Asian	8/166 (5%)	8/168 (5%)
	Other	1/166 (1%)	4/168 (2%)
	Not known or provided	47/166 (28%)	44/168 (26%)
**Injury details**			
Injury mechanism†			
	Road traffic collision	130 (62%)	139 (62%)
	Stabbing	33 (16%)	35 (16%)
	Fall	26 (12%)	35 (16%)
	Gunshot	4 (2%)	4 (2%)
	Burn	0	1 (<1%)
	Inhalation	1 (1%)	0
	Other‡	19 (9%)	22 (10%)
Injury characteristics			
	Concomitant head injury§	29/60 (48%)	32/68 (47%)
	Compressible haemorrhage	50/208 (24%)	49 (22%)
	Non-compressible haemorrhage	171/208 (82%)	186 (83%)
	Traumatic cardiac arrest¶	21/151 (14%)	20/175 (11%)
	Blunt force trauma	162/208 (78%)	178 (80%)
	Penetrating trauma	47/208 (23%)	48 (22%)
	Crush trauma	6/208 (3%)	2 (1%)
**Prehospital timeline**			
Time from call to emergency services to arrival on scene, min		30 (23); n=209	31 (18); n=223
Time from arrival on-scene to administration of first intervention, min		26 (16); n=201	25 (17); n=209
**On-scene vital signs**			
Heart rate, bpm‖		115 (31); n=185	109 (33); n=198
SBP, mmHg‖		73 (16); n=128	73 (20); n=148
DBP, mmHg‖		47 (13); n=125	46 (16); n=147
Respiratory rate per min‖		24·3 (9·5); n=172	23·4 (10·6); n=186
Oxygen saturation, %‖		92 (8); n=131	91 (9); n=144
Glasgow Coma Scale		8 (3–14); n=209	6 (3–14); n=222
Capillary lactate concentration, mmol/L		9·13 (4·39); n=199	9·17 (4·98); n=207
Medical history**			
ISS††		36 (25–49); n=148	36 (25–50); n=152
NISS††		43 (34–57); n=144	48 (34–57); n=148
Concomitant treatments			
	Tranexamic acid	182 (87%)	206 (92%)
	Fluid volume given before intervention, mL	422 (499)	437 (482)
Mode of transport			
	Air	80 (38%)	86 (39%)
	Ground	129 (62%)	137 (61%)

Data are mean (SD), median (IQR), or n (%), unless otherwise specified. SBP=systolic blood pressure. DBP=diastolic blood pressure. ISS=injury severity score. NISS=new injury severity score. TARN=Trauma Audit and Research Network.

* Data only available for participants providing an emergency department arrival form.

† Multiple responses are possible.

‡ Other injuries comprise: 13 laceration injuries, six pedestrian incidents with trains, four agricultural incidents, four industrial accidents, and five other injuries.

§ Added in version 4.0 of prehospital case report form (sent to all sites by Aug 29, 2019).

¶ Defined as those with a heart rate of 0 and blood pressure of 0.

‖ Blood pressure, heart rate, respiratory rate, and oxygen saturation are summarised as continuous variables only for participants with non-zero on scene measurements.

** Data only available for the 342 participants providing a medical history form.

†† ISS and NISS will only be available for those participants who are TARN eligible, hence this is not strictly a baseline characteristic, and the number of missing participants refers to the number of TARN eligible participants missing their ISS or NISS.

**Figure 2:**
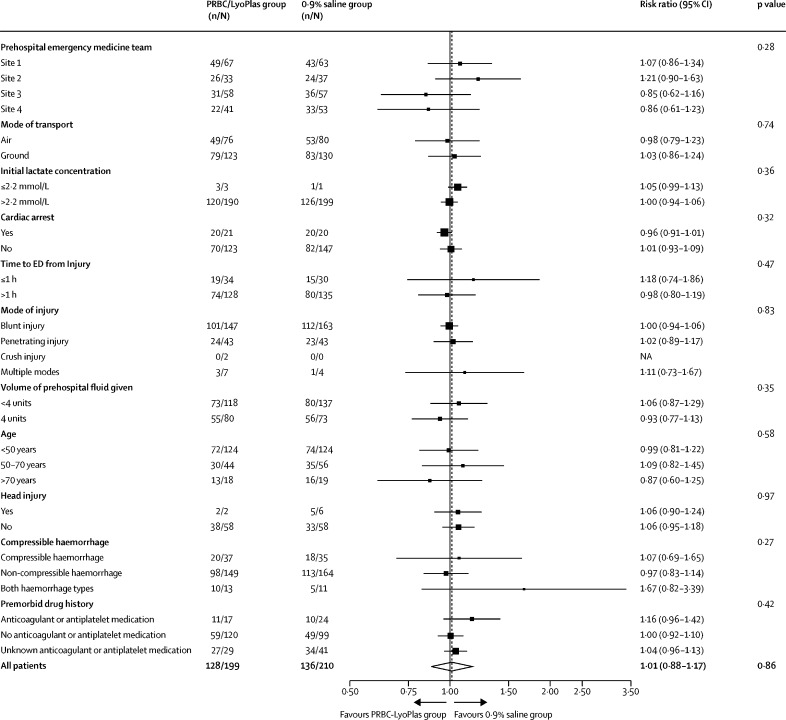
Subgroup analyses for the primary outcome p value for all patients corresponds to p value for the treatment effect. All other p values are for the treatment by subgroup interaction term. Post-hoc subgroup analyses according to injury severity, and transport time are reported in the appendix (p 110). ED=emergency department. NA=not available. PRBC=packed red blood cells.

The primary outcome occurred in 128 (64%) of 199 people in the PRBC–LyoPlas and in 136 (65%) of 210 people in the 0·9% sodium chloride group (adjusted risk ratio 1·01 [95% CI 0·88–1·17], adjusted risk difference –0·025% [–9 to 9]). For the PRBC–LyoPlas group versus control, this meant that 40 individuals (20%) versus 37 (18%) did not clear lactate and survived, 58 (29%) versus 76 (36%) did not clear lactate and died, or 30 (15%) versus 23 (11%) people who cleared lactate died or died without providing a lactate result. The event rates for the individual components of the primary outcome (episode mortality and lactate clearance) were not statistically different between groups (table 2). The point estimate for the number needed to benefit to reduce episode mortality was 36. The 95% CI ranged from a number needed to harm of 15 through to number needed to benefit of eight. The Bayesian analysis found that the probability that the risk difference of more than 0% and more than 10% for the primary outcome was 48·2% and 1·3% for non-informative priors, 44·1% and 0·3% for sceptical priors, and 53·4% and 1·6% for informative priors. Further information on the Bayesian analysis is available in the appendix (p 102). There were no changes to the study findings across the sensitivity analyses for the primary outcome including the per-protocol analyses (appendix p 109). The treatment effect for the primary outcome was consistent across all of the predefined subgroups (figure 2) and the two post-hoc subgroup analyses based on injury severity score and scene to hospital transport time of more or less than 20 min (appendix p 110). Post-hoc simulations examined the effect of deterministically scaling up sample sizes assuming the study would continue to have the same outcome rates in each group. Even after extensive further recruitment, we would still expect to observe a null result for the primary outcome (a projected sample size of 5000 produced an estimated unadjusted risk ratio of 0·99 [95% CI 0·95–1·03]).

**Table 2:** Primary and key secondary outcomes

	**Packed red blood cells and lyophilised plasma group**	**0·9% sodium chloride group**	**Adjusted risk ratio (95% CI)**	**Adjusted average difference (95% CI)**
**Primary outcome**				
Episode mortality or failure to clear lactate, or both	128/199 (64%)	136/210 (65%)	1·01 (0·88 to 1·17)*; p=0·86	−0·025% (−9 to 9)†; p=1·00
**Secondary outcomes**				
Episode mortality	88/203 (43%)	99/218 (45%)	0·97 (0·78 to 1·20)*; p=0·75	−3% (−12 to 7)†; p=0·57
Failure to clear lactate	98/196 (50%)	113/206 (55%)	0·94 (0·78 to 1·13)*; p=0·52	−5% (−14 to 5)†; p=0·33
Post-intervention fluids, mL	123 (310), 207	160 (389), 221	..	−34 (−101 to 32)‡; p=0·31
**Time to ED arrival, mins**				
From 999 call	90 (35)	91 (35)	..	0·60 (−6·14 to 7·35)‡; p=0·86
From randomisation	37 (22)	35 (22)	..	3·03 (−1·40 to 7·46)‡; p=0·18
**Vital signs at ED arrival**				
Heart rate, bpm	107 (29)	105 (24)	..	−0·80 (−5·83 to 4·23)§; p=0·76
Systolic blood pressure, mm Hg	114 (27)	114 (29)	..	−1·19 (−8·19 to 5·82)§; p=0·74
Diastolic blood pressure, mm Hg	75 (24)	72 (24)	..	2·26 (−3·77 to 8·29)§; p=0·46
Respiratory rate per min	20 (6·5)	19 (5·6)	..	0·59 (−0·79 to 1·97)§; p=0·40
Oxygen saturation	97 (5%)	97 (5%)	..	0·48 (−0·86 to 1·82)§; p=0·48
**Laboratory results (ED arrival)**				
Lactate concentration, mmol/L	7·04 (4·50)	6·93 (4·58)	..	−0·08 (−0·97 to 0·82)§; p=0·87
INR >1·5	12/84 (14%)	12/74 (16%)	0·91 (0·44 to 1·90)*; p=0·80	..
Haemoglobin concentration, g/L	133 (19), 154	118 (23), 152	..	15 (10 to 19)‡; p<0·0001
**Total blood product up to 24 h after ED arrival**				
PRBC	6·34 (7·09), 209	4·41 (6·17), 223	..	1·80 (0·58 to 3·01)‡; p=0·004
Plasma	5·04 (5·56), 209	3·37 (5·04), 223	..	1·54 (0·57 to 2·50)‡; p=0·002
**Death**				
Within 3 h	32/197 (16%)	46/208 (22%)	0·75 (0·50 to 1·13)*; p=0·17	−7% (−15 to 1)†; p=0·08
Within 30 days	86/204 (42%)	99/219 (45%)	0·94 (0·76 to 1·17)*; p=0·59	−4% (−13 to 6)†; p=0·44

Data are n/N (%); mean (SD); median (IQR) participants, unless otherwise specified. Key secondary outcomes are reported here; all secondary outcomes are reported in the appendix (p 102). IDS=intervention delivery site. INR=International normalised ratio. ED=emergency department. LyoPlas=lyophilised plasma. PRBC=packed red blood cells.

* Output is from a log-binomial regression model adjusted for IDS. Values of risk ratio of less than 1 indicate lower event rates in the PRBC–LyoPlas group.

† Output is from a binomial regression model with identity link adjusted for IDS. Values of absolute risk difference of less than 0 indicates lower event rates in the PRBC–LyoPlas group.

‡ Output is a from linear regression model adjusted for IDS. Values of mean differences of less than 0 indicate lower average values the PRBC–LyoPlas group.

§ Output is from a linear regression model adjusted for IDS and the on scene value of the outcome variable. Values of mean differences <0 indicate lower average values in PRBC–LyoPlas group.

Vital signs and lactate concentrations were similar across both groups on arrival at hospital (table 2) through to 24 h (appendix p 102). The mean haemoglobin concentration on arrival to hospital was higher in the PRBC–LyoPlas group compared with the 0·9% sodium chloride group (table 2; appendix p 112). Nine (6%) of 152 participants in the 0·9% sodium chloride group had a haemoglobin <80 g/L at hospital admission. Blood product use was similar after hospital admission up to 24 h. A post-hoc analysis found that total (prehospital and hospital) blood and plasma use was higher in the PRBC–LyoPlas group. Mortality at 3 h and 30 days was not statistically different between groups.

The frequency of adverse events were similar between groups (table 3). The rates of transfusion-related adverse events in the first 24 h after ED arrival were similar across treatment groups: 11 (7%) of 148 in the PRBC–LyoPlas group versus nine (7%) of 137 in the 0·9% sodium chloride group (adjusted relative risk 1·05 [95% CI 0·46–2·42]. Acute respiratory distress syndrome developed in nine (6%) of 142 individuals in the PRBC–LyoPlas group and three (2%) of 130 people in 0·9% sodium chloride group (adjusted relative risk 2·71 [0·75–9·81]); ARDS data was unavailable for one (1%) patient. The number of days organ failure free were also similar across groups: 12·9 (SD 13·0) in the PRBC–LyoPlas group versus 12·1 (13·1) in the 0·9% sodium chloride group (adjusted mean difference 0·86 [95% CI –1·64 to 3·36]; appendix p 105). Two unexpected, unrelated additional serious adverse events were reported one in the PRBC-LyoPlas (cerebral infarct) and one in the 0·9% sodium chloride group (abnormal liver function tests). No patients required dose reductions, had treatment discontinued for drug related toxicity. There were no treatment-related deaths.

**Table 3:** Complications and adverse events

			**Packed red blood cells and lyophilised plasma group (n=142)**	**Sodium chloride group (n=130)**
**Any Organ Failure by system during hospital stay up to day 30 [SOFA ≥3]**				
Respiratory				
	Yes		83 (58%)	68 (52%)
	No		35 (25%)	45 (35%)
	Missing		24 (17%)	17 (13%)
Neurological				
	Yes		89 (63%)	74 (57%)
	No		50 (35%)	56 (43%)
	Missing		3 (2%)	0
Cardiovascular				
	Yes		95 (67%)	80 (62%)
	No		43 (30%)	46 (35%)
	Missing		4 (3%)	4 (3%)
Liver				
	Yes		13 (9%)	6 (5%)
	No		117 (82%)	116 (89%)
	Missing		12 (9%)	8 (6%)
Coagulation				
	Yes		12 (8%)	19 (15%)
	No		123 (87%)	108 (83%)
	Missing		7 (5%)	3 (2%)
Renal				
	Yes		32 (23%)	33 (25%)
	No		104 (73%)	93 (72%)
	Missing		6 (4%)	4 (3%)
Multi-organfailure				
	Yes		86 (61%)	78 (60%)
	No		56 (39%)	52 (40%)
	Missing		0	0
**Acute respiratory distress syndrome**				
Yes			9 (6%)	3 (2%)
No			133 (94%)	126 (97%)
Missing			0	1 (1%)
**Suspicion or clinical evidence of infection**				
Yes			92 (65%)	83 (64%)
No			50 (35%)	47 (36%)
Missing			0 (0%)	0
	Type of Infection*			
		Intra-abdominal	262 (18%)	15 (12%)
		Meningitis	3 (2%)	1 (1%)
		Respiratory	61 (43%)	59 (45%)
		UTI	5 (4%)	10 (8%)
		Soft tissue	35 (25%)	20 (15%)
		Indwelling device	16 (11%)	13 (10%)
		Blood-born	8 (6%)	7 (5%)
		Other	46 (32%)	40 (31%)
**Thromboembolism**				
Yes			17 (12%)	11 (8%)
No			125 (88%)	118 (91%)
Missing			0	1 (1%)
	Type of Thromboembolism*			
		Deep Vein Thrombosis	3 (2%)	3 (2%)
		Pulmonary embolism	9 (6%)	8 (6%)
		Stroke	3 (2%)	0
		Other	2 (1%)	3 (2%)
**Transfusion-related acute lung injury**				
Yes			0	1 (1%)
No			142 (100%)	128 (99%)
Missing			0	1 (1%)
**Transfusion-related complications (in first 24 hours in emergency department)**				
Yes			11 (7%)	9 (7%)
No			137 (93%)	128 (93%)
Missing			0	0

Data are n (%). This list of adverse events and complications are for the 272 participants that completed at least one daily assessment form. PRBC=packed red blood cells. SOFA=sequential organ failure assessment. UTI=urinary tract infection.

* Multiple responses are possible for the type of thromboembolism and type of infection.

## Discussion

This phase 3, multicentre, randomised controlled superiority trial did not demonstrate that prehospital PRBC–LyoPlas resuscitation was superior to 0·9% sodium chloride for trauma related haemorrhagic shock in the civilian population studied. Although the point estimates for the individual components of the primary outcome and some other secondary outcomes (eg, survival within 3 h) are consistent with a benefit from allocation to PRBC–LyoPlas, the confidence intervals are wide and include the possibility of both benefits and harms. The trial found that individuals randomly assigned to the PRBC–LyoPlas group had a higher haemoglobin concentration on admission to hospital and received cumulatively more blood products in total than did people in the control group. The implication is that the logistical and financial costs of bringing blood product resuscitation forward from hospital to the prehospital domain^21^ might not be routinely justified within the context of a modern major trauma network.

Earlier use of blood products during the prehospital phase of trauma care has been driven by the desire to deliver traditionally hospital-based interventions earlier in the patient pathway. Haemostatic resuscitation (ie, transfusion-based resuscitation) has rapidly become the new standard of care for haemorrhagic shock. Past studies have explored the optimal timing and ratio of component therapy. Military and civilian studies suggest that early transfusion improves survival, although the trial evidence remains inconclusive.^16^ There are several explanations as to why RePHILL did not demonstrate benefit from prehospital blood products. The study took place in a civilian setting within an established major trauma network, where prehospital critical care is provided at the scene of the incident by critical care practitioners and doctors. Although the overall time from injury to hospital admission exceeded 1 h on average, a proportion of participants had transport times of less than 20 min, a group that other studies have suggested might not benefit from prehospital transfusion.^22^ RePHILL used lyophilised plasma due to the logistical challenges created by the short post-thaw shelf-life of fresh frozen plasma at the time of the study. Lyophilised plasma has similar or improved biological efficacy relative to fresh frozen plasma.^23^, ^24^ A non-randomised, secondary analysis of the PAMPER trial reported that the use of fresh frozen plasma in combination with packed red cells was beneficial.^25^ Whether the apparent differences relate to the use of lyophilised or fresh frozen plasma or, as the authors of PAMPER highlight, residual confounding in their analyses, will require further research. Our study used a plasma to red cell ratio of 1:1 consistent with UK national guidelines for major haemorrhage.^20^ Whether different ratios, the addition of coagulation factors or platelets, or use of whole blood would have made a difference remains to be determined by future research. Finally, the population of civilian participants enrolled in RePHILL were older, more severely injured, and had a higher proportion of blunt traumatic injuries than in observational military studies, which have suggested benefit from prehospital blood transfusion.^13^, ^11^

The concept of improving oxygen carriage for prehospital trauma patients by the transfusion of red blood cells can be examined given the measured haemoglobin concentration on arrival to hospital. Current European guidance recommends a target haemoglobin measurement of 70–90 g/L before transfusion is instigated, although older patients and people with traumatic brain injury might benefit from higher concentrations of haemoglobin.^26^ Whereas the rate of lactate clearance was not statistically different, the group receiving blood products had an expected higher mean measured haemoglobin concentration on admission to hospital (133 [SD 19]) compared with the sodium chloride group (118 [23]). Of note, only nine (6%) of 152 individuals in the sodium chloride group were admitted with a haemoglobin concentration of <80 g/L. It is noted that the group receiving prehospital blood products received more blood products in total over 24 h than did the sodium chloride group. The extra blood product usage in this group did not appear to improve physiological outcomes or survival.

Frequentist trials approach hypothesis testing in a binary manner, whereby the null hypothesis is accepted or rejected based on probability testing. We included an exploratory Bayesian analysis for the composite primary outcome and its individual components, which enabled us to include a range of previous beliefs about the effectiveness of blood and LyoPlas. Across the range of priors included, the probability that the absolute risk difference was less than 0 (ie, blood and LyoPlas was superior) for the primary outcome was between 44·1–53·4%, for episode mortality was 71·2–88·2%, and for lactate clearance was 81·3–86·9%, although the 95% credible intervals included the possibility for benefit as well as harm. Further research could reduce the current uncertainty about the size and direction of effects for prehospital transfusion.

Although the trial has addressed the original hypothesis, there are several limitations. First, we recruited only 93% of our planned sample size due to the impact of COVID-19. Although it is possible that this might have led to a type 2 error, given the similarity of the primary and secondary outcomes between the intervention and control group, we consider it unlikely that completing recruitment would have led to a substantively different finding. Simulations examining the effect of large increases in sample sizes showed that this would not have materially altered the findings for the primary outcome. Whether a larger trial would have affected the secondary outcomes remains to be determined in future studies. Research in trauma-related haemorrhagic shock is limited by the absence of a core outcome set. After considerable discussions with the research funders, patient and public involvement groups and trial investigators, a composite primary outcome reflecting the efficacy of initial resuscitation (lactate clearance) and overall effectiveness (episode mortality) was chosen. Although there was empirical attractiveness to this combined outcome, the findings in RePHILL were that the direction of effects observed in the individual components for mortality (−3% [95% CI –12 to 7]) and improved lactate clearance (−5% [–14 to 5] in the transfusion group) were not reflected in the composite outcome (−0·025% [–9 to 9]). The suitability of this outcome should be carefully considered for any future studies. The study was necessarily an open-label study, which might have led to performance bias or detection bias, particularly for more subjective outcomes, such as adverse events. Finally, the RePHILL trial used group O, Rhesus factor D negative blood. Greater use of group O Rhesus factor D positive blood, as seen in some international settings, might have reduced demand on the small pool of group O Rhesus factor D negative blood.^27^, ^28^

The study design and delivery of prehospital trials are challenging. Multicentre randomised controlled trials are viewed as the gold standard. Such trials take considerable time to design and deliver and require collaboration to recruit sufficient numbers. Other trials in this area have terminated early due to futility (Control Of Major Bleeding After Trauma [COMBAT])^15^ or insufficient recruitment (Pre-Hospital Use of Plasma for Traumatic Hemorrhage [PUPTH]).^29^ Such randomised clinical trials can also give apparently contradictory results. The PAMPER clinical trial^6^ showed a nearly 30% reduction in mortality with plasma transfusion in the prehospital environment, whereas the COMBAT clinical trial found no evidence of survival improvement. Findings from a post-hoc combined analysis of the 626 participants in both studies suggest that prehospital plasma is associated with a survival benefit when transport times are longer than 20 min.^22^ The ongoing trials of Pre-hospital Administration of Lyophilized Plasma for Post-traumatic Coagulopathy Treatment (PREHO-PLYO, NCT02736812) and Prehospital Plasma or Red Blood Cell Transfusion Strategy in Major Bleeding (PRIEST, NCT04879485) should further inform the value of transfusion in the prehospital setting. Of note, none of these trials include crystalloid as the intervention comparator, despite its widespread use in prehospital care. Future randomised trials should consider exploring the clinical and cost-effectiveness of whole blood, which offers logistical benefits over transfusion of individual components.

The RePHILL trial did not demonstrate that PRBC–Lyoplas improved episode mortality or lactate clearance when compared with 0·9% sodium chloride for participants with trauma-related haemorrhagic shock. Based on current evidence, the decision to commit to routine prehospital transfusion in civilian practice will require careful consideration by all stakeholders.

## Data sharing

Requests for access to data from the RePHILL trial should be addressed to the corresponding author at rephill@trials.bham.ac.uk. The individual participant data collected during the trial (including the data dictionary) will be available, after de-identification, with no end date. All proposals requesting data access will need to specify how the data will be used, and all proposals will need the approval of the trial investigator team before data release.

## Declaration of interests

We declare no competing interests.
