# Supplementary material for: Resuscitation with blood products in patients with trauma-related haemorrhagic shock receiving prehospital care (RePHILL): a multicentre, open-label, randomised, controlled, phase 3 trial
Source: Lancet Haematol. 2022 Mar 7;9(4):e250–61. doi: 10.1016/S2352-3026(22)00040-0 (PMC8960285; doi:10.1016/S2352-3026(22)00040-0)
Supplement: Supplementary appendix [file mmc1.pdf]

THE LANCET
Haematology

Supplementary appendix

This appendix formed part of the original submission and has been peer reviewed. We post it as supplied by the authors.

Supplement to: Crombie N, Doughty HA, Bishop JRB. Resuscitation with blood products in patients with trauma-related haemorrhagic shock receiving prehospital care (RePHILL): a multicentre, open-label, randomised, controlled, phase 3 trial. *Lancet Haematol* 2022; published online March 7. https://doi.org/10.1016/S2352-3026(22)00040-0.

Supplemental material

Table of contents

Trial protocol	Page 1
Statistical analysis plan	Page 59
Further secondary and exploratory outcomes	Page 102
Sensitivity analyses	Page 109
Post-hoc subgroup analyses	Page 110
Intervention delivery sites	Page 111
Haemoglobin concentration on ED arrival	Page 112
RePHILL collaborative group names	Page 113

NIHR | National Institute for Health Research

A Multi-Centre Randomised Controlled Trial of Pre-Hospital Blood Product Administration versus Standard Care for Traumatic Haemorrhage

RePHILL – Resuscitation with Pre-Hospital bLood products

PROTOCOL

Version 3.0, 8th April 2019

**Sponsor:**	**University Hospitals Birmingham NHS Foundation Trust**
**Chief Investigator:**	**Prof. Gavin Perkins**
**Co-Chief Investigator:**	**Dr. Nicholas Crombie**
**Coordinating Centre:**	**Birmingham Clinical Trials Unit**
**Funder:**	**National Institute for Health Research (NIHR) Efficacy and Mechanism Evaluation (EME) Programme**
**EudraCT No.:**	**2015-001401-13**
**ISRCTN:**	**62326938**
**REC Ref. No.:**	**15/SC/0691**

UNIVERSITY OF BIRMINGHAM | BCTU Birmingham Clinical Trials Unit

Surgical Reconstruction and Microbiology Research Centre

AMENDMENTS

The following amendments and/or administrative changes have been made to this protocol since the implementation of the first approved version:

Date of amendment	Protocol version number	Type of amendment	Summary of amendment
18 Jul 2016	1.1	Substantial	• Update of Chief Investigator<br>○ Prof. Mark Midwinter to Prof. Gavin Perkins<br>• Addition of New Sites<br>○ North Bristol NHS Trust (PI: Jason Kendall)<br>○ Norfolk and Norwich University Hospitals NHS Foundation Trust (PI: Frank Sutherland)<br>○ Sheffield Teaching Hospital NHS Foundation Trust (PI: Hasan Qayyum)<br>○ University Hospitals of North Midlands NHS Trust (PI: Thomas James)<br>○ Yorkshire Ambulance Service NHS Trust (PI: Anil Hormis)<br>○ Leeds Teaching Hospital NHS Trust (PI: Jonathan Thornley)<br>○ Hull and East Yorkshire NHS Foundation Trust (PI: Tom Cowlan)<br>○ South Tees NHS Foundation Trust (PI: Jeremy Henning)<br>• Change to Principal Investigators<br>○ University Hospitals Birmingham NHS Foundation Trust (PI: Elaine Hardy)<br>○ East of England Ambulance Service NHS Trust (PI: Tom Davies)<br>○ Cambridge University Hospitals (PI: Alison Hieatt)
21 Sep 2016	1.1	Substantial	• Change of Principal Investigator<br>○ South Tees NHS Foundation Trust (PI: Ian Blain)
25 Jan 2017	2.0	Substantial	• Administrative updates to TMG (formal change to CI requested as part of SA1)<br>• Updates to members of the oversight committees<br>• Clarification on the primary outcome<br>• Update to exclusion criteria<br>• Update to include delivery of interventions by the intraosseous route<br>• Clarification of the informed consent process<br>• Clarification on the randomisation and enrolment process<br>• Update to the schedule of events<br>• Clarification of AE reporting<br>• Clarification on data collection<br>• Statistical updates<br>• Clarification on monitoring requirements<br><br>• Removal of Participating Sites

			○ Yorkshire Ambulance Service NHS Trust (PI: Anil Hormis)<br>○ Hull and East Yorkshire NHS Trust (PI: Tom Cowlam)<br>○ Leeds Teaching Hospital NHS Trust (PI: Jonathan Thornley)<br>○ South Tees Hospital NHS Foundation Trust (PI: Ian Blain)<br>○ East of England Ambulance Service NHS Trust (PI: Tom Davies)<br>• Change of Principal Investigator<br>○ Cambridge University Hospitals (PI: Sarah Hazelman)<br>○ Sheffield Teaching Hospitals NHS Foundation (PI: Gary Mills)
6 Feb 2017	2.0	Substantial	• Addition of Participating Site<br>○ The Air Ambulance Service (PI: Caroline Leech)
16 Feb 2017	2.0	Substantial	• Addition of Participating Site<br>○ MAGPAS Air Ambulance (PI: Simon Lewis)
16 Mar 2017	2.0	Substantial	• Addition of Participating Site<br>○ Barts Health NHS Trust (PI: Tim Harris)<br>• Change of Principal Investigator<br>○ University Hospitals Birmingham NHS Foundation Trust (PI: David Yeo)
05 May 2017	2.0	Substantial	• Change of Principal Investigator<br>○ West Midlands Air Ambulance (PI: Mark Nash)
24 Aug 2017	2.0	Substantial	• Addition of Participating Site<br>○ East Anglian Air Ambulance (PI: Alistair Wilson)
22 Dec 2017	2.0	Substantial	• Change of Principal Investigator<br>○ Norfolk and Norwich University Hospitals NHS Foundation Trust (PI: Meenal Galal)
08 Jun 2018	2.0	Substantial	• Change of Principal Investigator<br>○ University Hospitals of North Midlands NHS Trust (PI: Philip Morgan)
14 Jun 2018	2.0	Substantial	• Change of Principal Investigator<br>○ Barts Health NHS Trust, (PI: Benjamin Bloom)
30 Jul 2018	2.0	Substantial	• Addition of Participating Site<br>• Luton and Dunstable Hospital (PI: Manoj Viegas)<br>• Change of Principal Investigator<br>○ Cambridge University Hospitals (PI: Adam Chesters)
12 Oct 2018	2.0	Substantial	• Addition of Participating Site<br>○ John Radcliffe Hospital, Oxford University Hospitals NHS trust (PI: Aqib Hafeez)<br>• Change of Principal Investigator<br>○ University Hospitals of North Midlands NHS Trust (PI: Thomas James)
01 Feb 2019	2.0	Substantial	• Change of Principal Investigator<br>○ Norfolk and Norwich University Hospitals NHS Foundation Trust (PI: Francoise Sheppard)
18 Feb 2019	3.0	Substantial	• Removal of participating sites table

			• Update to secondary outcomes<br>• Update to who will assess and confirm eligibility<br>• Update to the exclusion criteria<br>• Removal of NHS digital, long term follow-up<br>• Clarification to trial procedure on-scene<br>• Clarification of informed consent procedure<br>• Update to blood sampling<br>• Removal of blood sampling for future analysis<br>• Update to pharmacovigilance reporting requirements<br>• Update to categorisation of causality table<br>• Update to of data protection regulations<br>• Update to end of trial definition

Trial Management Group	
**Clinical Team**	
**Chief Investigator:**	
Prof. Gavin Perkins	**Professor in Critical Care Medicine**<br>Warwick Medical School and University Hospitals Birmingham NHS Foundation Trust<br>Email: g.d.perkins@warwick.ac.uk
**Co-Chief Investigator:**	
Dr. Nicholas Crombie	**Consultant Trauma Anaesthetist**<br>University Hospitals NHS Foundation Trust<br>Email: nicholas.crombie@uhb.nhs.uk
Mr. Iain Smith	**Speciality Registrar in General Surgery (Co-Investigator)**<br>Queen Elizabeth University Hospital, Glasgow<br>Email: iain.smith6@nhs.net
Dr. Heidi Doughty	**Consultant in Transfusion Medicine (Co-Investigator)**<br>NHS Blood & Transplant<br>Email: heidi.doughty@nhsbt.nhs.uk
Major David Naumann	**Research Fellow**<br>University of Birmingham and University Hospitals Birmingham NHS Foundation Trust<br>Email: david.naumann@nhs.net
Miss Hazel Smith	**Research Paramedic**<br>University Hospitals Birmingham NHS Foundation Trust<br>Email: hazel.smith18@nhs.net
**University of Birmingham Clinical Trials Unit (BCTU)**	
**Statisticians:**	
Miss Natalie Ives	**Reader in Clinical Trials, Statistics Team Leader (Co-investigator):**<br>Email: n.j.ives@bham.ac.uk
Dr. Jon Bishop	**Senior Statistician (Co-investigator):**<br>Email: j.bishop.1@bham.ac.uk
**Trial Management:**	
Dr. Margaret Grant	**Director of Operations and Trials Management:**<br>Email: m.r.grant@bham.ac.uk
Miss Gemma Slinn	**Trials Management Team Leader:**<br>**Email:** g.slinn@bham.ac.uk
Dr. Rebekah Wale	**Trial Manager**<br>**Email: r.wale@bham.ac.uk**

Trial Steering Committee	
**Chair:**	
Prof. Ian Roberts	Professor of Epidemiology & Public Health, Co-director of the Clinical Trials Unit at the London School of Hygiene & Tropical Medicine<br>Email: ian.roberts@lshtm.ac.uk
**Independent Members:**	
Prof. John Holcomb	Professor of Surgery, University of Texas<br>Email: john.holcomb@uth.tmc.edu
Dr. Simon Stanworth	Consultant Haematologist, NHS Blood & Transplant<br>Email: simon.stanworth@nhsbt.nhs.uk
Prof. Jason Smith	Honorary Professor, Plymouth University Peninsula Schools of Medicine and Dentistry<br>Email: jasonesmith@nhs.net
Prof. Timothy Coats	Professor of Emergency Medicine, Leicester University<br>Email: tc61@le.ac.uk
**Lay Members:**	
Mr. Andrew Cox	Queen Elizabeth Hospital Birmingham, Trauma PPI Group<br>Email: e-cox@sky.com
Mr. Timothy Marshall	Queen Elizabeth Hospital Birmingham, Trauma PPI Group<br>Email: timmarshall666@hotmail.com
**On behalf of the TMG:**	
Prof. Gavin Perkins<br>Dr. Nicholas Crombie	See Trial Management Group for contact details

Data Monitoring and Ethics Committee	
**Chair:**	
Prof. Jon Nicholl	Professor of Health Services Research, School of Health and Related Research, University of Sheffield<br>Email: j.nicholl@sheffield.ac.uk
Dr. Jan Jansen	Associate Professor of Surgery & Director of Research, Division of Acute Care Surgery, University of Alabama at Birmingham<br>Email: jjansen@uabmc.edu
Prof. Fiona Lecky	Clinical Professor of Emergency Medicine, University of Sheffield<br>Email: f.e.lecky@sheffield.ac.uk

RePHILL Trial Office

For general protocol related queries and supply of trial materials:

Birmingham Clinical Trials Unit (BCTU), College of Medical and Dental Sciences, Public Health Building, University of Birmingham, Edgbaston, Birmingham, B15 2TT

Telephone: 0121 414 7943 Fax: 0121 415 9135
Email: RePHILL@trials.bham.ac.uk

Clinical Queries

Co-Chief Investigator: Dr. Nicholas Crombie (nicholas.crombie@uhb.nhs.uk)

Research Paramedic: Miss Hazel Smith (hazel.smith18@nhs.net)

or telephone 07789933031

Intervention Box Allocation

Telephone: 0800 953 0274

Database: https://www.trials.bham.ac.uk/RePHILL/

Participant Enrolment

Database: https://www.trials.bham.ac.uk/RePHILL/

Safety Reporting

Fax SAE Forms to: 0121 415 9135

Chief Investigator and Sponsor Signatures

The Chief Investigator and Sponsor have discussed this protocol and agree to abide by this protocol and to conduct the trial in compliance with EU Good Clinical Practice (GCP), the applicable UK Statutory Instruments, which include the Medicines for Human Use (Clinical Trials) Regulations 2004 and subsequent amendments, the current UK recommended data protection regulations including the Data Protection Act (2018), the Trust Information Governance Policy (or local equivalent) and the UK Policy Framework for Health and Social Care Research

Chief Investigator

Prof. Gavin Perkins

____

Signature Date

Sponsor Representative

Dr Chris Counsell

____

Signature Date

Principal Investigator Signature Page

Principal Investigator:

I have read and agree to the protocol, as described in this document. I agree to adhere to the protocol as outlined and agree that any suggested changes to the protocol must be approved by the Trial Steering Committee (TSC) prior to seeking approval from the Research Ethics Committee (REC).

I am aware of my responsibilities as an Investigator under the guidelines of Good Clinical Practice (GCP), the Declaration of Helsinki and the trial protocol and I agree to conduct the trial according to these guidelines and to appropriately direct and assist the staff under my control, who will be involved in the trial.

Principal investigator

<insert name>

Signature Date

Name of Institution

<insert name>

The Principal Investigator should sign this page and return a copy to the RePHILL Trial Office

Abbreviations

AE	Adverse Event
aPTT	Activated Partial Thromboplastin Time
AR	Adverse Reaction
ARDS	Acute Respiratory Distress Syndrome
ASR	Acute Safety Report
ATR	Annual Transfusion Reaction
BCTU	Birmingham Clinical Trials Unit at the University of Birmingham
CI	Chief Investigator
CPAP	Continuous Positive Airway Pressure
CRF	Case Report Form
CRPD	Clinical Practice Research Datalink
CTA	Clinical Trial Authorisation
DAT	Direct Antigen Test
DIBD	Developmental International Birth Date
DMEC	Data Monitoring and Ethics Committee
DSUR	Development Safety Update Report
ED	Emergency Department
EudraCT	European Clinical Trials Database
FBC	Full Blood Count
FFP	Fresh Frozen Plasma
GCP	Good Clinical Practice
GP	General Practitioner
HES	Hospital Episode Statistics
HSCIC	Health & Social Care Information Centre
HR	Haemostatic Resuscitation
ICF	Informed Consent Form
IDS	Intervention Delivery Site
IMP	Investigational Medicinal Product
INR	International Normalised Ratio
IO	Intraosseous
ISF	Investigator Site File
ISRCTN	International Standard Randomised Controlled Trial Number
IV	Intravenous
kPa	KiloPascals

MCMC	Marcov chain Monte Carlo
MHRA	Medicines and Healthcare Products Regulatory Authority
NIHR	National Institute for Health Research
NIRS	Near-Infra-Red Spectroscopy
NHSBT	NHS Blood & Transplant
ONS	Office of National Statistics
PEEP	Positive End Expiratory Pressure
PRBC	Packed Red Blood Cells
PHBP	Pre-Hospital Blood Products
PHEM Team	Pre-Hospital Emergency Medical Team
PI	Principal Investigator – the local lead investigator for the RePHILL Trial
PIS	Participant Information Sheet
PT	Prothrombin Time
RCT	Randomised Controlled Trial
REC	Research Ethics Committee
RHS	Receiving Hospital Site
ROTEM®	Rotational Thromboelastometry
SABRE	Serious Adverse Blood Reactions and Events
SAE	Serious Adverse Event
SAP	Statistical Analysis Plan
SAR	Serious Adverse Reaction
SBP	Systolic Blood Pressure
SHOT	Serious Hazards of Transfusion
SOFA score	Sequential Organ Failure Assessment score
SOP	Standard Operating Procedure
SmPC	Summary of Product Characteristics
SRMRC	Surgical Reconstruction and Microbiology Research Centre
SUSAR	Suspected Unexpected Serious Adverse Reaction
TIC	Trauma Induced Coagulopathy
TMG	Trial Management Group
TSC	Trial Steering Committee
UK	United Kingdom
U&Es	Urea and Electrolytes
vCJD	variant Creutzfeldt-Jakob Disease

1. TRIAL SUMMARY 16

2. BACKGROUND AND RATIONALE 19

2.1 Existing Research and Current Practice 19

2.2 Clinical Studies 20

2.3 Trial Rationale 21

2.4 Risks and Benefits 21

2.5 Assessment and Management of Risk 21

3. TRIAL OBJECTIVES AND OUTCOME MEASURES 22

3.1 Trial Objectives 22

3.1.1 Principle Objective 22

3.1.2 Secondary Objectives 22

3.2 Internal Pilot Trial 22

3.3 Outcome Measures 23

3.3.1 Primary Outcome 23

3.3.2 Secondary Outcomes 23

4. ELIGIBILITY 24

4.1 Inclusion Criteria 24

4.2 Exclusion Criteria 24

5. INFORMED CONSENT PROCEDURE 24

5.1 Participant Consent (*after* trial intervention) 26

5.2 Participants Who Lack Capacity to Consent for Themselves 26

5.3 Consent Arrangements for Participants under the Age of 16 27

5.4 Participants Who Do Not Survive 27

5.5 28

Participants transferred to non-RePHILL Hospitals 28

6 RANDOMISATION AND ENROLMENT PROCESS 29

6.1 Randomisation Process 29

6.1.1 Role of Blood Banks 29

6.1.2 Role of PHEM 29

6.2 Randomisation 30

6.3 Enrolment....................30

6.4 Co-enrolment....................30

6.5 Post Randomisation Exclusions and Withdrawals 30

7. TRIAL PROCEDURES AND ASSESSMENTS 31

7.1 On-scene....................31

7.1.1 Subsequent Boluses....................32

7.1.2 Lactate Concentration 32

7.2 On Arrival at the Receiving Hospital ED 32

7.2.1 Vital Signs 32

7.2.2 Tissue Oxygenation and Perfusion 32

7.2.3 (Venous) Lactate Concentration 32

7.2.4 Calculating Lactate Clearance 33

7.2.5 Blood Samples on Admission....................33

7.2.6 Blood Sampling for ROTEM®....................33

7.2.7 Blood Sampling for Platelet Function....................33

7.3 During Hospital Admission....................34

7.3.1 Acute Respiratory Distress Syndrome (ARDS)....................34

7.3.2 Sequential Organ Failure Assessment score (SOFA) score 34

7.4 Schedule of Assessments 36

8. TRIAL INTERVENTION/INVESTIGATIONAL MEDICINAL PRODUCTS 37

8.1 Trial Treatments....................37

8.1.1 PHBP Arm (Lyophilised Plasma LyoPlas N-w (LyoPlas) and PRBC) 37

8.1.2 Crystalloid Arm 37

8.2 Supply of Trial Stocks and Storage Conditions 37

8.2.1 Trial Supplies 37

8.2.2 Packaging and Labelling of the IMPs 38

8.2.3 Storage 38

8.3 Administration of Treatment....................38

8.4 Interactions or Contraindications....38

9. PHARMACOVIGILANCE.... 39

9.1 Reporting Requirements....39

9.2 (Serious) Adverse Events 40

9.2.1 Events that do not Require Reporting on a SAE Form....40

9.2.2 Monitoring Participants Pregnancies for Potential SAEs 41

9.2.3 Reporting Period....41

9.3 Reporting Procedure – Site 41

9.3.1 Serious Adverse Events 41

9.3.2 Provision of Follow-up Information 42

9.4 Reporting Procedure – Trial Office....42

9.5 Reporting to the Competent Authority and Research Ethics Committee....43

9.5.1 Suspected Unexpected Serious Adverse Reactions....43

9.5.2 Serious Adverse Reactions....43

9.5.3 Other Safety Issues Identified during the Course of the Trial....43

9.6 Investigators....43

9.7 Haemovigilance....43

9.8 Developmental Safety Update Reports 44

9.9 Annual Progress Reports 44

9.10 Reporting Urgent Safety Measures 44

9.11 Notification of Serious Breaches of Good Clinical Practice and/or the Protocol...44

10. DATA MANAGEMENT AND QUALITY ASSURANCE.... 45

10.1 Confidentiality 45

10.2 Data Collection 45

11. STATISTICAL CONSIDERATIONS.... 46

11.1 Sample Size 46

11.2 Analysis of Outcome Measures....47

11.2.1 Primary Outcome Analysis....48

11.2.2 Secondary Outcome Analysis....48

11.2.3 Subgroup Analyses 48

11.2.4 Missing Data and Sensitivity Analyses 49

11.3 Planned Interim Analyses 49

11.4 Planned Final Analyses....49

12. END OF TRIAL 49

13. ARCHIVING.... 49

14. DIRECT ACCESS TO SOURCE DATA 50

15. ETHICS AND REGULATORY REQUIREMENTS 50

16. MONITORING REQUIREMENT.... 51

17. OVERSIGHT COMMITTEES 51

17.1 Trial Management Group 51

17.2 Trial Steering Committee (TSC) 51

17.3 DMEC....51

18. FINANCE 52

19. INDEMNITY 52

20. DISSEMINATION AND PUBLICATION 54

1. Trial Summary

**Title**	**A Multi-Centre Randomised Controlled Trial of Pre-Hospital Blood Product Administration versus Standard Care for Traumatic Haemorrhage**
**Acronym**	**RePHILL**
**Trial Design**	A multicentre randomised controlled, open-label, parallel group two arm trial with internal pilot.
**Aim**	This trial will test the hypothesis that Pre-Hospital Blood Products (PHBP) resuscitation with up to two units each of packed red blood cells (PRBC) and lyophilised (freeze-dried) plasma (LyoPlas N-w which will be referred to as LyoPlas) will improve tissue perfusion (as measured by lactate clearance) and reduce mortality in trauma participants with haemorrhagic shock compared to the current standard practice of crystalloid resuscitation.<br>The trial includes an internal pilot phase (25 participants) which will test logistical aspects of the trial and assess feasibility and recruitment.
**Total number participants**	490 (inclusive of pilot phase)
**Planned trial sites**	Intervention Delivery Sites (IDS) and Receiving Hospital Sites (RHS).
**Main inclusion and exclusion criteria**	**Inclusion criteria:**<br>• Traumatic injury<br>• Pre-Hospital Emergency Medical team attend<br>• Hypotension Systolic Blood Pressure <90mmHg or absence of palpable radial pulse) believed to be due to traumatic haemorrhage.<br>**Exclusion Criteria:**<br>• Children (known or apparently aged <16 years)<br>• Blood administered on-scene, prior to randomisation<br>• Traumatic cardiac arrest where a) the arrest occurred prior to arrival of the PHEM team and/ or b) the primary cause is not hypovolaemia<br>• Refusal of blood product administration (e.g. known Jehovah's Witness)<br>• Pregnancy (known or apparent)<br>• Isolated head injury without evidence of external haemorrhage<br>• Known prisoners in the custody of HM Prison or Probation services

**Outcome measures**	**Primary outcome:**<br>Composite measure consisting of:<br>• Episode mortality[I]<br>• Lactate clearance. A failure to achieve lactate clearance ≥ 20% per hour in the first 2 hours from randomisation[II]<br><br>**Secondary outcomes:**<br>• Individual components of the primary outcome<br>• All-cause mortality within 3 hours of randomisation<br>• Pre-hospital time and type and volume of fluid<br>• Vital signs (systolic blood pressure, heart rate, capillary oxygen saturation)<br>• (Venous) lactate concentration<br>• Haemoglobin concentration on ED arrival<br>• Trauma-induced coagulopathy (defined as International Normalised Ratio (INR) >1.5)<br>• Coagulation measured viscoelastically by rotational thromboelastometry (ROTEM®)[III]<br>• Platelet function using multiple electrode impedence aggregometery (MultiPlate)[III]<br>• Total blood product receipt<br>• Acute respiratory distress syndrome<br>• Transfusion-related complications<br>• Organ failure-free day
**Trial duration per participant**	Main trial data collection ends at withdrawal, acute care discharge, death or at 30 days follow-up, whichever occurs first. Apart from episode mortality data which will be collected up to discharge from the acute care setting, which may be >30 days.

[I] Episode mortality refers to mortality between time of injury/ recruitment and up to discharge from the primary receiving facility to non-acute care, i.e. discharge home or to long-term care, to rehabilitation or repatriation to a hospital closer to their normal residence

[II] A participant is considered randomised and entered into the trial when the first intervention box has been opened.

[III] Selected RHS only

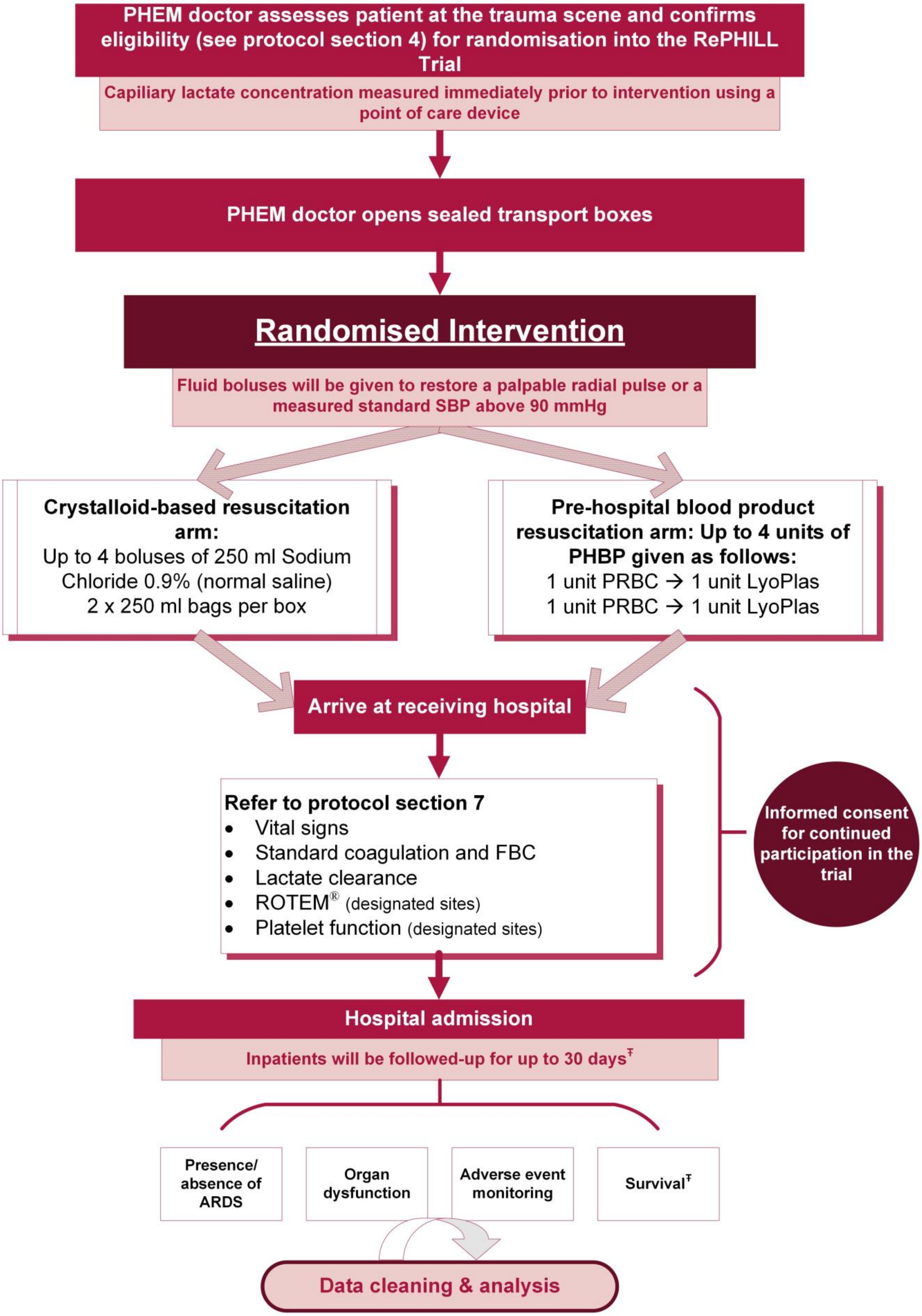

[Ŧ] Episode mortality data will be collected up to discharge from the acute care setting which may be >30 days

Appendix page 18

2. Background and Rationale

2.1 Existing Research and Current Practice

The administration of high ratios of plasma to packed red blood cells (PRBC) has been widely adopted for in-hospital treatment of major traumatic haemorrhage. The rationale is to provide "haemostatic resuscitation" (HR) to address trauma induced coagulopathy (TIC), which carries a fourfold increase in mortality. Evidence for HR is almost exclusively derived from observational studies. A recent Cochrane review identified no randomised controlled trials (RCTs) of plasma-based trauma resuscitation[1], while a previous systematic review found only one outdated blood component RCT of platelets[2]. The recently published Pragmatic Randomized Optimal Platelet and Plasma Ratios trial found no difference in mortality between two transfusion ratio regimens[3], both of which would be considered "haemostatic resuscitation" when compared to conventional approaches.

The only adequately performed RCT of pre-hospital fluids found that aggressive crystalloid administration increased mortality and morbidity after penetrating trauma[4]. Underlying mechanisms are believed to include increased blood pressure "blowing-off" immature clot, leading to re-bleeding. Consequently, restricted fluid regimes became standard pre-hospital care. A separate attempt to examine pre-hospital intravenous (IV) fluid resuscitation in trauma was inconclusive, with over half of the participants receiving the wrong intervention[5].

Acceptance of in-hospital HR saw the British military implement it for battlefield casualty retrieval [6]. Initially two units cells PRBC and two units of thawed plasma were carried, later increasing to four units of each. Civilian pre-hospital retrieval services have adopted a limited version of this practice, carrying PRBC alone for trauma resuscitation[7-10]. This increases demand for universal donor red cells in the absence of robust supporting evidence. Although intuitively blood product replacement should be beneficial to trauma patients, similarly logical interventions for bleeding such as recombinant activated Factor VII[11] and pneumatic anti-shock garments[12] have failed to demonstrate benefit when formally tested in randomised trials.

Implementation of the British military version of pre-hospital HR has not been possible in the UK due to logistic constraints. Thawed plasma is unsuitable for UK civilian practice due to its limited post-thaw shelf-life (24 hours) and the rarity of exsanguinating trauma, which would lead to significant product wastage. The 15 month shelf-life of LyoPlas makes it an attractive alternative, but it has not to date been tested in an RCT.

The Prehospital Air Medical Plasma (PAMPer) study (a four-year RCT which started in 2014 in the USA)[13] compares two units of thawed plasma against conventional care. PAMPer will not assess coagulopathy, nor provide information about the role of packed red cells. RePHILL will address both

of these in a different trauma population - one dominated by blunt mechanisms with a far lower incidence of gunshot wounds.

2.2 Clinical Studies

Meta-analysis of observational studies of in-hospital HR suggests increased survival[14, 15]. However, the minimal pre-hospital evidence is inconsistent. Consultant-delivered battlefield casualty retrieval (with access to pre-hospital blood products (PHBP) including thawed plasma) was associated with reduced mortality in major, but sub-catastrophic injuries (Injury Severity Score between 16 and 50)[16]. However, only 32% of such patients received PHBP, while 41% received advanced airway interventions, 25% received chest decompression and 60% and 46% received IV or interosseous access respectively. The study could not determine the cause of the improved survival.

A matched cohort study of casualties with similar injuries before and after the introduction of PHBP found that PHBP-recipients had 8% mortality vs. 20% in non-recipients[17]. However, pre-hospital times were longer prior to introduction of PHBP, non-recipients had greater physiological derangement and more than 50% of non-recipients received no blood products after hospital arrival, compared to median transfusions of 2 units each of red blood cells and plasma amongst PHBP-recipients. The "PHBP era" coincided with increasingly liberal in-hospital transfusions[18] and many clinicians deployed during the PHBP period had experience gained from previous deployments before PHBP were available. The only prospective cohort study to date is less favourable. Transport by civilian air ambulance with PHBP was associated with reduced 6-hour mortality compared to patients transported by an air ambulance without PHBP. Overall mortality was similar[8]. An older civilian study reported that in-flight blood receipt was associated with greater acidosis at hospital arrival than crystalloid resuscitation, but was confounded by much longer flight times in the blood recipients[19], while a recently completed case-control study of 1047 battlefield casualties found no reduction in coagulopathy or mortality from PHBP even after multivariate regression[20]. The most persuasive evidence in favour of PHBP is a retrospective study in which 50/1415 (3.5%) of blunt trauma patients received pre-hospital PRBC, with a 64% reduction in hazard ratio of 30-day mortality[21]. However, 48% of PRBC recipients were interfacility transfers rather than primary retrievals from scene (vs. 4% of non-recipients), thus survivorship bias may have influenced the results. Absolute mortality was higher amongst PHBP recipients in both the overall study and matched subgroup analysis (Brown, J., pers. comm, 08 June 2015). Our recently completed systematic review found no "moderate" or "good" quality evidence supporting PHBP resuscitation, and meta-analysis of the limited (and entirely observational) data showed no evidence of a long-term survival benefit[22].

2.3 Trial Rationale

With the increasing adoption of PHBP resuscitation for trauma in both the UK and abroad, in both military and civilian settings, it is important to determine whether this intervention is, in fact, effective. The logistical and financial resources required for the provision of PHBP resuscitation are significant and require dedicated use of valuable universal donor blood components. This trial is an opportunity to establish a robust evidence base for PHBP resuscitation; an opportunity, which may fade if the trend for PHBP continues to the point that equipoise is lost despite a lack of high quality evidence, as is the case for in-hospital HR.

2.4 Risks and Benefits

The risks to participants in this trial are considered to be minimal. PHBP resuscitation delivers an equivalent intervention to participants which they would inevitably have received on arrival in hospital. The same single donor derived LyoPlas which will be used in this trial is established in the German and Israeli Defence Forces Medical Corps[23], while 10-donor mini-pool derived LyoPlas is used by the French military[24] with no reports of significant adverse events[25], though this product is not commercially available. LyoPlas N-w is produced by a quarantined single donor process – plasma is only processed if a donor has unremarkable infectivity testing at least four months after the donation was received. Plasma is then filtered, rendering it virtually cell-free. To minimise risk of transfusion-related acute lung injury, LyoPlas is only produced from leucocyte-antibody negative plasma. Transmission of prion disease (variant Creutzfeldt-Jakob Disease; vCJD) is not considered a hazard of this study – as of June 2014, no cases of vCJD have been reported in Germany[26]. Although German plasma does not meet full vCJD risk criteria for NHS Blood & Transplant (NHSBT) importation under all modelling conditions, the worst-case scenario is that if all fresh frozen plasma (FFP) requirements for patients born after 01 Jan 1996 were met from German sources, 0.1 clinical cases would result (1.9 log reduction compared to UK sourced plasma)[27]. As the majority of the approximately 245 plasma recipients in this study will have been born prior to 1996 (and would receive UK-sourced plasma in routine clinical practice), the additional risk of vCJD transmission from LyoPlas N-w use is considered to approach zero.

In contrast, massive traumatic haemorrhage leading to profound hypotension (systolic blood pressure (SBP) <90mmHg) is associated with 23% mortality[28, 29]. Any benefit from PHBP is potentially lifesaving.

2.5 Assessment and Management of Risk

The assessment and management of risk is detailed in the separate RePHILL Risk Assessment document. An on-going evaluation of risk will continue throughout the trial.

3. Trial Objectives and Outcome Measures

3.1 Trial Objectives

3.1.1 Principle Objective

The principle objective of this trial is to investigate the clinical effectiveness of PHBP resuscitation compared to the current standard care of restricted crystalloid based resuscitation in participants suffering from major traumatic haemorrhage.

3.1.2 Secondary Objectives

To test the hypotheses that, when compared to standard care, does PHBP resuscitation:

I. Improve blood pressure, heart rate and capillary oxygenation on ED arrival?

II. Prolong on-scene time?

III. Reduce pre-hospital fluid requirements?

IV. Reduce in-hospital transfusion requirements?

V. Reduce trauma-induced coagulopathy?

VI. Preserve platelet function?

VII. Lead to a greater incidence of transfusion-related complications, particularly acute respiratory distress syndrome?

VIII. Lead to blood product wastage?

IX. Affect haemoglobin concentration levels on ED arrival?

3.2 Internal Pilot Trial

The first 6 months of the RePHILL trial will constitute an internal pilot to assess and confirm the trial logistics to determine if it is both feasible and practical to carry on and recruit into the trial. The pilot will be run at multiple sites to validate the multi-centre aspects of the trial.

At the end of the pilot phase, the following targets should be met to justify progression to the main trial:

- Minimum of 25 participants recruited across at least two active sites;
- In participants recruited to the trial intervention arm, at least one unit of PRBC and one unit of LyoPlas delivered to at least 80% of participants before reaching hospital
- At least 90% complete data capture
- Data Monitoring and Ethics Committee (DMEC) reports no safety concerns, which would prohibit continuation to main trial.

3.3 Outcome Measures

3.3.1 Primary Outcome

The primary outcome is a composite measure consisting of:

- Episode mortality[IV]
- Lactate clearance. A failure to achieve lactate clearance ≥ 20% per hour in the first 2 hours after randomisation[V]

3.3.2 Secondary Outcomes

- Individual components of the primary outcome
- All-cause mortality within 3 hours of randomisation
- All-cause mortality within 30 days of randomisation
- Pre-hospital time and type and volume of fluid
- Vital signs (systolic blood pressure, heart rate, capillary oxygen saturation) at scene, on arrival at the Emergency Department (ED), then also at 2, 6, 12 and 24 hours after arrival at ED
- Haemoglobin concentration on ED arrival
- (Venous) lactate concentration on arrival at ED and at 2 hours after arrival at ED
- Trauma-induced coagulopathy (defined as International Normalised Ratio (INR) >1.5) to be measured on arrival at ED, and also at 2 and 6 hours after arrival at ED
- Coagulation measured viscoelastically by rotational thromboelastometry (ROTEM®)[VI]
- Platelet function using multiple electrode impedence aggregometery (MultiPlate)[VI]
- Total blood product receipt at 6, 12 and 24 hours after arrival at ED
- Acute respiratory distress syndrome (ARDS) within the first 7 days after injury
- Transfusion-related complications
- Organ failure-free days[30]. The presence of organ failure is defined as any Sequential Organ Failure Assessment (SOFA) component score[31] of ≥ 3. Organ failure will be assumed to be absent if the participant is discharged from hospital and will be assumed to be present if the participant has died

[IV] Episode mortality refers to mortality between time of injury/ recruitment and discharge from the primary receiving facility to non-acute care, i.e. discharge home or to long-term care, to rehabilitation or repatriation to a hospital closer to their normal residence

[V] A participant is considered randomised and entered into the trial when the first intervention box has been opened.

[VI] Selected RHS only

4. Eligibility

The Pre-Hospital Emergency Medical (PHEM) team (doctor and/or paramedic) will assess the potential participant's vital signs on-scene and confirm that they are eligible to be entered into the RePHILL trial. In all cases, the PI for Intervention Delivery Sites (IDS) will be a medically qualified doctor and they will be responsible for maintaining oversight of the confirmation of eligibility process.

4.1 Inclusion Criteria

- Traumatic injury
- Pre-Hospital Emergency Medical team attend
- Hypotension (Systolic Blood Pressure <90mmHg or absence of palpable radial pulse) believed to be due to traumatic haemorrhage

4.2 Exclusion Criteria

- Children (known or apparently aged <16 years)
- Blood administered on-scene, prior to arrival of the RePHILL PHEM team
- Traumatic cardiac arrest where a) the arrest occurred prior to arrival of the PHEM team and/ or b) the primary cause is not hypovolaemia
- Refusal of blood product administration; known Jehovah's Witness
- Pregnancy (known or apparent)
- Isolated head injury without evidence of external haemorrhage
- Known prisoners in the custody of HM Prison or Probation services

5. Informed Consent Procedure

Major traumatic haemorrhage is a life-threatening condition that requires urgent treatment. RePHILL is a trial of a potentially life-saving intervention. The vast majority of eligible participants will lack capacity throughout the recruitment and intervention periods of the trial. An occasional participant may retain capacity; however, their clinical condition will require immediate resuscitation. It would be inappropriate to attempt to gain informed consent at this time, as it would delay life-saving resuscitation. It is therefore impossible to obtain prospective informed consent. It would also be clinically unjustifiable to delay treatment until full informed consent can be obtained from a personal legal representative. Even if such a representative were immediately available, the emotional distress of the situation is such that they would be unlikely to make an informed decision in the minimal time available. Consequently, RePHILL **cannot be conducted on the basis of prospective informed consent**.

Participants who are incapable of giving consent in emergency situations are an established exception to the general rule of informed consent in clinical trials. This is clearly acknowledged in the Declaration of Helsinki 2008). Under UK law, emergency care is permitted under the terms of The Medicines for Human Use (Clinical Trials; Amendment No.2) Regulations 2006. Specifically:

- *Having regard to the nature of the trial and the particular circumstances of the case, it is necessary to take action for the purpose of the trial as a matter of urgency*
- *It is not reasonably practicable to obtain informed consent prior to entering the subject*

  (Due to the extreme physiological compromise which will be present in eligible participants, it is not practical to seek informed consent as to do so would delay resuscitation and increase the risk to the potential participant's life)
- *The action to be taken is carried out in accordance with a procedure approved by the research ethics committee*

The Pre-Hospital Emergency Medical (PHEM) team will search the participant on scene for evidence that they would refuse participation, such as an Advance Directive, as carried by members of the Jehovah's Witness faith.

Contact with trial participants and/or their relatives/friends to initiate the consent process will be made as soon as practically possible after the initial emergency has passed, taking the utmost care and sensitivity in doing so. Based on findings from previous trauma research studies and from engaging with patient and public representatives it has been suggested that the earliest practicable time to make contact is once the participant is no longer critically ill.

Details of the informed consent discussions will be recorded in the participant's medical notes. This will include date of discussion, the name of the trial, summary of discussion, version number of the participant information sheet (PIS) given to the participant or their legal representative, version number of the informed consent form (ICF) , what type of consent was received (legal representative and/or participant), and that the ICF was signed and dated.

Throughout the follow-up period, the participant's willingness to continue in the trial will be ascertained (through the participant themselves, or their legal representative as appropriate) and documented in the medical notes, and the participant or legal representative will have the opportunity to ask questions about the trial. Any new information that may be relevant to the participant's continued participation will be provided. Where new information becomes available which may affect the decision to continue, participants or their legal representative will be given time to consider and if happy to continue will be re-consented. Re-consent will be documented in the medical notes. The participant's right to withdraw from the trial will remain.

Appendix page 25

Electronic copies of the PIS and ICF will be available from the Trial Office and will be presented on the headed paper of the local institution. With the participant's prior consent, their General Practitioner (GP) will also be informed that they are taking part in the trial.

5.1 Participant Consent (*after* trial intervention)

The local research team at the receiving hospital will assess if the participant has capacity to consent for themselves. If the participant does have capacity, they will be provided with the Research Ethics Committee (REC) approved PIS explaining the trial and the options of their continued involvement. The participant will be given time to consider all of the information, have the opportunity to ask questions and discuss with others. A member of the local research team will ask the participant when they would like someone to come back to discuss participation further and potentially receive consent.

The participant may decide that it is not an appropriate time to discuss the trial or they may decide upfront that they do not want to be involved in which case, their feelings will be respected and their decision about continuing in the trial will be recorded.

5.2 Participants Who Lack Capacity to Consent for Themselves

Consent from a **legal representative** will be sought as soon as practically possible; with the recommendation being that this is obtained within the first 72 hours of the participant's hospital admission.

In the first instance, the local research team will work to identify a **personal legal representative** as defined below:

A personal legal representative is a person independent of the trial, who by virtue of their relationship with the trial participant is suitable to act as their legal representative for the purposes of the trial and who is available and willing to act for those purposes.

The personal legal representative will be approached and will be provided with the REC approved personal legal representative information sheet explaining the trial and the options for the participant's continuing involvement, including the need for them to give consent on behalf of the participant. The personal legal representative will then have time to consider the information provided, after which, a member of the local research team will ask when the personal legal representative would like them to come back and discuss participation further and potentially receive consent.

The personal legal representative may decide that it is not an appropriate time to discuss the trial or they may decide that the participant would not want to take part, in which case their feelings will be respected and their decision about the participant continuing in the trial will be recorded.

In the event that a personal legal representative cannot be identified, or it is deemed inappropriate to approach the potential personal legal representative, the local research team will work to identify a **professional legal representative** as defined below:

A person independent of the trial, who is the doctor primarily responsible for the medical treatment provided to that adult. Or a person nominated by the relevant healthcare provider.

Informed consent given by a professional representative shall represent the participant's presumed will.

If the participant does regain capacity during the follow-up period, they will be asked to give consent for themselves using the process outlined in **Section 5.1**.

The participant's wishes (consent or refusal) will supersede the personal or professional legal representative consent.

5.3 Consent Arrangements for Participants under the Age of 16

Children who are known or apparently aged <16 years are excluded from participating in the RePHILL Trial. However it is recognised that there may be scenarios where participants under the age of 16 are inadvertently randomised e.g., where they appear older than 16 years and do not have identification with them that confirms their actual age. In this scenario, consent will be sought, after the trial intervention, from a parent or guardian. If the participant has capacity, they will also be asked to provide assent for their continued participation in the trial. A Parent/ Guardian Consent Form and an Assent Form (for participants <16 years) will be provided for this purpose.

5.4 Participants Who Do Not Survive

The most challenging ethical consideration in this trial relates to the inevitable death of some participants. Actively seeking out and informing relatives of trial participation is transparent and avoids potential distress were the family to discover at some future point that their relative had been involved in a research trial. However, informing the family of trial participation in the immediate aftermath of their relative's death will impose an additional emotional burden at a time of great distress. Previous and ongoing emergency care studies have used passive information approaches, placing information in publically accessible locations and in sites likely to be visited by relatives of the deceased (hospitals, GP surgeries, the offices of the Registrars of Births and Deaths). Such information contains brief details of the trial and contact details for those wishing to seek further information about the trial. This allows a relative to make an individual decision as to whether to seek further information as to whether their relative was part of the trial, at a time of their choosing. This is the approach that we will take with the RePHILL trial and a REC approved poster will be placed in appropriate locations of the receiving hospitals.

For those participants that have been randomised, but subsequently die at scene, it will be impossible to obtain any form of consent. The data transferred to the BCTU for these participants will be pseudoanonymised with the trial number (this will be obtained when the RePHILL PHEM Case Report Form (CRF) has been completed) and if available, a partial date of birth. An Exit Form will also be completed for these participants, documenting partial date of death.

Should a participant die *en route* to hospital, the participant's transfer will follow part of the PHEM team's routine process. In these situations, the PHEM team will be responsible for enrolling the patient and completing the RePHILL PHEM CRF and Exit Form. The data that are transferred to BCTU for these participants will also be pseudoanonymised with the trial number and if available, a partial date of birth.

5.5 Participants transferred to non-RePHILL Hospitals

There may be some situations where due to the geographical location of the participant or the severity of their injuries, that the PHEM team will transfer participants to non-RePHILL hospitals. This may mean that participants are taken to non-RePHILL hospital temporarily, to be stabilised before being transferred to a RePHILL hospital. It could also mean that the participant remains at a non-RePHILL hospital permanently for follow-up or end-of-life care (if further treatment is considered futile). As the RePHILL trial only collects a pseudoanonymised, minimal dataset that is part of the participant's standard of care pathway, the RePHILL Trial Office shall engage with non-RePHILL hospitals to request participant data, the details of which are outlined below:

- If the participant is initially stabilised at a non-RePHILL hospital but is then subsequently transferred to a RePHILL hospital, the RePHILL Trial Office will request the following:
    - If consent is obtained at the RePHILL hospital, data collected at the non-RePHILL hospital, which relate to the RePHILL trial, will be requested.
    - If there is no consent in place, a minimal data set will be requested via the ED Admission Form and Exit Form. Both of these forms capture data pertaining to the composite primary outcome measure.
- If a participant is transferred to a non-RePHILL hospital and remains there (for follow-up care, or if they subsequently die), the RePHILL Trial Office will request a minimal data set via the ED Admission Form and Exit Form, as above, these forms capture data pertaining to the composite primary outcome measure.

6 Randomisation and Enrolment Process

6.1 Randomisation Process

Randomisation will be provided by a computer generated programme at the Birmingham Clinical Trials Unit (BCTU). Participants will be randomised at the level of the individual in a 1:1 ratio to either PHBP resuscitation or crystalloid resuscitation. The randomisation procedure will be stratified by IDS to account for variation in trauma care and type of trauma between delivery sites.

6.1.1 Role of Blood Banks

The role of the blood bank in the RePHILL trial will be to maintain a constant supply of randomised trial interventions to the PHEM team. The blood bank will obtain the randomised allocations via a secure online system (available at: https://www.trials.bham.ac.uk/RePHILL) at the BCTU. Unique log-in usernames and passwords will be provided to the blood bank staff supporting the trial. The online system will be available 24 hours a day, 7 days a week apart from short periods of scheduled maintenance. Alternatively, a back-up telephone toll-free allocation service on 0800 953 0274 is available Monday - Friday, 09:00-17:00. This excludes bank holidays and University of Birmingham closed days. If an online connection is not available, telephone allocation and a back-up paper allocation using a simple randomisation list will be used.

Blood banks will be supplied with pre-printed 'treatment box number' labels. A registered user at the blood bank will request a treatment allocation from the BCTU and will receive a treatment box number and treatment arm allocation. The allocated trial intervention will be packed into transport boxes affixing the correct labels. Transport boxes will be issued as a pair, one marked red and one marked yellow per single randomised allocation. The treatment box number should be identical on each coloured box pair carried. The date and time of expiry will also be written on each transport box.

The packed, sealed transport boxes will be dispatched to the PHEM base using an established courier service as required.

6.1.2 Role of PHEM

Upon receiving the box pairs from the blood bank, the PHEM team will need to access the RePHILL online system (https://www.trials.bham.ac.uk/RePHILL) and acknowledge receipt. As part of this process, the PHEM team will need to confirm that the boxes are matched, i.e. that they have the same number on both of them, that they are sealed and the time they were received.

During their shift, the PHEM team should ensure that they are carrying a pair of sealed, red and yellow transport boxes with matched box numbers.

Where possible, unopened transport boxes (with the seal still intact) should be returned to the blood banks prior to expiry, to minimise wastage. PRBC may be returned to stock and re-issued if there have been no temperature excursions.

6.2 Randomisation

The PHEM doctor will assess the potential participant's vital signs on scene and confirm if eligible for entry into the RePHILL trial. If they fulfil the eligibility criteria (as defined in Section 4 of the protocol) then the randomised treatment will be given. **Participants are considered randomised into the trial when the PHEM team open the first transport box containing the allocated trial intervention**. Eligibility will be documented at the Receiving Hospital Site (RHS) and the RePHILL PHEM Case Report Form (CRF) completed at handover in the ED. To receive a Trial Number, a member of the research team will access the online system at the BCTU and enter the information recorded on the PHEM CRF (Section 6.3).

6.3 Enrolment

Delegated site staff can enrol a participant (and obtain a trial number) by accessing the secure online system: https://www.trials.bham.ac.uk/RePHILL. In order to enrol a participant, site staff must have access to the completed 'Eligibility Checklist' and the 'Pre-Hospital Details' sections in the PHEM CRF. All fields must have been completed in order for the participant to be enrolled and a trial number issued.

6.4 Co-enrolment

Due to the emergency nature of this trial, it is highly unlikely that those randomising and enrolling participants to RePHILL will be aware if a participant is already enrolled in a clinical trial. Where a participant is enrolled in RePHILL and is subsequently found to have been participating in a concurrent trial, BCTU will inform the RePHILL CI, who will in turn liaise with the CI for the other trial.

When it is possible to plan in advance, the Trial Management Group (TMG) will consider requests for co-enrolment into other trials in accordance with best practice recommendations [32]. This will ensure careful consideration of participant burden, compatibility of interventions, organisational issues and follow-up. A log of co-enrolled participants will be maintained by BCTU.

6.5 Post Randomisation Exclusions and Withdrawals

Participants who are later found to be ineligible, but who have received the trial intervention will remain in the trial as per protocol and be included in the analysis.

For participants who have withdrawn consent for continuing in the trial, data already collected up until the point of withdrawal will be retained and included in the analysis.

7. Trial Procedures and Assessments

7.1 On-scene

The attending PHEM team (doctor and/or paramedic) will assess eligibility on-scene. Prior to delivery of the intervention, eligible participants will have a capillary blood test taken to measure lactate concentration using a point-of-care lactate device. The capillary blood will be obtained by a finger prick on a test strip, no sample can be retained from this, and therefore no tissue will be stored as a result of this test.

The allocated intervention will then be administered as either:

Crystalloid resuscitation:

- Consisting of up to 4 x 250 mL bags of 0.9% sodium chloride (normal saline). These will be administered as boluses of 250 mL to restore and maintain a systolic blood pressure (SBP) of ≥ 90 mmHg or a palpable radial pulse.

OR

PHBP resuscitation:

- Consisting of up to 2 units of PRBC and 2 units of LyoPlas. These will be administered sequentially.

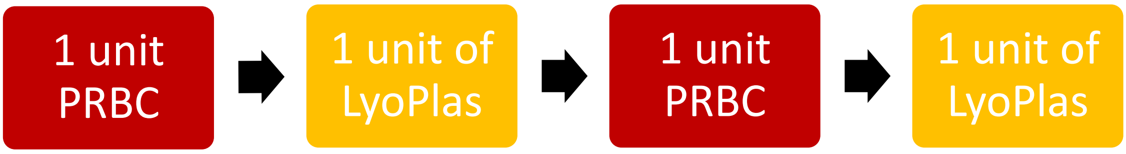

(*The volume of 1 unit PRBC is 270 mL (range: 220 – 340). The volume of reconstituted LyoPlas is 213 mL. Consequently, over the 4 boluses, similar volumes of fluid are administered in each trial arm)*

However if rapid volume transfusion is required (i.e. via more than one line), then the principle of balanced PRBC: LyoPlas transfusion must be adhered to.

In both arms, when possible, all interventions administered (normal saline, PRBC and LyoPlas) should be given through fluid warmers.

7.1.1 Subsequent Boluses

In both arms of the trial: If hypotension is corrected after the administration of a bolus, no further fluid will be administered. If clinically significant hypotension persists, or reoccurs further boluses will be administered until it is corrected. In each case, a maximum of 4 boluses can be administered as part of the trial interventions.

Any additional fluid boluses required to maintain blood pressure after administration of the 4 trial boluses should be given according to standard local practice.

7.1.2 Lactate Concentration

In cases where the participant is still on scene 2 hours after randomisation, a second capillary blood test should be taken to measure lactate concentration using a point-of-care lactate device at 2 hours after randomisation.

7.2 On Arrival at the Receiving Hospital ED

Trial data collected by the PHEM team will be shared with the RHS, in accordance with local policy and recorded on the RePHILL PHEM CRF.

7.2.1 Vital Signs

The following will be measured:

- Heart rate measured in beats per minute (bpm)
- Blood pressure measured in mmHg
- Respiratory rate is the number of breaths (inhalation – exhalation cycles) counted in one minute
- Capillary oxygen saturation (SpO_2) measured by application of a probe to a finger, toe or ear. SpO_2 is the percentage of haemoglobin that is oxygenated

7.2.2 Tissue Oxygenation and Perfusion

Selected sites only

When possible, Near-infra-red spectroscopy (NIRS) will be used to monitor tissue oxygenation and perfusion via a non-invasive adhesive pad attached to the participant's skin.

7.2.3 (Venous) Lactate Concentration

Lactate concentration will be measured on arrival at ED and 2 hours after arrival as part of standard care. **It will also be measured 2 hours after randomisation (if not previously done on scene), for trial purposes**. Where possible, a venous sample should be taken, however if this is not possible then an arterial sample is permitted. Lactate concentration will be measured on a near-patient blood

gas analyser. There is no processing required before analysis. This is drawn as a normal part of trauma care (i.e. is no extra burden for the participant). The blood volume drawn varies between syringes but is typically between 1 mL and 3 mL, to be drawn into a pre-heparinised syringe.

7.2.4 Calculating Lactate Clearance

Lactate clearance[33] is expressed as a percentage per hour (%/h) and is calculated from the measurement of (venous) lactate concentration (with automated analysers that are near-patient) by the PHEM team immediately prior to randomisation (Lac_0) and at 2 hours after randomisation (Lac_h) as:

$$\textbf{Lactate Clearance} = \frac{100 \times (Lac_0 - Lac_h)}{Lac_0 \times \text{Interval}}$$

7.2.5 Blood Samples on Admission

- Routine laboratory testing to include standard care blood tests (including but not limited to, coagulation and transfusion).
- The standard laboratory tests of coagulation are fibrinogen, prothrombin time (PT), activated partial thromboplastin time (APTT), International Normalised Ratio (INR). INR is a ratio of PT to normal, corrected for local processes and reagents, allowing valid comparison between different laboratories.
- Transfusion testing to include ABO and RhD group with assessment for mixed field group and antibody screen.

7.2.6 Blood Sampling for ROTEM®

Selected sites only

Coagulation will be measured viscoelastically by rotational thromboelastometery (ROTEM®):

- For sites using the ROTEM® machine, 4.5 mL of venous blood is to be drawn into a citrated container (BD Vacutainer 367691 or equivalent). The citrated container contains the additive sodium citrate, which inhibits blood clotting. This is a standard tube for blood clotting tests. No pre-processing is required.
- EXTEM and FIBTEM tests will be performed.

7.2.7 Blood Sampling for Platelet Function

Selected sites only

Selected sites will have a platelet function analyser (MultiPlate)[34]. Three MultiPlate tests (using ADP, ASPI and TRAP agonists) will be carried out from a venous blood sample drawn into a 3 evacuated hirudin-coated blood tube (Sarstedt AG & Co. S-Monovette® 04.1944.001 or equivalent).

7.3 During Hospital Admission

The following assessments are to be made during the hospital admission as indicated by the participant's clinical condition, up to withdrawal, acute care discharge, death or day 30, whichever is earlier.

7.3.1 Acute Respiratory Distress Syndrome (ARDS)

This will be assessed at day 7. The Berlin definition of ARDS will be used for assessing participants[34]. Criteria for the diagnosis are shown in Table 1.

Table 1: Criteria for diagnosis of ARDS

**Timing**	Within 1 week of a known clinical insult or new or worsening respiratory symptoms
**Chest imaging**	Bilateral opacities not fully explained by effusions, lobar/lung collapse or nodules
**Origin**	Respiratory failure not fully explained by cardiac failure or fluid overload
**Oxygenation**	PaO_2/FiO_2 ≤40 kPa with either PEEP or CPAP ≥5 cm H_2O (invasive or non-invasive)

7.3.2 Sequential Organ Failure Assessment score (SOFA) score

The extent of a participant's organ dysfunction will be recorded using the SOFA score[31]. The score is based on six components, one each for the following systems:

- Respiratory
- Neurological
- Cardiovascular
- Liver
- Coagulation
- Renal

The scores are assigned as shown in Tables 2 a-f.

The SOFA score will be determined daily for the duration of intensive care stay up to day 30. Scores will be derived from routine clinical and laboratory records.

Appendix page 34

Tables 2a-f: SOFA Score

Respiratory (a)

PaO_2/FiO_2 (kPa)	Score
≥53.3	0
<53.3	1
<40.0	2
<26.7 and mechanically ventilated	3
<13.3 and mechanically ventilated	4

Neurological (b)

Glasgow Coma Scale (GCS)	Score
15	0
13-14	1
10-12	2
6-9	3
<6	4

Cardiovascular (c)

Mean Arterial Pressure or inotrope requirement	Score
MAP ≥70 mmHg	0
MAP <70 mmHg	1
dop ≤5 or dob (any dose)	2
dop >5 OR epi ≤0.1 OR nor ≤0.1	3
dop >15 OR epi>0.1 OR nor >0.1	4
**Key: dop: Dopamine, dob: dobutamine, epi: adrenaline, nor: noradrenaline Doses in µg/kg/min**	

Liver (d)

Bilirubin (µmol/L)	Score
<20	0
20-32	1
33-101	2
102-204	3
>204	4

Coagulation (e)

Platelets×10^3/µl	Score
≥150	0
<150	1
<100	2
<50	3
<20	4

Renal (f)

Creatinine (µmol/L) or urine o/p	Score
≤109	0
110-170	1
171-299	2
300-440 (or <500 mL/day)	3
> 440 (or <200 mL/day)	4

Appendix page 35

7.4 Schedule of Assessments

Refer to Table 3 below:

Table 3: Table of Assessments

	On-scene	ED arrival	2 hours post-randomisation	2 hours post-ED arrival	6 hours post-ED arrival	12 hours post-ED arrival	24 hours post-ED arrival	During hospital stay (up to day 30)
Vital signs	✓	✓		✓	✓	✓	✓	
Confirm eligibility	✓							
[1]Lactate concentration	✓	✓	✓	✓				
Administer allocated treatment	✓							
Legal Representative Consent		✓						
Participant Consent								✓
[2]Blood sampling		✓		✓	✓			✓
ROTEM® (participating sites only)		✓						
Blood sampling (platelet function)		✓						
Record fluids administered	✓	✓		✓	✓	✓	✓	
Record surgical procedures				✓	✓	✓	✓	
SOFA and ARDS								✓
Adverse Events	✓	✓	✓	✓	✓	✓	✓	✓
[3]Date and time of death of non-survivors	✓	✓	✓	✓	✓	✓	✓	✓
[4]Acute care discharge date								✓

1 Capillary lactate concentrations taken on-scene will be measured using a simple point-of-care tester.

2 Standard laboratory tests should include a full blood count and coagulation tests. The normal sampling and laboratory practices of the site shou[illegible] followed.

3 Mortality may extend beyond 30 days as it includes episode mortality

4 Acute care discharge date should be recorded following discharge from acute care. This may extend beyond 30 days.

8. Trial Intervention/Investigational Medicinal Products

8.1 Trial Treatments

8.1.1 PHBP Arm (Lyophilised Plasma LyoPlas N-w (LyoPlas) and PRBC)

LyoPlas is a freeze dried plasma product derived from a single donation and is licenced for use in the same indication as fresh frozen plasma. LyoPlas is licensed for use in Germany as a medicinal product under the Marketing Authorisation Number PEI.H.03075.01.1, and therefore is being classified as an Investigational Medicinal Product (IMP) in the RePHILL trial.

PRBC are a concentrated preparation of red blood cells that is obtained from whole blood by removing the plasma (as by centrifugation).

The PRBC used in RePHILL will be blood group O, RhD negative, Kell negative from NHS Blood and Transplant national stocks supplied by the blood banks that are supporting this trial.

8.1.2 Crystalloid Arm

The crystalloid resuscitation comparator arm will consist of 0.9% sodium chloride (normal saline; a solution of sodium chloride in water). This is classified as the comparator IMP in the RePHILL trial.

8.2 Supply of Trial Stocks and Storage Conditions

8.2.1 Trial Supplies

LyoPlas

The trial stock of LyoPlas will be shipped from the central IMP distribution centre to local receiving site pharmacies. One packaged unit of LyoPlas will comprise:

- 1 glass bottle of 200 mL freeze dried human plasma
- 1 plastic bag containing 200 mL water for injection
- 1 transfer set

PRBC

The PRBC will be from national stocks supplied by the blood banks that are supporting the RePHILL trial.

Normal saline

Normal saline will be from routine NHS stock and does not require any special storage conditions.

8.2.2 Packaging and Labelling of the IMPs

LyoPlas: The central IMP distribution centre will package and label the LyoPlas prior to sending out to local site pharmacies.

Normal saline: Will be provided from local site pharmacies as standard NHS stock and will be labelled by site pharmacies prior to transfer to blood banks.

Both IMPs will be labelled in compliance with the applicable regulatory requirements.

8.2.3 Storage

LyoPlas is stable between 2°C – 25°C and should be maintained within these limits whilst stored in local pharmacies and blood banks..

For the RePHILL trial, once packaged into the trial intervention transport boxes, the LyoPlas should be maintained between 15°C and 25°C to permit ease of preparation and administration.

PRBC is to be maintained at 4°C (± 2°C) in accordance with blood bank standard procedures.

Normal saline should be stored in accordance with the Summary of Product Characteristics (SmPC).

8.3 Administration of Treatment

With respect to the interventions:

- LyoPlas may be administered via either an intravenous (IV) or intraosseous (IO) route after reconstitution in water, in accordance with the manufacturer's instructions.
- PRBC may be administered via either an intravenous (IV) or intraosseous (IO) route, according to standard clinical practice
- Normal saline may be administered via either an intravenous (IV) or intraosseous (IO) route, according to standard clinical practice

Fluid boluses should be administered according to standard practice which will usually require that they are delivered through a fluid warmer.

8.4 Interactions or Contraindications

LyoPlas and PRBC have been prepared with citrate, therefore solutions containing calcium must not be administered concurrently through the same line. Medicinal products should not be added to LyoPlas or PRBC. If an acute transfusion reaction (ATR), including allergic reactions, is suspected following IV/IO infusion of either PRBC or LyoPlas, the transfusion should be stopped immediately. The IV/IO cannula should be retained and the transfusion reaction managed as per standard clinical practice.

9. Pharmacovigilance

9.1 Reporting Requirements

The collection and reporting of Adverse Events (AEs) will be in accordance with the Medicines for Human Use (Clinical Trials) Regulations 2004 and subsequent amendments. The Investigator will assess the seriousness and causality (relatedness) of all applicable AEs experienced by the participant with reference to the reference safety information. This should be documented in the source data with reference to the approved reference safety information (Section 4.8, Undesirable Effects) of the SmPC for Sodium Chloride 0.9% Intravenous Infusion (date: 24th December 2018) and reference safety information (Section 5, Undesirable Effects) of the SmPC for LyoPlas N-w (date: 26th November 2010).

Standard definitions of different types of AEs are listed in **Table 4a** and categorisation of causality shown in **Table 4b**.

Table 4a: Definition of standard terms

Term	Definition
**Adverse Event (AE)**	Any untoward medical occurrence in a participant or clinical trial subject administered a medicinal product and which does not necessarily have a causal relationship with this treatment
**Adverse Reaction (AR)**	Any untoward and unintended response in a subject to an investigational medicinal product which is related (or for which a causal relationship cannot be ruled out) to any dose administered to that subject
**Unexpected adverse reaction**	An adverse reaction the nature and severity of which is not consistent with the information about the medicinal product in question set out:<br>(a) in the case of a product with a marketing authorisation, in the summary of product characteristics for that product;<br>(b) in the case of any other investigational medicinal product, in the investigator's brochure relating to the trial in question.
**Serious adverse event (SAE), serious adverse reaction (SAR) or suspected unexpected serious adverse reaction (SUSAR)**	Any adverse event, adverse reaction or unexpected adverse reaction, respectively, that:<br>• results in death;<br>• is life-threatening;<br>• requires hospitalisation or prolongation of existing hospitalisation;<br>• results in persistent or significant disability or incapacity; or consists of a congenital anomaly or birth defect
**SUSAR**	Suspected Unexpected Serious Adverse Reaction (as defined above)

Table 4b: Categorisation of causality

Category	Definition	Relatedness
**Definitely**	There is clear evidence to suggest a causal relationship, and other possible contributing factors can be ruled out	**Related**
**Probably**	There is evidence to suggest a causal relationship, and the influence of other factors is unlikely	
**Possibly**	There is some evidence to suggest a causal relationship (e.g. the event occurred within a reasonable time after administration of the trial medication). However, the influence of other factors may have contributed to the event (e.g. the participant's clinical condition, other concomitant events)	
**Unlikely**	There is little evidence to suggest there is a causal relationship (e.g. the event did not occur within a reasonable time after administration of the trial medication). There is another reasonable explanation for the event (e.g. the participant's clinical condition, other concomitant treatments)	**Unrelated**
**Not related**	There is no evidence of any causal relationship	

In the RePHILL trial, the LyoPlas and normal saline are categorised as the IMPs and the pharmacovigilance reporting requirements that will be followed are described in this section of the protocol.

As this is a trial using an intervention that also includes a blood component (PRBC), the statutory arrangements for haemovigilance should also be followed (refer to **Section 9.7**).

AEs will be recorded in the medical records as per standard clinical practice. Most (S)AE/ARs that occur in this trial, whether they are serious or not, will be 'expected' treatment-related consequences of the trial intervention or trauma related.

9.2 (Serious) Adverse Events

RePHILL trial participants are likely to have significant co-morbidities and therefore the frequency of AEs is likely to be high. Most of the AEs occurring in RePHILL, whether serious or not, will therefore be anticipated in the sense that they are recognised and accepted complications/consequences of major trauma.

Investigators will report AEs that meet the definition of an SAE, other than the SAEs listed in **Section 9.2.1**

9.2.1 Events that do not Require Reporting on a SAE Form

The following are regarded as expected SAEs (i.e. are recognised complications/consequences of major trauma) for the purpose of this trial and should not be reported on an SAE Form. These events

should be reported on the appropriate trial CRF(s) instead and will not be subject to expedited reporting.

Event	CRF
Death (from trauma)	Exit Form
Organ failure (single organ)	Daily Assessments
Multi organ dysfunction syndrome	Daily Assessments
Systemic inflammatory response syndrome	Daily Assessments
Acute respiratory distress syndrome	Daily Assessments – Day 7
Infection (any anatomical site)	Daily Assessments
Venous thromboembolism (deep venous thrombosis or pulmonary embolism)	Daily Assessments – Day 7
Transfusion reactions occurring after ED arrival	24 Hour FU and Daily Assessments

SAEs that are related to a pre-existing condition are not required to be reported.

9.2.2 Monitoring Participants Pregnancies for Potential SAEs

Known pregnancy at the time of enrolment is an exclusion criterion for the RePHILL trial, however, should a participant later be found to have been pregnant at the time of trauma and received the trial intervention, the outcome of the pregnancy (spontaneous miscarriage, elective termination, normal birth or congenital abnormality) must be followed-up and documented, even if the participant withdrew consent from follow-up within the trial. Initial notification of pregnancy will be done via a SAE form and the outcome of the pregnancy will be recorded on the Pregnancy Notification Form. These will be reported to the RePHILL Trial Office.

9.2.3 Reporting Period

Details of all SAEs (except those listed as excluded) will be documented and reported from the date of commencement of protocol defined treatment.

9.3 Reporting Procedure – Site

9.3.1 Serious Adverse Events

Receiving hospitals should report SAE's which are NOT listed as recognised complications of major trauma (as defined in section 9.2.1), to the RePHILL Trial Office on a SAE Form as soon as possible and no later than 24 hours after becoming aware of the event.

Complete SAE Forms should be faxed to the RePHILL Trial Office on:

0121 415 9135 or call 0121 414 7943 or 0121 415 8445

The research team at site will be required to respond to any related queries raised by the RePHILL Trial Office as soon as possible.

Site Investigators should also notify their own institutions of any SAEs in accordance with their institutional policies.

For SAE Forms completed by someone other than the Investigator, the Investigator will be required to countersign the original SAE Form to confirm agreement with the categorisation of seriousness and causality assessments. The SAE Form should then be returned to the RePHILL Trial Office and a copy retained at site.

9.3.2 Provision of Follow-up Information

Participants should be followed up until resolution or stabilisation of the event. Follow-up information should be provided as soon as available.

9.4 Reporting Procedure – Trial Office

On receipt of the SAE form, the RePHILL Trial Office will allocate each SAE a unique reference number which will be forwarded to the receiving hospital as proof of receipt. The SAE reference number should be quoted on all correspondence and follow-up reports regarding the SAE.

On receipt of an SAE Form, seriousness and causality will be reviewed independently by the Chief Investigator (CI; or nominated delegate). A SAE judged to have a reasonable causal relationship with the trial intervention will be regarded as a Serious Adverse Reaction (SAR). The causality assessment given by the PI will not be downgraded by the CI *"or delegate(s)"*. If the CI *"or delegate(s)"* disagrees with the PI's causality assessment, the opinion of both parties will be documented, and where the event requires further reporting, the opinion will be provided with the report.

The CI (or nominated individual) will also assess all SARs for expectedness. If the event meets the definition of a SAR that is unexpected (i.e. is not defined in the approved version of the Reference Safety Information) it will be classified as a Suspected Unexpected Serious Adverse Reaction (SUSAR).

9.5 Reporting to the Competent Authority and Research Ethics Committee

9.5.1 Suspected Unexpected Serious Adverse Reactions

The RePHILL Trial Office will report a minimal data set of all individual events categorised as a fatal or life threatening SUSAR to the Medicines and Healthcare products Regulatory Agency (MHRA) and REC within 7 days. Detailed follow-up information will be provided within an additional 8 days.

All other events categorised as non-life threatening SUSARs will be reported within 15 days.

A copy will be sent to the Trial Sponsor at the time of sending the SUSAR report.

9.5.2 Serious Adverse Reactions

The RePHILL Trial Office will report details of all SAEs and SARs (including SUSARs) to the MHRA and REC annually from the date of the Clinical Trial Authorisation, in the form of a Development Safety Update Report (DSUR).

A copy will also be sent to the Sponsor at the time of sending out the DSUR.

9.5.3 Other Safety Issues Identified during the Course of the Trial

The MHRA, REC and Sponsor will be notified immediately if a significant safety issue is identified during the course of the trial.

9.6 Investigators

Details of all SUSARs and any other safety issue(s) which arise during the course of the trial will be reported to Principal Investigators (PI). A copy of any such correspondence should be filed in the Investigator Site File (ISF).

9.7 Haemovigilance

Staff at IDS will be responsible for reporting all transfusion-related adverse events via Serious Hazards Of Transfusion and Serious Adverse Blood Reactions and Events (SHOT/SABRE) according to standard procedures, as required under the regulations of the EU Blood Safety Directive[35, 36]. Similarly, the receiving hospital staff are also responsible for reporting all transfusion-related adverse events, including acute transfusion reactions (<24 hr) and delayed transfusion reactions (>24 hr), to SHOT/SABRE according to standard procedures.

Each individual blood bank issuing blood will have their own their local policies and procedures for the response to a possible transfusion event and should ensure full compliance with their own licence and MHRA. Where the receiving hospital blood bank is different from the issuing hospital blood bank, then both parties should co-ordinate to ensure traceability and reporting.

The hospital blood transfusion laboratory that provided the PRBC (coordinating blood bank) must be informed immediately of all adverse events and reactions. Advice on clinical management and investigation of serious adverse reactions can be obtained from the hospital consultant responsible for blood transfusion at the coordinating blood bank.

9.8 Developmental Safety Update Reports

The RePHILL Trial Office will provide the MHRA with DSURs. The reports will be submitted within 60 days of the Development International Birth Date (DIBD) of the trial each year until the trial is declared ended.

9.9 Annual Progress Reports

An Annual Progress Report will be submitted to the REC within 30 days of the anniversary date on which the favourable opinion was given and annually until the trial is declared ended. A copy will also be sent to the Sponsor at the time of sending out the DSUR.

9.10 Reporting Urgent Safety Measures

If any urgent safety measures are taken, the CI/BCTU shall immediately, and in any event no later than 3 days from the date the measures are taken, give written notice to the REC and MHRA of the measures taken and the circumstances giving rise to those measures.

9.11 Notification of Serious Breaches of Good Clinical Practice and/or the Protocol

In accordance with Regulation 29A of the Medicines for Human Use (Clinical Trials) Regulations 2004 and its amendments, the Sponsor of the trial is responsible for notifying the licensing authority in writing of any serious breach of the conditions and principles of Good Clinical Practice (GCP) in connection with that trial or the protocol relating to that trial, within 7 days of becoming aware of that breach.

For the purposes of this regulation, a “serious breach” is a breach which is likely to effect to a significant degree the safety or physical or mental integrity of the subjects of the trial; or the scientific value of the trial. Sites are therefore requested to notify the RePHILL Trial office of any suspected trial-related serious breach of GCP and/or the trial protocol. Where the RePHILL Trial office is investigating whether or not a serious breach has occurred, sites are also requested to cooperate with the Trial Office in providing sufficient information to report the breach to the MHRA where required and in undertaking any corrective and/or preventive action. Sites may be suspended from further recruitment in the event of serious and persistent non-compliance with the protocol and/or GCP, and/or poor recruitment.

The BCTU on behalf of the Sponsor shall notify the MHRA and REC in writing of serious breaches.

10. Data Management and Quality Assurance

10.1 Confidentiality

All data will be handled in accordance with the current UK recommended data protection regulations.

10.2 Data Collection

During the hospital admission, up to withdrawal, discharge from acute care, death or day 30 (whichever is earlier), where possible, outcome data will be extracted from participant's clinical notes and laboratory reports, to complete the RePHILL trial CRFs (**Table 5**).

Table 5: RePHILL Trial CRFs

Form Name
PHEM CRF
ED Admission CRF
2, 6, 12 and 24 hour Follow-Up CRFs
ROTEM CRF
Impedance Aggregometry and NIRS CRF
Daily Assessments
Medical History CRF
Exit Form
Serious Adverse Event Form
Pregnancy Notification Form

It is the responsibility of the Investigator to ensure the accuracy of all data entered in the CRFs. The RePHILL Trial Signature and Delegation Log will identify all those personnel with responsibilities for data collection. The Trial Office must be informed immediately of any change in the site research team.

Prior to commencing recruitment, all sites will undergo a process of initiation and will have completed GCP training. Key members of the site research team will be required to attend either a meeting or a teleconference covering aspects of the trial design, protocol procedures, AE, collection and reporting of data and record keeping. Sites will be provided with an ISF containing essential documentation, instructions, and other documentation required for the conduct of the trial.

The CRFs will comprise, but will not necessarily be limited to those listed in Table 5.

If paper CRFs are being completed, they must be signed and dated and returned to the RePHILL Trial Office by the PI or an authorised member of the site research team (as delegated on the RePHILL Trial Signature & Delegation Log) within the timeframe listed in the table above. Entries on paper CRFs should be made in ballpoint pen, in black ink, and must be legible. Any errors should

be crossed out with a single stroke, the correction inserted and the change initialled and dated. If it is not obvious why a change has been made, an explanation should be written next to the change. Data reported on each CRF should be consistent with the related source data or the discrepancies should be explained. If information is not known, this must be clearly indicated on the CRF. All sections should be completed; all missing and ambiguous data will be queried. In all cases it remains the responsibility of the PI to ensure that the CRF has been completed correctly and that the data are accurate. Paper CRFs received will be entered onto the trial database by a trained member of the BCTU trial team.

If remote electronic data entry is being undertaken then CRFs should be entered online at: https://www.trials.bham.ac.uk/RePHILL. Authorised staff at IDS and RHS will require an individual secure login username and password to access this online data entry system. As above, data reported should be consistent with the related source data and all missing and erroneous data will be queried.

CRF version numbers may be updated by the RePHILL Trial Office, as appropriate, throughout the duration of the trial. Whilst this will not constitute a protocol amendment, new versions of the CRFs must be implemented by participating sites immediately on receipt.

It will be the responsibility of the PI to ensure the accuracy of all data entered in the CRFs. The RePHILL Trial Signature and Delegation Log will identify all those personnel with responsibilities for data collection.

Access to data, including the final trial dataset will be limited to the Research Team.

The investigator(s)/ institution(s) will permit trial-related monitoring, audits REC review and regulatory inspection(s), providing direct access to source data/ documents. Trial participants are informed of this during the informed consent discussion and will consent to provide access their medical notes.

11. Statistical Considerations

11.1 Sample Size

Although no definitive data exists on this composite outcome, the observational studies suggest potentially dramatic reductions in mortality from civilian pre-hospital PRBC[21] and military pre-hospital PRBC with thawed plasma[17]. Following extensive consultation with experts in pre-hospital trauma resuscitation, it is considered that an absolute reduction of 10% in the proportion of participants experiencing one of the component primary outcomes is clinically meaningful for the participants and is an appropriate effect size upon which to base the power calculation.

To detect an absolute difference of 10% between groups in the proportion of participants experiencing either episode mortality or lactate clearance <20%/h in the two hours post-randomisation (i.e. from 20% in the standard care group to 10% in the group receiving PHBP) using the method of difference between proportions (2-sided Fisher's Exact Test) with 80% power, and a type 1 error rate of 5% (i.e. α=0.05), requires 219 participants per group to be randomised, 438 participants in total. Assuming and adjusting for a 10% loss to follow-up rate, 490 participants will need to be recruited.

The interim analysis for the DMEC meeting in May 2018 reported the results on the 192 participants recruited by 20th April 2018. A pooled event rate of 65% experiencing either episode mortality or lactate clearance <20%/h in the two hours post-randomisation was observed in these participants. This observed rate does not correspond with the pooled event rate of 15% assumed in the original sample size calculations. On the DMECs recommendations, this issue was discussed with the TSC in October 2018, and it was agreed that the power calculations will be framed in terms of relative risk rather than absolute risk but the original target sample size of 490 will not be changed.

Assuming the pooled event rate remains at 65% and allowing for a 10% loss to follow-up rate, 490 participants will provide 80% power to detect a relative risk ratio of 0.82 (i.e. from 71.7% in the standard care group to 58.3% in the group receiving PHBP) using the method of difference between proportions (2-sided Fisher's Exact Test), and a type 1 error rate of 5% (i.e. α=0.05). This estimated relative risk ratio is consistent with the relative risk ratios of 1·54[38] and 0.70[39] reported in two recent pre-hospital RCTs using plasma in one of the treatment arms.

11.2 Analysis of Outcome Measures

A separate Statistical Analysis Plan (SAP) will be produced and will provide a more comprehensive description of the planned statistical analyses. A brief outline of these analyses is given below.

The primary comparison groups will be composed of those resuscitated with PHBP versus those resuscitated with normal saline. All analyses will be based on the intention to treat principle, i.e. all participants will be analysed in the groups to which they were allocated irrespective of compliance with the randomised treatment allocation or other protocol violation. For all major outcome measures, summary statistics and differences between groups (e.g. mean differences, relative risks, hazard ratios) will be presented, with 95% confidence intervals and p-values from two-sided tests also given. The data will be assessed for normality and appropriate data transformations or non-parametric tests will be used if necessary. Outcomes will be adjusted for the minimisation variable, IDS, where possible. A p-value of <0.05 will be considered statistically significant, and no adjustment for multiple comparisons will be made.

11.2.1 Primary Outcome Analysis

The primary outcome is a composite measure of episode mortality[VII] and early lactate clearance (see section 7.2.4 for formula for calculating lactate clearance) and is measured as a binary outcome. Participants clearing less than 20% per hour of their lactate between randomisation and 2 hours after randomisation or dying will be defined as experiencing the primary outcome. A log-binomial regression model, adjusting for IDS, will be used to calculate the relative risk and 95% confidence interval. As this is a composite endpoint, it will also be reported in accordance with the recommendations of Ferreira-González et al[37].

11.2.2 Secondary Outcome Analysis

Dichotomous data (e.g. development of ARDS, mortality at specified time-points) will be analysed in the same way as the primary outcome. Survival data (e.g. mortality) will be analysed using the log-rank test with a Cox Proportional Hazard model used to calculate hazard ratios, if the assumptions of proportionality are met. Continuous outcomes (e.g. pre-hospital fluid volume, vital signs) will be analysed at specified time-points using linear regression models, with mean differences and 95% confidence intervals reported.

11.2.3 Subgroup Analyses

Eleven a priori subgroup analyses are planned with respect to both the primary and secondary outcome measures. The subgroups will be IDS, mode of transport (air .vs. ground), initial lactate concentration (≤ 2.2 mmol/L .vs. >2.2 mmol/L), time to ED from injury (≤ 1 hour .vs. >1 hour), mode of injury (blunt, penetrating, crush), volume of pre-hospital fluid given (total intervention 4 units) vs. those not receiving the total intervention), age (<50 years, 50-70 years, >70 years), head injury (positive vs. negative), compressible haemorrhage (compressible haemorrhage vs. non-compressible haemorrhage), pre-morbid drug history (anticoagulant or antiplatelet medication vs. no anticoagulant or antiplatelet medication) and age of blood products (<8 days vs. ≥ 8 days). Tests for statistical heterogeneity (e.g. by including the treatment group by subgroup interaction parameter in the regression model) will be performed prior to any examination of effect estimates within subgroups. The results of subgroups will be treated with caution and will be used for the purposes of hypothesis generation only.

[VII] Episode mortality refers to mortality between time of injury/ recruitment and discharge from the primary receiving facility to non-acute care, i.e. discharge home or to long-term care, to rehabilitation or repatriation to a hospital closer to their normal residence.

11.2.4 Missing Data and Sensitivity Analyses

Every attempt will be made to collect full follow-up data on all study participants, it is thus anticipated that missing data will be minimal. Participants with missing primary outcome data will not be included in the primary analysis in the first instance. This presents a risk of bias, and sensitivity analyses will be undertaken to assess the possible impact of the risk. In brief, missing responses will be simulated using a Markov chain Monte Carlo method (MCMC) to generate multiple datasets. Analysis will be then be performed on each set with the results combined using Rubin's rule to obtain a single set of results (treatment effect estimate and confidence intervals). Any sensitivity analyses will not, irrespective of their differences, supplant the planned primary analyses. Full details will be included in the SAP.

11.3 Planned Interim Analyses

Interim analyses of major outcome measures and safety data will be conducted and provided in strict confidence to the independent DMEC (see section 17.3). Details of the agreed plan will be written in the SAP.

11.4 Planned Final Analyses

The final analysis for the study will occur once all participants have completed the trial as per the end of trial definition and corresponding outcome data has been entered onto the study database and validated as being ready for analysis.

12. End of Trial

For participants, the main trial data collection ends at withdrawal, acute care discharge, death, or at 30 days follow-up, whichever occurs first. Apart from episode mortality data which will be collected up to discharge from an acute care setting, which may be >30 days.

The end of trial will be six months after the date of last data capture (to include resolution of missing data and data queries). The RePHILL Trial Office will notify the MHRA and REC that the trial has ended within 90 days of the end of trial. Where the trial has terminated early, the RePHILL Trial Office will inform the MHRA and REC within 15 days of the end of trial. The RePHILL Trial Office will provide them with a summary of the clinical trial report within 12 months of the end of trial.

A copy of the end of trial notification as well as the summary report will also be sent to the Sponsor at the time of sending these to the MHRA and REC.

13. Archiving

All essential documents within the Trial Master File will be archived for up to 25 years after completion of the trial. Electronic data sets will be stored indefinitely.

It is the responsibility of the Principal Investigators at sites to ensure all essential trial documentation and source documents (e.g. signed Informed Consent Forms, Investigator Site Files, Pharmacy Files, participants' hospital notes, copies of CRFs etc.) at their site are securely retained for at least 25 years.

14. Direct Access to Source Data

The investigator(s)/institution(s) will permit trial-related monitoring, quality checks, audits, REC review, and regulatory inspection(s), providing direct access to source data/documents. The Principal Investigator will comply with these visits and any required follow up. Sites are also requested to notify the RePHILL Trial Office of any MHRA inspections.

Trial participants who regain capacity will be informed of this during the informed consent discussion and will consent to provide access to their clinical notes. Personal or legal representatives will be informed of this during the informed consent discussion where consent is being sought due to lack of participant capacity and will also consent to provide access to the participant's clinical notes for these purposes.

15. Ethics and Regulatory Requirements

The trial will be performed in accordance with the recommendations guiding physicians in biomedical research involving human subjects, adopted by the 18th World Medical Association General Assembly, Helsinki, Finland, June 1964, amended at the 48th World Medical Association General Assembly, Somerset West, Republic of South Africa, October 1996 (website: http://www.wma.net/en/30publications/10policies/b3/index.html).

The trial will be conducted in accordance with the UK Policy Framework for Health and Social Care Research, the applicable UK Statutory Instruments, (which include the Medicines for Human Use Clinical Trials 2004 and subsequent amendments and the current UK recommended data protection regulations including the Data Protection Act 2018.

This trial will be carried out under a Clinical Trial Authorisation (CTA) in accordance with the Medicines for Human Use Clinical Trials regulations. The protocol will be submitted to and approved by the REC prior to circulation.

Before any participants are enrolled into the trial, the Principal Investigator at each site is required to obtain local R&D approval. Sites will not be permitted to enrol participants until written confirmation of R&D approval is received by the Principal Investigator.

It is the responsibility of the Principal Investigator to ensure that all subsequent amendments gain the necessary local approval. This does not affect the individual clinicians' responsibility to take immediate action if thought necessary to protect the health and interest of individual participants.

16. Monitoring Requirement

Monitoring of RePHILL will ensure compliance with GCP. A risk proportionate approach to the initiation, management and monitoring of RePHILL will be adopted and outlined in the trial-specific risk assessment.

The RePHILL Trial Office will be in regular contact with the site research team to check on progress and address any queries that they may have. The Trial Office will check incoming CRFs for compliance with the protocol, data consistency, missing data and timing. Sites will be asked for missing data or clarification of inconsistencies or discrepancies. Sites will be requested to send in copies of signed ICFs and other documentation for in-house review for all participants giving explicit consent.

Additional on-site monitoring visits may be triggered, for example poor CRF return, poor data quality, excessive number of deviations. This will be detailed in the monitoring plan. If a monitoring visit is required, the RePHILL Trial Office will contact the site to arrange a date for the proposed visit and will provide the site with written confirmation. Investigators will allow the RePHILL trial staff access to source documents as requested.

17. Oversight Committees

17.1 Trial Management Group

The TMG will comprise the CI, other lead investigators (clinical and non-clinical) and members of the BCTU. The TMG will be responsible for the day-to-day running and management of RePHILL. It will convene at regular intervals.

17.2 Trial Steering Committee (TSC)

The role of the TSC is to provide the overall supervision of the trial. The TSC will monitor trial progress and conduct and advise on scientific credibility. The TSC will consider and act, as appropriate, upon the recommendations of the DMEC. Further details of the remit and role of the TSC are available in the TSC Charter.

17.3 DMEC

An independent DMEC will be established to oversee the safety of participants in the trial. The DMEC will meet prior to the trial opening to enrolment and again once the first 25 participants have been entered into the study or at the end of the 6 month internal pilot trial part, whichever occurs first, to assess the safety data, and advise on continuation to the main phase III trial (see Section 3.2 for the Internal Pilot Stopping Rules). Since this is an internal pilot trial, and this safety data will be included

in the main analysis of the RePHILL trial, this data will remain confidential, except to members of the DMEC and the trial statistician(s) performing the analysis.

During the main phase III trial, the DMEC will meet at least annually, or as per a timetable agreed by the DMEC prior to trial commencement. Data analyses will be supplied in confidence to the DMEC, which will be asked to give advice on whether the accumulated data from the trial, together with the results from other relevant research, justifies the continuing recruitment of further participants. The DMEC will operate in accordance with the trial specific charter.

If one treatment really is substantially better or worse than the other with respect to the primary outcome, then this may become apparent before the target recruitment has been reached. Alternatively, new evidence might emerge from other sources that any one treatment is definitely more, or less, effective than the other. To protect against this, during the main period of recruitment to the trial, interim analyses of the primary outcome and adverse events will be supplied, in strict confidence, to the independent DMEC, along with updates on results of other related studies, and any other analyses that the DMEC may request. The DMEC will advise the chair of the TSC if, in their view, any of the randomised comparisons in the trial have provided both (a) “proof beyond reasonable doubt”[VIII] that for all, or for some, types of participants one particular treatment is definitely indicated or definitely contraindicated in terms of a net difference in the major endpoints, and (b) evidence that might reasonably be expected to influence the participant management of many clinicians who are already aware of the other main trial results. The TSC can then decide whether to close or modify any part of the trial. Unless this happens, however, the TMG, TSC, the investigators and all of the central administrative staff (except the statisticians who supply the confidential analyses) will remain unaware of the interim results.

18. Finance

The National Institute for Health Research (NIHR) Efficacy & Mechanism Evaluation Programme is funding this trial (project number 14/152/14).

19. Indemnity

This is a clinician-initiated study. There are no special arrangements to provide compensation for non-negligent harm to participants. The “Clinical Trial Compensation Guidelines” published by the ABPI will not apply.

[VIII] Appropriate criteria of proof beyond reasonable doubt cannot be specified precisely, but a difference of at least $p<0.001$ (similar to a Haybittle-Peto stopping boundary) in an interim analysis of a major endpoint may be needed to justify halting, or modifying, the study prematurely. If this criterion were to be adopted, it would have the practical advantage that the exact number of interim analyses would be of little importance, so no fixed schedule is proposed.

Participants who sustain injury and wish to make a claim for compensation should do so in writing in the first instance to the organisation where they were treated.

Non-NHS organisations taking part in the study and NHS organisations who are not members of their appropriate national clinical negligence scheme (for example CNST in England, CNORIS in Scotland) must take out adequate insurance, or provide other indemnity satisfactory to the sponsor, to cover their potential liabilities against claims for negligence, and must be able to provide evidence of the cover if requested by the sponsor. The University of Birmingham has in force, a public liability policy and/ or clinical trials policy which provides cover for claims of 'negligent harm' and the activities here are included in that coverage.

Appendix page 53

20. Dissemination and publication

Regular newsletters will keep collaborators informed of trial progress, and regular meetings will be held to report progress of the trial and to address any problems encountered in the conduct of the trial.

The CI will coordinate dissemination of data from RePHILL. All publications and presentations, including abstracts, relating to the main trial will be authorised by the RePHILL TMG. The results of the analysis will be published in the name of the RePHILL Trial Investigators in a peer reviewed journal (provided that this does not conflict with the journal's policy). All contributors to the trial will be listed, with their contribution identified. Trial participants will be sent a summary of the final results of the trial, which will contain a reference to the full paper. All applications from groups wanting to use RePHILL data to undertake original analyses will be submitted to the TMG for consideration before release. To safeguard the scientific integrity of RePHILL, trial data will not be presented in public before the main results are published without the prior consent of the TMG.

References

1. Karam, O., et al., *Plasma transfusion strategies for critically ill patients*, in *Cochrane Database of Systematic Reviews*. 2013. p. CD010654.

2. Curry, N., et al., *The acute management of trauma hemorrhage: A systematic review of randomized controlled trials.* Critical Care, 2011. **15**(2): p. R92.

3. Holcomb, J.B., et al., *Transfusion of plasma, platelets, and red blood cells in a 1:1:1 vs a 1:1:2 ratio and mortality in patients with severe trauma: the PROPPR randomized clinical trial.* JAMA, 2015. **313**(5): p. 471-82.

4. Bickell, W.H., et al., *Immediate versus delayed fluid resuscitation for hypotensive patients with penetrating torso injuries.* New England Journal of Medicine, 1994. **331**(17): p. 1105-9.

5. Turner, J., et al., *A randomised controlled trial of prehospital intravenous fluid replacement therapy in serious trauma.* Health Technology Assessment, 2000. **4**(31): p. 1-57.

6. Calderbank, P., et al., *Doctor on board? What is the optimal skill-mix in military pre-hospital care?* Emergency Medicine Journal, 2011. **28**(10): p. 882-3.

7. London's Air Ambulance. *UK's first air ambulance to carry blood on board*. 2012 06.03.12]; Available from: http://www.londonsairambulance.co.uk/news/305-news-uks-first-air-ambulance-to-carry-blood-on-board.

8. Holcomb, J.B., et al., *Prehospital Transfusion of Plasma and Red Blood Cells in Trauma Patients.* Prehosp Emerg Care, 2015. **19**(1): p. 1-9.

9. Ischerwood, S., et al., *Pre-hospital blood transfusions on a UK Air Ambulance service: the first ten months*. 2014.

10. Weaver, A.E., et al., *The introduction of on-scene blood transfusion in a civilian physician-led pre-hospital trauma service.* Scandinavian Journal of Trauma, Resuscitation and Emergency Medicine, 2013. **21**(Suppl 1): p. S27

11. Hauser, C.J., et al., *Results of the CONTROL trial: efficacy and safety of recombinant activated Factor VII in the management of refractory traumatic hemorrhage.* Journal of Trauma - Injury, Infection and Critical Care, 2010. **69**(3): p. 489-500.

12. Roberts, I., B. K, and K. Dickinson, *Medical anti-shock trousers (pneumatic anti-shock garments) for circulatory support in patients with trauma*, in *Cochrane Database Syst Rev*. 1999. p. CD001856.

13. *Prehospital Air Medical Plasma Trial (PAMPer)*. 13 Sep 2014]; Available from: http://clinicaltrials.gov/show/NCT01818427.

14. Murad, M.H., et al., *The effect of plasma transfusion on morbidity and mortality: a systematic review and meta-analysis.* Transfusion, 2010. **50**(6): p. 1370-83.

15. Bhangu, A., et al., *Meta-analysis of plasma to red blood cell ratios and mortality in massive blood transfusions for trauma.* Injury, 2013. **44**(12): p. 1693-9.

16. Morrison, J.J., et al., *En-route care capability from point of injury impacts mortality after severe wartime injury.* Annals of Surgery, 2013. **257**(2): p. 330-4.

17. O'Reilly, D.J., et al., *Prehospital blood transfusion in the en route management of severe combat trauma: A matched cohort study.* Journal of Trauma and Acute Care Surgery, 2014. **77**(3 Suppl 2): p. S114-20.

18. Jansen, J.O., et al., *Changes in blood transfusion practices in the UK role 3 medical treatment facility in Afghanistan, 2008-2011.* Transfusion Medicine, 2014. **24**(3): p. 154-61.

19. Sumida, M.P., et al., *Prehospital blood transfusion versus crystalloid alone in the air medical transport of trauma patients.* Air Medical Journal, 2000. **19**(4): p. 140-3.

20. Smith, I.M., et al., *Pre-hospital blood product resuscitation: benefits unclear*, in *Military Health System Research Symposium*. 2014: Fort Lauderdale, Florida, USA.

21. Brown, J.B., et al., *Pretrauma center red blood cell transfusion is associated with reduced mortality and coagulopathy in severely injured patients with blunt trauma.* Annals of Surgery, 2015. **261**(5): p. 997-1005.

22. Smith, I.M., et al., *Pre-hospital blood product resuscitation for trauma: a systematic review*, in *Military Health System Research Symposium*. 2015: 17-20 Aug 2015, Fort Lauderdale, Florida, USA.

23. Glassberg, E., et al., *Freeze-dried plasma at the point of injury: from concept to doctrine.* Shock, 2013. **40**(6): p. 444-50.

24. Sailliol, A., et al., *The evolving role of lyophilized plasma in remote damage control resuscitation in the French Armed Forces Health Service.* Transfusion, 2013. **53 Suppl 1**: p. 65S-71S.

25. Martinaud, C., et al., *Use of freeze-dried plasma in French intensive care unit in Afghanistan.* Journal of Trauma-Injury Infection & Critical Care, 2011. **71**(6): p. 1761-4.

26. The National CJD Research & Surveillance Unit. *Varient Creutzfeldt-Jakob Diesase: Current Data (June 2014)*. 2014 22 Dec 2014]; Available from: http://www.cjd.ed.ac.uk/documents/worldfigs.pdf.

27. Advisory Committee on the Safety of Blood, Tissues and Organs. *Importation of plasma as a vCJD risk reduction measure: reconsideration of "acceptable" source countries*. 2013 22 Dec 2014]; Available from: https://www.gov.uk/government/uploads/system/uploads/attachment_data/file/192586/Plasma_sourcing_paper_final_version.pdf.

28. Hasler, R.M., et al., *Systolic blood pressure below 110mmHg is associated with increased mortality in blunt major trauma patients: Multicentre cohort study.* Resuscitation, 2011. **82**(9): p. 1202-1207.

29. Hasler, R.M., et al., *Systolic blood pressure below 110 mmHg is associated with increased mortality in penetrating major trauma patients: Multicentre cohort study.* Resuscitation, 2012. **83**(4): p. 476-81.

30. Chase, J.G., et al., *Organ failure and tight glycemic control in the SPRINT study.* Critical Care (London, England), 2010. **14**(4): p. R154.

31. Vincent, J.L., et al., *The SOFA (Sepsis-related Organ Failure Assessment) score to describe organ dysfunction/failure. On behalf of the Working Group on Sepsis-Related Problems of the European Society of Intensive Care Medicine.* Intensive Care Medicine, 1996. **22**(7): p. 707-10.

32. Krige, A., et al., *Co-Enrolment to Intensive Care Studies – A UK Perspective.* Journal of the Intensive Care Society, 2013. **14**(2): p. 103-106.

33. Regnier, M.-A., et al., *Prognostic significance of blood lactate and lactate clearance in trauma patients.* Anesthesiology, 2012. **117**(6): p. 1276-88.

34. The ARDS Definition Task Force, et al., *Acute respiratory distress syndrome: the Berlin Definition.* JAMA, 2012. **307**(23): p. 2526-33.

35. Kosmirak, L., *NHSBT Portfolio of Blood Components and Guidance for their Clinical Use.*, in *Specification SPN223.* 2014, NHS Blood & Transplant: Version 6.2. Watford.

36. *The Blood Safety and Quality Regulations*, in *SI 2005/50*. 2005: London.

Appendix page 57

37. Ferreira-Gonzalez, I., et al., *Methodologic discussions for using and interpreting composite endpoints are limited, but still identify major concerns.* Journal of Clinical Epidemiology, 2007. **60**(7): p. 651-657.

38. Moore HB, Moore EE, Chapman MP, et al., *Plasma-first resuscitation to treat haemorrhagic shock during emergency ground transportation in an urban area: a randomised trial.* Lancet, 2018. **392**: p. 283-291

39. Sperry JL, Guyette FX, Brown JB, et al., *Prehospital Plasma during Air Medical Transport in Trauma Patients at Risk for Hemorrhagic Shock.* New England Journal of Medicine, 2018.**379**: p. 315-326.

Surgical Reconstruction and Microbiology Research Centre

NHS National Institute for Health Research

UNIVERSITY OF BIRMINGHAM | BCTU Birmingham Clinical Trials Unit

A Multi-Centre Randomised Controlled Trial of Pre-Hospital Blood Product Administration versus Standard Care for Traumatic Haemorrhage: The RePHILL Trial

Trial Registration: ISRCTN 62326938

Statistical Analysis Plan

SAP Version Number	Protocol Version Number
1.0	3.0

Name of Author:	Jon Bishop	Role:	Trial Statistician	Affiliation:	BCTU<br>University of Birmingham
Signature of Author:		Date:			
Name of Chief Investigator:	Prof Gavin Perkins	Role:	Chief Investigator	Affiliation:	Warwick Medical School and University Hospitals Birmingham NHS Foundation Trust
Signature of Chief Investigator:	[signature]	Date:	28-JUN-2021		
**This Statistical Analysis Plan has been approved by:**					
Name of Approver:	Natalie Rowland	Role:	Senior Statistician	Affiliation:	BCTU<br>University of Birmingham
Signature of Approver:		Date:			

SAP version number	SAP section number	Description of and reason for change	Timing of change with respect to interim/final analysis/databas e lock	Blind Reviewer	
				Name:	
				Signature:	
				Date:	
				Name:	
				Signature:	
				Date:	
				Name:	
				Signature:	
				Date:	

Statistical Analysis Plan (SAP) Amendments

Abbreviations & Definitions	
**Abbreviation / Acronym**	**Meaning**
ARDS	Acute respiratory distress syndrome
BCTU	Birmingham Clinical Trials Unit
CONSORT	Consolidated Standards of Reporting Trials
DMC	Data Monitoring Committee
ED	Emergency Department
GCS	Glasgow Comma Score
HDI	Highest Density Interval
IDS	Intervention Delivery Site
INR	International Normalised Ratio
ISRCTN	International Standard Randomised Controlled Trial Number
ITT	Intention to Treat
MCMC	Markov Chain Monte Carlo
OOR	Out of Range
PHBP	Pre-Hospital Blood Products
PHEM Team	Pre-Hospital Emergency Medical Team
PRBC	Packed Red Blood Cells
PT	Prothrombin Time
RHS	Receiving Hospital Site
ROTEM®	Rotational Thromboelastometry
SAE	Serious Adverse Event
SAP	Statistical Analysis Plan
SOFA	Sequential Organ Failure Assessment
SUSAR	Suspected Unexpected Serious Adverse Reaction
TSC	Trial Steering Committee
**Term**	**Definition**
International Standard Randomised Controlled Trial Number	A clinical trial registry
Protocol	Document that details the rationale, objectives, design, methodology and statistical considerations of the study
Randomisation	The process of assigning trial subjects to intervention or control groups using an element of chance to determine the assignments in order to reduce bias.
Statistical Analysis Plan	Pre-specified statistical methodology documented for the trial, either in the protocol or in a separate document.

TABLE OF CONTENTS

1. Introduction ... 7
2. Background and rationale ... 7
3. Trial objectives ... 7
4. Trial methods ... 8
4.1. Trial design ... 8
4.2. Trial interventions ... 8
4.3. Primary outcome measure ... 8
4.4. Secondary outcome measures ... 8
4.5. Timing of outcome assessments ... 9
4.6. Randomisation ... 9
4.7. Sample size ... 10
4.8. Framework ... 11
4.9. Interim analyses and stopping guidance ... 11
4.10. Internal Pilot Progression Rules ... 12
4.11. Timing of final analysis ... 12
4.12. Timing of other analyses ... 12
4.13. Trial comparisons ... 12
5. Statistical Principles ... 12
5.1. Confidence intervals and p-values ... 12
5.2. Adjustments for multiplicity ... 13
5.3. Analysis populations ... 13
5.4. Definition of adherence ... 13
5.5. Handling protocol deviations ... 13
5.6. Unblinding ... 14
6. Trial population ... 14
6.1. Recruitment ... 14
6.2. Baseline characteristics ... 14
7. Interventions ... 14
7.1. Description of the interventions ... 14
7.2. Adherence to allocated intervention ... 14
8. Protocol deviations ... 14
9. Analysis methods ... 15
9.1. Covariate adjustment ... 15
9.2. Distributional assumptions and outlying responses ... 15
9.3. Handling missing data ... 15
9.4. Data manipulations ... 16
9.5. Analysis methods – primary outcome(s) ... 20
9.6. Analysis methods – secondary outcomes ... 21
9.7. Analysis methods – exploratory outcomes and analyses ... 21
9.8. Safety data ... 22
9.9. Planned subgroup analyses ... 23
9.10. Sensitivity analyses ... 24
10. Analysis of sub-randomisations ... 25
11. Health economic analysis ... 25
12. Statistical software ... 25
13. References ... 25

Appendix A: Deviations from SAP ... 27
Appendix B: Trial schema ... 28
Appendix C: Schedule of assessments ... 29
Appendix D1: CONSORT flow diagram ... 30
Appendix D2: Baseline characteristics ... 31

Appendix D2a: Prehospital timeline by group 33
Appendix D2b: Mode of transport by IDS 34
Appendix D3: Description of the interventions 34
Appendix D4: Adherence to allocated intervention 34
Appendix D5: Protocol deviations 35
Appendix D6: Primary outcome results 35
Appendix D6a: Qualifying event for primary outcome 36
Appendix D7: Secondary outcomes results 37
Appendix D8: Safety 40
Appendix D9: Subgroup analysis for primary outcome 41
Appendix D10: Exploratory Bayesian analysis for primary outcome 42
Appendix D10a: Exploratory Bayesian analysis for episode mortality 43
Appendix D10b: Exploratory Bayesian analysis for lactate clearance 43

1. Introduction

This document is the Statistical Analysis Plan (SAP) for the RePHILL trial, and should be read in conjunction with the current trial protocol. This SAP details the proposed analyses and presentation of the data for the main paper(s) reporting the results for the RePHILL trial.

The results reported in these papers will follow the strategy set out here. Subsequent analyses of a more exploratory nature will not be bound by this strategy, though they are expected to follow the broad principles laid down here. The principles are not intended to curtail exploratory analysis (e.g. to decide cut-points for categorisation of continuous variables), nor to prohibit accepted practices (e.g. transformation of data prior to analysis), but they are intended to establish rules that will be followed, as closely as possible, when analysing and reporting data.

Any deviations from this SAP will be described and justified in the final report or publication of the trial (using a table as shown in Appendix A). The analysis will be carried out by an appropriately qualified statistician, who should ensure integrity of the data during their data cleaning processes.

2. Background and rationale

The background and rationale for the trial are outlined in detail in the protocol. In brief, the administration of high ratios of plasma to packed red blood cells (PRBC) has been widely adopted for in-hospital treatment of major traumatic haemorrhage. Acceptance of in-hospital haemostatic resuscitation saw the British military implement it for battlefield casualty retrieval. With the increasing adoption of giving Pre-Hospital Blood Products (PHBP) for trauma in both the UK and abroad, in both military and civilian settings, it is important to determine whether this intervention is effective. The provision of PHBP requires considerable logistical and financial resources and RePHILL will establish a high quality evidence base for PHBP resuscitation.

3. Trial objectives

The primary objective is to investigate the clinical effectiveness of PHBP resuscitation compared to the current standard care of restricted crystalloid based resuscitation in participants suffering from major traumatic haemorrhage.

Secondary objectives are to test the hypotheses that, when compared to standard care, does PHBP resuscitation:

I. Improve blood pressure, heart rate and capillary oxygenation on Emergency Department (ED) arrival?
II. Prolong on-scene time?
III. Reduce pre-hospital fluid requirements?
IV. Reduce in-hospital transfusion requirements?

V. Reduce trauma-induced coagulopathy?
VI. Preserve platelet function?
VII. Lead to a greater incidence of transfusion-related complications, particularly acute respiratory distress syndrome (ARDS)?
VIII. Lead to blood product wastage?
IX. Affect haemoglobin concentration levels on ED arrival?

4. Trial methods

4.1. Trial design

RePHILL is a multi-centre, prospective, open-label, superiority, parallel group, phase III randomised controlled trial with an internal pilot phase. See Appendix B for trial schema. The progression rules for the internal pilot are described in section 4.10.

Participants will be recruited at the scene of their traumatic injury.

4.2. Trial interventions

PHBP (Lyophilised Plasma LyoPlas N-w (LyoPlas) and PRBC) resuscitation vs. crystalloid (0.9% sodium chloride [normal saline]) resuscitation (standard care).

4.3. Primary outcome measure

The primary outcome is a composite measure consisting of episode mortality (mortality between time of injury/recruitment and discharge from the primary receiving facility to non-acute care) and lactate clearance. An event is defined as either episode mortality or a failure to achieve lactate clearance of ≥20% per hour in the first 2 hours after randomisation where a participant is considered randomised and entered into the trial when the first intervention box has been opened.

4.4. Secondary outcome measures

Secondary outcomes are as follows:

- Individual components of the primary outcome
- All-cause mortality within 3 hours of randomisation
- All-cause mortality within 30 days of randomisation
- Pre-hospital time and type and volume of fluid
- Vital signs (systolic blood pressure, heart rate, capillary oxygen saturation) at scene, on arrival at the ED, then also at 2, 6, 12 and 24 hours after arrival at ED
- Haemoglobin concentration on ED arrival

- (Venous) lactate concentration on arrival at ED and at 2 hours after arrival at ED
- Trauma-induced coagulopathy (defined as International Normalised Ratio (INR) >1.5) to be measured on arrival at ED, and also at 2 and 6 hours after arrival at ED
- Total blood product receipt at 6, 12 and 24 hours after arrival at ED
- ARDS within the first 7 days after injury
- Transfusion-related complications
- Organ failure-free days. The presence of organ failure is defined as any Sequential Organ Failure Assessment (SOFA) component score of ≥3. Organ failure will be assumed to be absent if the participant is discharged from acute care and will be assumed to be present if the participant has died.

At selected receiving hospital sites (RHS) the following secondary outcomes will also be recorded:

- Coagulation measured viscoelastically by rotational thromboelastometry (ROTEM®)
- Platelet function using multiple electrode impedence aggregometery (MultiPlate).

4.5. Timing of outcome assessments

The schedule of trial procedures and outcome assessments are given in the protocol. Secondary outcome measures will be assessed on at least one of the following time points: on-scene; 3 hours post randomisation; arrival at ED; 2, 6, 12, and 24 hours after arrival at ED; and daily assessments up to day 30.

4.6. Randomisation

Participants will be randomised at the level of the individual in a 1:1 ratio to either PHBP (Lyophilised Plasma LyoPlas N-w (LyoPlas) and PRBC) resuscitation or crystalloid (0.9% sodium chloride [normal saline]) resuscitation.

Randomisation will be performed centrally at the Birmingham Clinical Trials Unit (BCTU) using stratification by intervention delivery site (IDS) to account for variation in trauma care and type of trauma between delivery sites.

In the RePHILL trial, the blood banks will maintain a constant supply of randomised trial interventions to the Pre-Hospital Emergency Medical (PHEM) team. The blood bank will obtain the randomised allocations via a secure online system at the BCTU.

Blood banks will be supplied with pre-printed 'treatment box number' labels. A registered user at the blood bank will request a treatment allocation from the BCTU and will receive a treatment box number and treatment arm allocation. The allocated trial intervention will be packed into transport boxes affixing the correct labels. Transport boxes will be issued as a pair, one marked red and one marked yellow per single randomised allocation. The packed, sealed transport

boxes will be dispatched to the PHEM base using an established courier service as required.

On-scene, the PHEM doctor will assess the potential participant's vital signs on scene and confirm if eligible for entry into the RePHILL trial. If they fulfil the eligibility criteria for the trial, the randomised treatment will be given. Participants are considered randomised into the trial when the PHEM team open the first transport box containing the allocated trial intervention.

4.7. Sample size

Although no definitive data exists on the composite primary outcome measure, observational studies suggest potentially dramatic reductions in mortality from civilian pre-hospital PRBC[1] and military pre-hospital PRBC with thawed plasma[2]. Following extensive consultation with experts in pre-hospital trauma resuscitation, it is considered that an absolute reduction of 10% in the proportion of participants experiencing one of the component primary outcomes is clinically meaningful for the participants and is an appropriate effect size upon which to base the power calculation.

To detect an absolute difference of 10% between groups in the proportion of participants experiencing either episode mortality or lactate clearance <20%/h in the two hours post-randomisation (i.e. from 20% in the standard care group to 10% in the group receiving PHBP) using the method of difference between proportions (2-sided Fisher's Exact Test) with 80% power, and a type 1 error rate of 5% (i.e. α=0.05), requires 219 participants per group to be randomised, 438 participants in total. Assuming and adjusting for a 10% loss to follow-up rate, 490 participants will need to be recruited.

The interim analysis for the Data Monitoring Committee (DMC) meeting in May 2018 reported the results on the 192 participants recruited by 20th April 2018. A pooled event rate of 65% experiencing either episode mortality or lactate clearance <20%/h in the two hours post-randomisation was observed in these participants. This observed rate does not correspond with the pooled event rate of 15% assumed in the original sample size calculations. On the DMCs recommendations, this issue was discussed with the TSC in October 2018. The TSC recommended that the power calculations were framed in terms of a relative risk rather than an absolute risk, with the original target sample size of 490 unchanged.

Assuming the pooled event rate remains at 65% and allowing for a 10% loss to follow-up rate, 490 participants will provide 80% power to detect a relative risk ratio of 0.82 (i.e. from 71.7% in the standard care group to 58.3% in the group receiving PHBP) using the method of difference between proportions (2-sided Fisher's Exact Test), and a type 1 error rate of 5% (i.e. α=0.05). This estimated relative risk ratio is consistent with the relative risk ratios of 1.54[3] and 0.70[4] reported in two recent pre-hospital randomised controlled trials using plasma in one of the treatment arms.

4.8. Framework

The objective of the trial is to test the superiority of one intervention to another.

The null hypothesis is that there is no difference in the composite outcome of either episode mortality or a failure to achieve lactate clearance ≥20% per hour in the first 2 hours after randomisation between the intervention groups. The alternative hypothesis is that there is a difference between the groups.

4.9. Interim analyses and stopping guidance

A separate DMC reporting template will be drafted and agreed by the DMC including an agreement on which outcomes will be reported at interim analyses. The statistical methods stated in this SAP will be followed for the outcomes included in the DMC report, where possible.

If PHBP resuscitation is substantially better or worse than crystalloid resuscitation with respect to the composite outcome of episode mortality or lactate clearance, then this may become apparent before the target recruitment has been reached. Alternatively, new evidence might emerge from other sources to suggest that PHBP resuscitation is definitely more, or less, effective than crystalloid resuscitation. To protect against any unnecessary continuation of the trial, interim analyses of major endpoints and safety data will be supplied, in strict confidence, to the independent DMC, along with updates on results of other related studies, and any other analyses that the DMC may request.

The DMC will advise the chair of the Trial Steering Committee (TSC) if, in their view, any of the randomised comparisons in the trial have provided both: a) “proof beyond reasonable doubt”† that for all, or for some, types of participant one particular intervention is definitely indicated or definitely contra-indicated in terms of a net difference of a major endpoint; and b) evidence that might reasonably be expected to influence the patient management of many clinicians who are already aware of the other main trial results. Unless this happens, however, the TSC, the collaborators and all of the central trial staff (except the statisticians who supply the confidential analyses) will remain unaware of the interim results.

† Appropriate criteria of proof beyond reasonable doubt cannot be specified precisely, but a difference of at least $p<0.001$ (similar to a Haybittle-Peto[5] stopping boundary) in an interim analysis of a major endpoint may be required to justify halting, or modifying, the study prematurely. If this criterion were to be adopted, it would have the practical advantage that the exact number of interim analyses would be of little importance, so no fixed schedule is proposed. Given the proposed use of the Haybittle-Peto boundary, no adjustment for multiple testing (to control the overall type I error rate) is proposed, i.e. the threshold for statistical significance at the final analysis will still be $p=0.05$.

4.10. Internal Pilot Progression Rules

The first 6 months of the RePHILL trial will constitute an internal pilot to assess and confirm the trial logistics to determine if it is both feasible and practical to carry on and recruit into the trial. The pilot will be run at multiple sites to validate the multi-centre aspects of the trial.

At the end of the pilot phase, the following targets should be met to justify progression to the main trial:

- Minimum of 25 participants recruited across at least two active sites;
- In participants recruited to the trial intervention arm, at least one unit of PRBC and one unit of LyoPlas delivered to at least 80% of participants before reaching hospital;
- At least 90% complete data capture;
- DMC reports no safety concerns, which would prohibit continuation to main trial.

The pilot data was reviewed by the DMC and TSC on 26/APR/2017 and the trial was allowed to continue.

4.11. Timing of final analysis

The final analysis for the trial will occur once all the participants have completed the trial. The main trial data collection for participants ends at withdrawal, discharge to non-acute care, death or at 30 days follow-up, whichever occurs first. Apart from episode mortality data which is collected up to discharge from acute care which may be >30 days. Final analysis will then occur only after the corresponding outcome data has been entered onto the trial database and validated as being ready for analysis. This is provided that the trial has not been stopped early for any reason (e.g. DMC advice or funding body request).

4.12. Timing of other analyses

Not applicable.

4.13. Trial comparisons

All references in this document to 'group' refer to PHBP (Lyophilised Plasma LyoPlas N-w (LyoPlas) and PRBC) or crystalloid (0.9% sodium chloride [normal saline]) resuscitation.

5. Statistical Principles

5.1. Confidence intervals and p-values

All estimates of differences between groups will be presented with two-sided 95% confidence intervals, unless otherwise stated. P-values will be reported from two-sided tests at the 5% significance level.

5.2. Adjustments for multiplicity

No correction for multiple testing will be made.

5.3. Analysis populations

All primary analyses (primary and secondary outcomes including safety outcomes) will be based on the intention-to-treat (ITT) principle. Participants will be analysed in the intervention group to which they were randomised, and all participants shall be included whether or not they received the allocated intervention. This is to avoid any potential bias in the analysis.

A per protocol analysis will be carried out as a sensitivity analysis and will only be performed for the primary outcome. Adherence and the per protocol group are defined in section 5.4.

Further details on the sensitivity analyses are given in section 9.10.

5.4. Definition of adherence

Adherence to allocated intervention will be monitored by recording the administration of each of the four trial interventions and reasons why any of the trial interventions were not given. Blood banks will also monitor receipt of unused trial interventions upon return of the transport boxes.

We will define adherence, in the crystalloid arm of the trial, as the administration of at least one bolus of fluid. In the PHBP arm, adherence is defined as the administration of at least one unit of PRBC and one unit of LyoPlas. Non-adherence will be defined as the failure to administer at least one bolus of fluid in the crystalloid arm or the failure to administer at least one unit of PRBC and one unit of LyoPlas in the PHBP arm without clinical justification (e.g. deemed not to be needed clinically, or, patient arrives at ED before the second bolus can be administered, etc.)

The 'per protocol' population will include only those participants considered adherent to allocated intervention as described above.

5.5. Handling protocol deviations

A protocol deviation is defined as a failure to adhere to the protocol such as errors in applying the inclusion/exclusion criteria, the incorrect intervention being given, incorrect data being collected or measured, follow-up visits outside the visit window or missed follow-up visits. We will apply a strict definition of the ITT principle and will include all participants as per the ITT population described in section 5.3 in the analysis in some form, regardless of deviation from the protocol.[6] This does not include those participants who have specifically withdrawn consent for the use of their data in the first instance; however these outcomes will be explored as per other missing responses.

5.6. Unblinding

Not applicable, RePHILL is an open-label study.

6. Trial population

6.1. Recruitment

A flow diagram (as recommended by CONSORT[7]) will be produced to describe the participant flow through each stage of the trial. This will include information on the number (with reasons) of losses to follow-up (drop-outs and withdrawals) over the course of the trial. A template for reporting this is given in Appendix D1.

6.2. Baseline characteristics

The trial population will be tabulated as per Appendices D2, D2a and D2b. Categorical data will be summarised by number of participants, counts and percentages. Continuous data will be summarised by the number of participants, mean and standard deviation if deemed to be normally distributed or number of participants, median and interquartile range if data appear skewed, and ranges if appropriate. Tests of statistical significance will not be undertaken, nor confidence intervals presented.[8]

7. Interventions

7.1. Description of the interventions

Not applicable.

7.2. Adherence to allocated intervention

A cross-tabulation of allocated intervention by the adherence categories stated in section 5.4 will be produced (proportions and percentages). A template for reporting adherence is given in Appendix D4.

8. Protocol deviations

Frequencies and percentages by group will be tabulated for the protocol deviations as per Appendix D5.

9. Analysis methods

9.1. Covariate adjustment

In the first instance, intervention effects between groups for all outcomes will be adjusted for IDS (stratification variable; see section 4.6) as a fixed effect (this will be the primary analysis). A secondary analysis adjusting for IDS and the following prognostic variables (age, lactate, cardiac arrest and Glasgow Comma Score (GCS) at randomisation) as fixed effects will also be undertaken for the primary outcome measure (and the individual components).

For binary outcomes, if the log-binomial model fails to converge, a Poisson regression model with robust standard errors will be used to estimate the same parameters.[9] If this also fails to converge, unadjusted estimates will be produced from the log-binomial model. It will be made clear in the final report why this occurred (e.g. not possible due to low event rate/lack of model convergence).

Longitudinal secondary outcomes (e.g. vital signs, venous lactate concentration, etc.) will also be adjusted for their baseline values.

9.2. Distributional assumptions and outlying responses

Distributional assumptions (e.g. normality of regression residuals for continuous outcomes) will be assessed visually prior to analysis; although in the first instance the proposed primary method of estimation in this analysis plan will be followed. If responses are considered to be particularly skewed and/or distributional assumptions violated, the impact of this will be examined through sensitivity analysis; this will consist of transformation of responses prior to analysis (e.g. log transformation) in the first instance. If extreme values are apparent and considered to be affecting the integrity of the analysis, a sensitivity analysis consisting of removing the outlying response(s) and repeating the analysis will be performed. Output from these analyses, if performed, will be described and presented alongside the original analysis (or included, e.g. in appendices) with the excluded values clearly labelled.

9.3. Handling missing data

In the first instance, analysis will be completed on received data only with every effort made to follow-up participants to minimise any potential for bias. To examine the possible impact of missing data on the results, and to make sure we are complying with the intention-to-treat principle, sensitivity analysis will be performed on the primary outcome measure.[10] See section 9.10 for further details regarding planned sensitivity analyses.

9.4. Data manipulations

The Trial Statistician will derive all responses from the raw data recorded in the database.

Age

This is the number of years that occur between the date of randomisation and the date of birth and is obtained using the INTCK function in SAS v9.4. The trial only collects partial date of birth (i.e. only month and year) so it will be assumed that the participant was born on the 15th day of the month.

Date and time of randomisation
This is defined as the date and time at which the first treatment box was opened.

Time on scene
This is the number of minutes that occur between the date and time of on-scene attendance and the date and time of ED arrival, and is obtained using the INTCK function in SASv9.4. Values are reported in minutes.

Lactate clearance
The value, date, and time of the 2 hour post-randomisation lactate concentration are the value, date, and time of either the:

a) Capillary lactate concentration taken if the participant has not reached hospital within 2 hours of randomisation, or the
b) Venous lactate concentration taken in ED if the participant has reached hospital within 2 hours of randomisation, or the
c) Arterial lactate concentration taken in ED if the participant has reached hospital within 2 hours of randomisation and venous access is not available

The lactate concentration value available from a) to c) above which is closest to the 2 hours from randomisation time point will be used as the 2 hour lactate concentration value regardless of method of collection.

Using the following notation:

	=	Randomisation capillary lactate concentration
	=	2 hour post-randomisation lactate concentration
	=	Date and time of
	=	Date and time of
Interval	=	- (in minutes)

The interval time is given by:

Time between 2 hour post -randomisation lactate concentration and randomisation lactate (minutes)	=	Date and Time of the 2 hour post-randomisation lactate concentration ()	-	Date and Time of the randomisation capillary lactate concentration ()

Lactate clearance, expressed as a percentage per hour (%/h), is calculated using the formula:

A normal lactate is taken to be ≤2.2 mmol/L. Achieving ≥20% per hour lactate clearance is defined as follows in participants whose:

a) is >2.2 mmol/L and whose demonstrates lactate clearance of ≥20% per hour; or
b) is >2.2 mmol/L, but whose is ≤2.2 mmol/L, regardless of the magnitude of the change; or
c) and are both ≤2.2 mmol/L, regardless of the magnitude and direction of any difference.

All the above will be counted as participants achieving ≥20% per hour lactate clearance.

The above can be summarised in the following table:

(mmol/L)	(mmol/L)	**Required lactate clearance**
>2.2	>2.2	≥20% per hour
>2.2	≤2.2	Not applicable
≤2.2	≤2.2	Not applicable

Achieving <20% per hour lactate clearance is defined as follows in participants:

a) Whose is >2.2 mmol/L and whose demonstrates lactate clearance of <20% per hour; or
b) Who die prior to interval sampling (e.g. before the measurement is taken at). For this we require the date and time of death from the exit form to determine if the participant died within two hours and 30 minutes of randomisation.

The table below summarises what is considered an event (failure to achieve lactate clearance):

**(mmol/L)**	**(mmol/L)**	**Lactate clearance <20% per hour**	**Lactate clearance ≥20% per hour**
>2.2	>2.2	Failure to clear (event)	Achieves clearance
>2.2	≤2.2	Achieves clearance	Achieves clearance
≤2.2	≤2.2	Achieves clearance	Achieves clearance
Dies prior to interval sampling and within 2.5 hours of randomisation		Failure to clear (event)	

There are instances where the lactate value is too high for a value to be reported, i.e. the value is out of range (OOR) of the detection level of the test. In these cases, the lactate measurement is recorded on the database as “too high to be recorded”. In these instances, at database lock prior to analysis, a review of these lactate values will undertaken independently by two statisticians blind to treatment allocation to assess whether the participant cleared their lactate or not. For example, if randomisation lactate is OOR, but the 2 hour randomisation lactate is

≤2.2, then as per the table above the participant would be considered to have achieved clearance; or if both the randomisation and 2 hour lactates are OOR, then the participant will be considered to have failed to clear. If unable to determine, then the lactate component of the primary outcome will be considered missing.

Episode Mortality

Episode mortality is defined as those participants who die during the study between time of injury/recruitment and discharge from the primary receiving facility to non-acute care (this includes participants who die on-scene). The date of discharge from acute care and date of death are recorded on the exit form. Any deaths occurring after the date of discharge from acute care are not considered to be cases of episode mortality.

Primary Outcome

The primary outcome is a composite measure consisting of episode mortality (mortality between time of injury/recruitment and discharge from the primary receiving facility to non-acute care) and lactate clearance (failure to achieve lactate clearance ≥20% per hour in the first two hours from randomisation). Therefore, if the participant experiences either:

a) episode mortality **or**
b) a failure to achieve lactate clearance ≥20% per hour in the first 2 hours after randomisation

they will be considered to have experienced the primary outcome. If they have survived to the point of exiting the trial through discharge from acute care **and** have experienced lactate clearance ≥20% per hour in the first 2 hours after randomisation they will be considered to not have experienced the primary outcome.

All-cause mortality within 3 hours of randomisation

The time to death is calculated by (Date and Time of death - Date and Time of randomisation). If this is less than or equal to 3 hours then the participant will be coded as having experienced all-cause mortality within 3 hours of randomisation (i.e. ≤3 hours). If this is more than 3 hours (i.e. >3 hours), or if the participant has completed the exit form through discharge from acute care then the participant will be coded as not having experienced all-cause mortality within 3 hours of randomisation. If the Date and Time of death is not recorded, but the participant is known to have died on-scene, then it will be assumed that the participant experienced all-cause mortality within 3 hours of randomisation.

All-cause mortality within 30 days of randomisation

The time to death is calculated by (Date of death - Date of randomisation). If this is less than or equal to 30 days then the participant will be coded as having experienced all-cause mortality within 30 days of randomisation (i.e. ≤30 days). If this is more than 30 days (i.e. >30 days), or if the participant has completed the exit form through discharge from acute care (i.e. was alive at discharge from acute care) then the participant will be coded as not having experienced all-cause mortality within 30 days of randomisation. If the Date and Time of death is not recorded,

but the participant is known to have died on-scene, then it will be assumed that the participant experienced all-cause mortality within 30 days of randomisation.

Trauma-induced coagulopathy

A participant is considered to have experienced trauma-induced coagulopathy if the INR>1.5. If no INR value has been recorded, the value of prothrombin time (PT) will be converted to an INR value and used instead. To convert PT to an INR, the ratio of the observed patient PT to a control PT (standardized for the potency of the thromboplastin reagent) will be calculated:

This calculation requires the control PT to be known.

Organ failure-free days (OFFS)

The presence of organ failure is defined as any SOFA component score of ≥3. Organ failure will be assumed to be absent if the participant is discharged from acute care and will be assumed to be present if the participant has died.

The SOFA component scores are determined from the raw values using the conversions in Tables (a)-(f) below. For each daily assessment organ failure is present if:

ANY(Respiratory, Neurological, Cardiovascular, Liver, Coagulation, Renal) ≥3.

Respiratory (a)

PaO_2/FiO_2 (kPa)	Score
≥53.3	0
<53.3	1
<40.0	2
<26.7 and mechanically ventilated	3
<13.3 and mechanically ventilated	4

Neurological (b)

Glasgow Coma Scale (GCS)	Score
15	0
13-14	1
10-12	2
6-9	3
<6	4

Cardiovascular (c)

Mean Arterial Pressure or inotrope requirement	Score
MAP ≥70 mmHg	0
MAP <70 mmHg	1
dop ≤5 or dob (any dose)	2
dop >5 OR epi ≤0.1 OR nor ≤0.1	3
dop >15 OR epi>0.1 OR nor >0.1	4

Liver (d)

Bilirubin (μmol/L)	Score
<20	0
20-32	1
33-101	2
102-204	3
>204	4

Key: dop: Dopamine, dob: dobutamine, epi: adrenaline, nor: noradrenaline
Doses in [illegible]g/kg/min

Coagulation (e)

Platelets$\times 10^3/\mu l$	Score
≥150	0
<150	1
<100	2
<50	3
<20	4

Renal (f)

Creatinine (μmol/L) or urine o/p	Score
≤109	0
110-170	1
171-299	2
300-440 (or <500 mL/day)	3
> 440 (or <200 mL/day)	4

Then OFFS is the number of days alive and free of organ failure in the first 30 days. Days in which a participant is on a ward will be defined as being organ failure free. For the respiratory component, days in which a participant is either on a ward or in level 2 critical care will be defined as being ventilator free.

Age of Blood Products

For each participant allocated to the PHBP, the age of each unit of administered blood products is calculated by (Date bled – Date of randomisation). If any unit is 8 days or older, that participant will be classified as receiving blood products ≥8 days old for the purposes of the subgroup analysis (see Section 9.9).

Cardiac Arrest

Participants will be classified as experiencing cardiac arrest if they have a heart rate of 0 and a blood pressure reading (systolic or diastolic) of 0 when their vital signs are recorded on-scene.

9.5. Analysis methods – primary outcome(s)

A template for reporting the primary outcome is given in Appendix D6.

The primary outcome is a composite measure of episode mortality or failure to clear lactate and is measured as a binary outcome. Participants clearing less than 20% per hour of their lactate between randomisation and 2 hours after randomisation or dying between time of injury/recruitment and discharge to non-acute care will be defined as experiencing the primary outcome. The primary outcome measure will be summarised as the number of participants experiencing the primary outcome with percentages.

Adjusted relative risks (adjusted for IDS) along with 95% confidence intervals will be estimated using a log-binomial regression model. Statistical significance of the treatment group parameter

will be determined from the p-value generated by the model. The absolute risk difference along with 95% confidence intervals will be estimated using a binomial regression model with identity link. As this is a composite endpoint, the primary outcome will also be reported in accordance with the recommendations of Ferreira-González et al.[11], with the qualifying event for the primary outcome presented using the template in Appendix D6a.

See section 9.1 for details on covariate adjustment and model convergence.

9.6. Analysis methods – secondary outcomes

A template for reporting the secondary outcomes is given in Appendix D7.

Binary outcomes (e.g. development of ARDS, mortality at pre-specified time-points) will be analysed in the same way as the primary outcome.

Continuous outcomes (e.g. pre-hospital fluid volume, vital signs, OFFS) will be summarised using means and standard deviations, or medians and IQRs if appropriate. Adjusted mean differences (adjusted for IDS and baseline values) along with 95% confidence intervals will be estimated using a linear regression model. Statistical significance of the treatment group parameter will be determined from the p-value generated by the model.

9.7. Analysis methods – exploratory outcomes and analyses

Bayesian analysis of the primary composite outcome measure, and each component separately, will be performed as exploratory analyses to quantify the probability of treatment effects of different sizes. The PHBP and Crystalloid intervention arms will be modelled separately, with the outcome assumed to be a random deviate from a Bernoulli distribution. Priors will take the form of independent beta(α,β) distributions and are set to model the probability of a participant achieving the primary outcome in each arm. Three sets of priors are specified: non-informative priors, skeptical priors such that the probability of observing a treatment effect at least as large as the specified relative risk ratio of 0.82 is less than 5%, and informative priors reflecting current knowledge. For each set of the priors, the table below provides the values of the shape parameters used to specify the beta distributions, summary statistics (mean, variance, median, upper and lower 2.5% quantiles), and simulated probabilities of exceeding three different treatment effect sizes (expressed as relative risk ratios).

	**Priors**					
	**Non-informative**		**Skeptical**		**Informative**	
	**Crystalloid**	**PHBP**	**Crystalloid**	**PHBP**	**Crystalloid**	**PHBP**
**α**	1	1	50	50	7	3
**β**	1	1	25	25	3	2
**Mean**	0.5	0.5	0.667	0.667	0.7	0.6

**Variance**	0.0833	0.0833	0.003	0.003	0.019	0.04
**2.5%**	0.025	0.025	0.557	0.557	0.400	0.194
**Median**	0.5	0.5	0.668	0.668	0.714	0.614
**97.5%**	0.975	0.975	0.768	0.768	0.925	0.932
**Pr(RR≤0.82)**	0.410		0.0442		0.444	
**Pr(RR≤0.70)**	0.350		0.0015		0.313	
**Pr(RR≥1.54)**	0.325		0.0002		0.054	

For example, the informative prior for the PHBP group is set as beta(3,2), reflecting the assumption that the primary outcome rate in the PHBP group is unlikely to be lower than 19% or greater than 93%. For the Crystalloid group, the informative prior is set as beta(7,3), reflecting the assumption that the primary outcome rate in the Crystalloid group is unlikely to be lower than 40% or greater than 93%. Relative risk ratios of 1.54[3] and 0.70[4] were reported in two recent pre-hospital RCTs using plasma in one of the treatment arms. The informative priors are specified such that the probabilities of observing treatment effects less than or equal to 0.70 and greater than or equal to 1.54 are 31.3% and 5.4% respectively. Under the sceptical priors these probabilities are 0.15% and 0.02% respectively.

Adjusted relative risks (adjusted for IDS), along with 95% highest density intervals (HDIs) will be estimated using a Bayesian log-binomial regression model. The posterior probabilities that the relative risk ratio was less than 1, 0.8, and 0.7 will be calculated. Similarly, risk differences, adjusted for IDS, along with 95% HDIs will be estimated using a Bayesian binomial regression model with an identity link. The posterior probabilities that the risk difference was less than 0%, 10%, and 20% will be calculated. A template for reporting is given in Appendices D10 to D10b.

9.8. Safety data

The number and percentage of participants experiencing any adverse events, serious adverse events (SAEs) and suspected unexpected serious adverse reactions (SUSARs) will be presented by intervention group. Statistical significance will be determined by a chi-squared test. The total number of SAEs in each group will also be given along with a descriptive table of the details of the events. A template for reporting this data is given in Appendix D8.

9.9. Planned subgroup analyses

Interpretation of subgroup analysis will be treated with caution (output will be treated as exploratory rather than definitive[12]). Analysis will be limited to the primary outcome of a composite measure consisting of episode mortality and lactate clearance only, and the following subgroups:

- IDS
- Mode of PHEM transport (air vs. ground)
- Initial lactate concentration (≤2.2 mmol/L vs. >2.2 mmol/L)

- Time to ED from injury (≤1 hour vs. >1 hour)
- Mode of injury (blunt vs. penetrating vs. crush)
- Volume of pre-hospital fluid given (total intervention (4 boluses) .vs. <4 boluses)
- Age (<50 years, 50-70 years, >70 years)
- Head injury (positive vs. negative)
- Compressible haemorrhage (compressible haemorrhage vs. non-compressible haemorrhage)
- Pre-morbid drug history (anticoagulant or antiplatelet medication vs. no anticoagulant or antiplatelet medication)
- Age of blood products (<8 days vs. ≥8 days)
- Cardiac arrest (arrested vs. not arrested)

The effects of these subgroups will be examined by including a treatment group by subgroup interaction parameter in the regression model. A template for reporting the subgroups analyses for the primary outcome is given in Appendix D9.

The anticipated magnitude and direction of differential treatment effects in each of the subgroup analyses are:

- IDS: regional differences in PHEM protocols and system characteristics may alter the effect of the intervention. However, the anticipated direction of effect is unclear in the subgroup analysis for IDS and this analysis will be regarded as purely exploratory.
- Mode of PHEM transport (air vs. ground): ground transport typically takes longer than air transport, thus a greater effect of intervention may be observed in those being transported by ground.
- Initial lactate concentration (≤2.2 mmol/L vs. >2.2 mmol/L): participants with an abnormal initial lactate concentration (>2.2 mmol/L) may have a higher incidence of the primary outcome event and may exhibit a greater benefit from PHBP.
- Time to ED from injury (≤1 hour vs. >1 hour): participants taking >1 hour to arrive in ED from injury take longer to receive definitive treatment. Thus, a greater effect of intervention may be observed in participants taking more than >1 hour to arrive and receive definitive treatment in ED.
- Mode of injury (blunt vs. penetrating vs. crush): the anticipated direction of effect is unclear in the subgroup analyses for mode of injury and this analysis will be regarded as purely exploratory.
- Volume of pre-hospital fluid given (total intervention (4 boluses) vs. <4 boluses): the volume given is considered a surrogate for the volume of blood loss. Participants receiving <4 boluses of pre-hospital fluid may have experienced less blood loss and a lower incidence of the primary outcome event. Thus, they may exhibit a reduced effect from PHBP.
- Age (<50 years, 50-70 years, >70 years): mortality is greater in the more elderly groups. Therefore, the intervention is anticipated to have a greater effect in the >70 years cohort.

- Head injury (positive vs. negative): Major haemorrhage increases the risk of secondary brain injury. We anticipate that the intervention effect may be greater in those with head injury.
- Compressible haemorrhage (compressible haemorrhage vs. non-compressible haemorrhage): the anticipated direction of effect is unclear in the subgroup analyses for compressible haemorrhage and this analysis will be regarded as purely exploratory.
- Pre-morbid drug history (anticoagulant or antiplatelet medication vs. no anticoagulant or antiplatelet medication): the degree of haemorrhage will be greater in those with anticoagulation medication. Therefore, we anticipate that the intervention may have a greater effect in those participants on anticoagulant medication.
- Age of blood products (<8 days vs. ≥8 days): fresh blood has better oxygen carrying capacity than old blood, therefore we anticipate that the intervention may have a greater effect in those participants receiving fresher blood (<8 days old).
- Cardiac Arrest: participants experiencing a cardiac arrest may have a higher incidence of the primary outcome event, but may be less likely to demonstrate any differential treatment effect due to the severity of their condition.

9.10. Sensitivity analyses

Sensitivity analyses will be limited to the primary outcome and will consist of:

- Analysis adjusting for IDS and age, lactate, cardiac arrest and GCS at randomisation;
- Per-protocol analysis (population described in sections 5.3 and 5.4);
- An analysis to assess the effect of lactate timings. Lactate clearance is calculated on measurements made at randomisation and 2 hours post-randomisation. For this sensitivity analysis, time windows will be applied to the lactate measurements. The lactate concentration at randomisation will only contribute to this analysis if the measurement was taken no more than 15 minutes (i.e. ≤15 minutes) before the PHEM team open the first transport box containing the allocated trial intervention. The lactate concentration at 2 hours post randomisation will only contribute to this analysis if the measurement was taken within 30 minutes of the 2 hour assessment (i.e. between 1.5 and 2.5 hours post-randomisation). Lactate concentrations recorded outside these times will not be included in this sensitivity analysis.
- An analysis to assess the effect of missing responses; missing responses will be simulated using a Markov chain Monte Carlo method (MCMC) to generate multiple datasets. The imputation model will include the following variables: IDS, age, randomisation lactate, cardiac arrest, GCS, haemorrhage type, category of injury (penetrating, blunt, crush), heart rate, systolic blood pressure, and injury severity score. Analysis will be then be performed on each set with the results combined using Rubin's rule to obtain a single set of results (treatment effect estimate and confidence intervals). Each component of the primary outcome will be imputed separately[13,14].
- An analysis to assess the generalisability of the results by comparing baseline characteristics of those who are lost to follow-up or withdrawn from the trial with the trial

population.
- An analysis to assess the generalisability of the results by comparing baseline characteristics of those who provide primary outcome data to those who do not provide primary outcome data.

10. Analysis of sub-randomisations

Not Applicable.

11. Health economic analysis

No health economic analysis is planned for this trial.

12. Statistical software

Statistical analysis will be undertaken in the following statistical software packages: SAS v9.4 and RStan v2.19.

13. References

1. Brown, J.B., Cohen, M.J., Minei, J.P., Maier, R.V., West, M.A., Billiar, T.R., Peitzman, A.B., Moore, E.E., Cuschieri, J. and Sperry, J.L. Pretrauma center red blood cell transfusion is associated with reduced mortality and coagulopathy in severely injured patients with blunt trauma. Annals of Surgery. 2015; 261(5): 997-1005.
2. O'Reilly, D.J., Morrison, J.J., Jansen, J.O., Apodaca, A.N., Rasmussen, T.E. and Midwinter, M.J. Prehospital blood transfusion in the en route management of severe combat trauma: A matched cohort study. Journal of Trauma and Acute Care Surgery. 2014; 77(3 Suppl 2): S114-20.
3. Moore HB, Moore EE, Chapman MP, et al. Plasma-first resuscitation to treat haemorrhagic shock during emergency ground transportation in an urban area: a randomised trial. Lancet 2018;392:283-91
4. Sperry JL, Guyette FX, Brown JB, et al. Prehospital Plasma during Air Medical Transport in Trauma Patients at Risk for Hemorrhagic Shock. N Engl J Med 2018;379:315-26.
5. Peto R, Pike MC, Armitage P, Breslow NE, Cox DR, Howard SV et al. Design and analysis of randomised clinical trials requiring prolonged observation of each patient. I. Introduction and design. Br J Cancer. 1976;34:585-612.
6. Gupta SK. Intention-to-treat concept: A review. Perspect Clin Res. 2011;2(3):109-112.
7. Schulz KF, Altman DG, Moher D, for the CONSORT Group. CONSORT 2010 Statement: updated guidelines for reporting parallel group randomised trials. BMJ. 2010;340:c332.
8. Altman DG, Dore CJ. Randomisation and baseline comparisons in clinical trials. Lancet. 1990;335:149–53.
9. Zou G. A modified Poisson regression approach to prospective studies with binary data. Am J Epidemiol. 2004;159(7):702-6.

10. White IR, Horton NJ, Carpenter J, Pocock SJ. Strategy for intention to treat analysis in randomised trials with missing outcome data. BMJ. 2011;342:d40.
11. Ferreira-Gonzalez, I., Permanyer-Miralda, G., Busse, J.W., Bryant, D.M., Montori, V.M., Alonso-Coello, P., Walter, S.D. and Guyatt, G.H. Methodologic discussions for using and interpreting composite endpoints are limited, but still identify major concerns. Journal of Clinical Epidemiology. 2007; 60(7): 651-7.
12. Wand R, Lagakos SW, Ware JH, Hunter DJ, Drazen JM. Reporting of subgroups analyses in clinical trials. NEJM. 2007;357:2189-94.
13. O'Keeffe AG, Farewell DM, Tom BD, Farewell VT. Multiple imputation of missing composite outcomes in longitudinal data. Statistics in biosciences. 2016 Oct 1;8(2):310-32.
14. Sullivan TR, White IR, Salter AB, Ryan P, Lee KJ. Should multiple imputation be the method of choice for handling missing data in randomized trials?. Statistical methods in medical research. 2018 Sep;27(9):2610-26.

Appendix A: Deviations from SAP

This report below follows the statistical analysis plan dated <insert effective date of latest SAP> apart from following:

**Section of report not following SAP**	**Reason**
<insert section >	<insert, e.g. exploratory analyses request by TMG>

Appendix B: Trial schema

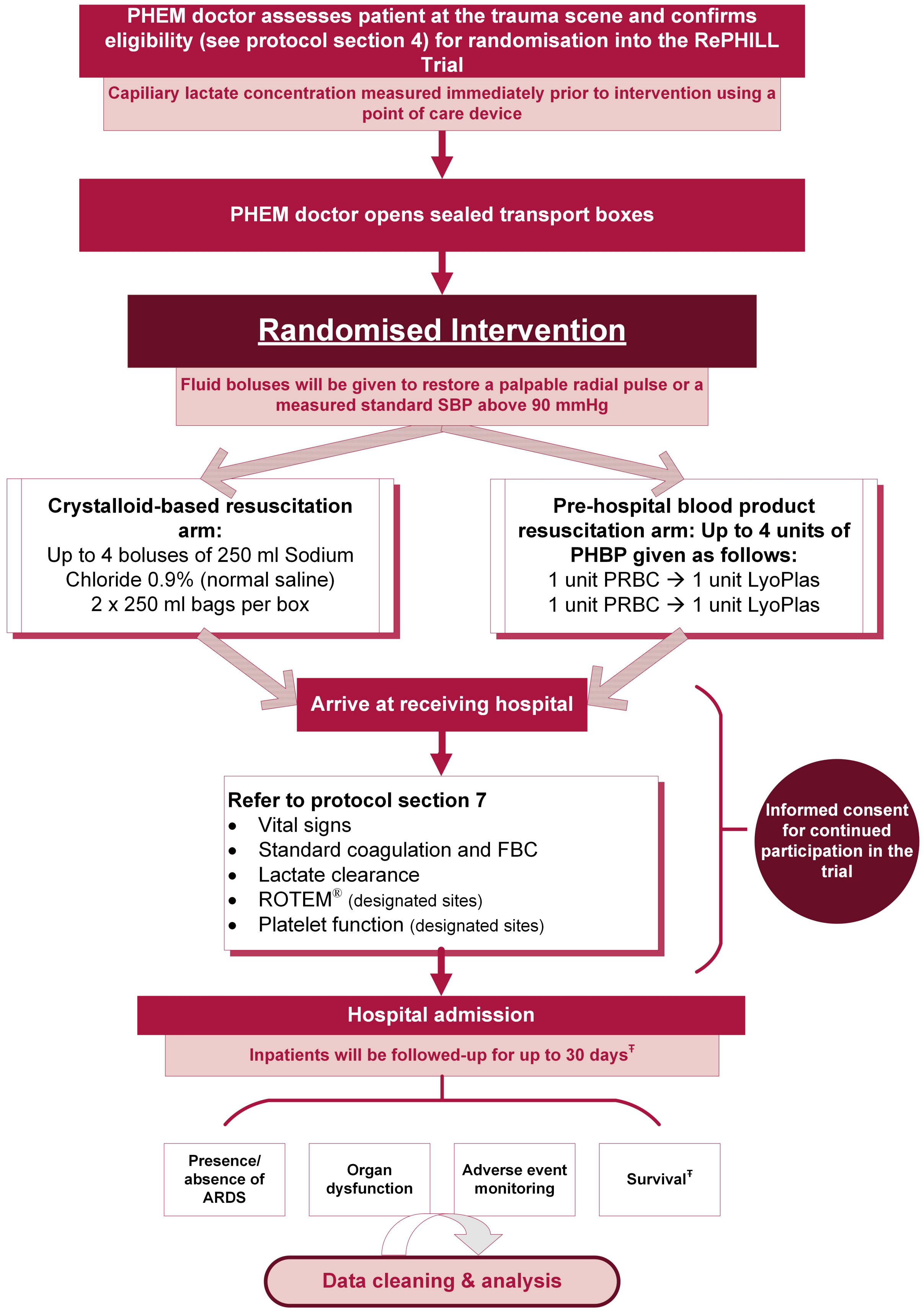

‡ Episode mortality data will be collected up to discharge from the acute care setting which may be >30 days

Appendix C: Schedule of assessments

See trial protocol.

Appendix page 87

Appendix D1: CONSORT flow diagram

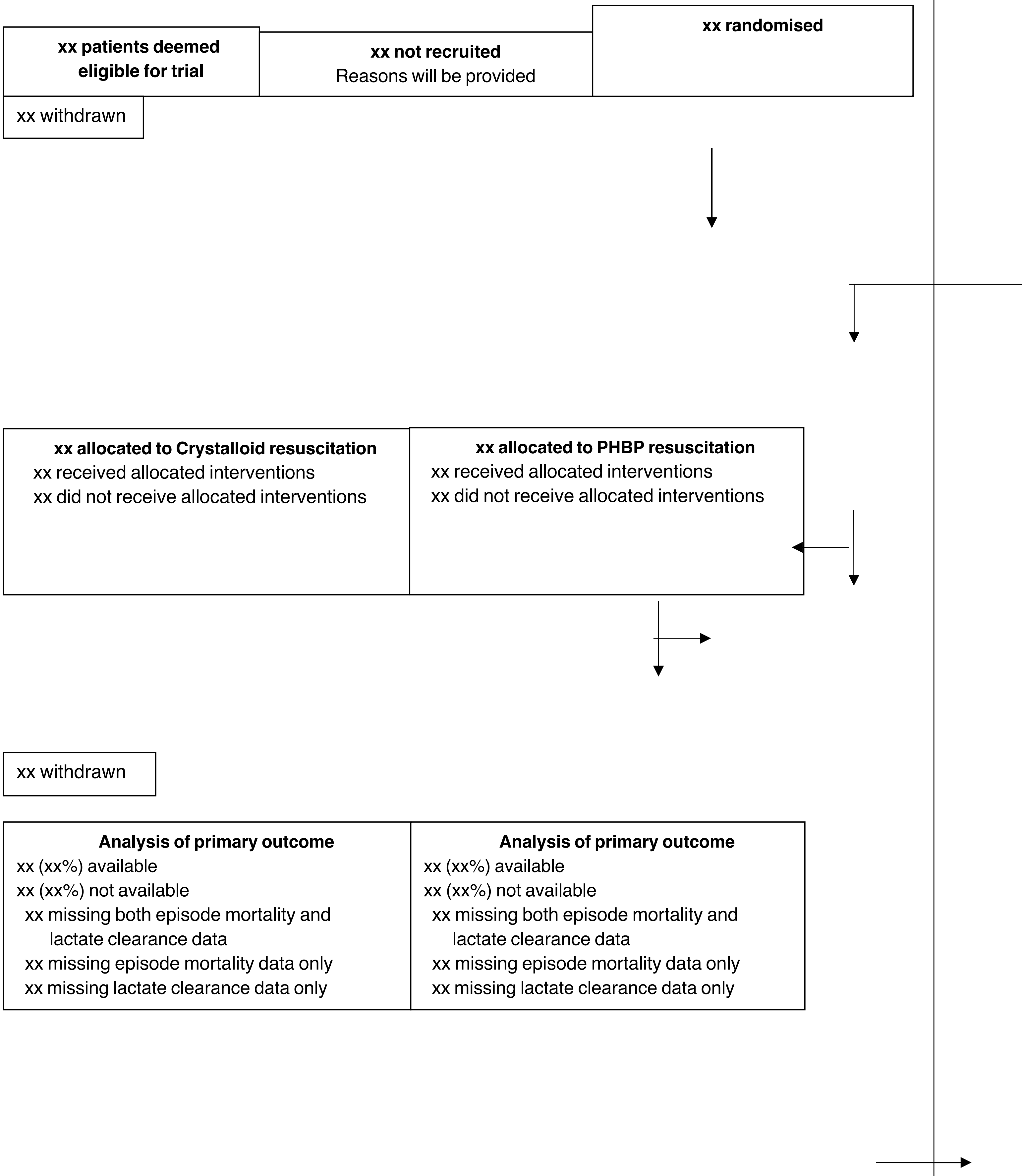

Appendix D2: Baseline characteristics

		PHBP (n=xxx)	Crystalloid (n=xxx)	Overall (n=xxx)
**Stratification variable**				
Intervention Delivery Site	Midlands Air Ambulance (MAA)	n (%)	n (%)	n (%)
	MAGPAS	n (%)	n (%)	n (%)
	The Air Ambulance Service (TAAS)	n (%)	n (%)	n (%)
	East Anglian Air Ambulance (EAAA)	n (%)	n (%)	n (%)
	...			
**Demographic and other baseline variables**				
Gender	Male	n (%)	n (%)	n (%)
	Female	n (%)	n (%)	n (%)
	Missing	n (%)	n (%)	n (%)
Age, years	Mean (SD)	...	...	...
	Missing	n (%)	n (%)	n (%)
Ethnic Group, n (%)	White (British)	n (%)	n (%)	n (%)
	White (Irish)	n (%)	n (%)	n (%)
	White (Other)	n (%)	n (%)	n (%)
	Black (Caribbean)	n (%)	n (%)	n (%)
	Missing	n (%)	n (%)	n (%)
Capillary lactate concentration (mmol/L)	Mean (SD)	...	...	...
	Missing	n (%)	n (%)	n (%)
Mechanism of injury(ies) (More than one can apply)	Fall from <2m	n (%)	n (%)	n (%)
	Fall from ≥2m	n (%)	n (%)	n (%)
	Inhalation	n (%)	n (%)	n (%)
	Road traffic accident	n (%)	n (%)	n (%)
	Burn injury	n (%)	n (%)	n (%)
	Head Injury	n (%)	n (%)	n (%)
	Gunshot wound	n (%)	n (%)	n (%)
	Stabbing	n (%)	n (%)	n (%)
	Other	n (%)	n (%)	n (%)
	Missing	n (%)	n (%)	n (%)
Acute Brain Injury	Yes	n (%)	n (%)	n (%)
	No	n (%)	n (%)	n (%)
	Missing	n (%)	n (%)	n (%)
Mode of transport	Air	n (%)	n (%)	n (%)
	Ground	n (%)	n (%)	n (%)
	Missing	n (%)	n (%)	n (%)
Haemorrhage	Compressible	n (%)	n (%)	n (%)
	Non-compressible	n (%)	n (%)	n (%)
	Missing	n (%)	n (%)	n (%)
Category of injury	Blunt	n (%)	n (%)	n (%)
	Penetrating	n (%)	n (%)	n (%)
	Crush	n (%)	n (%)	n (%)
	Missing	n (%)	n (%)	n (%)
Tranexamic Acid				
Has the participant been given a tranexamic acid bolus?	Yes	n (%)	n (%)	n (%)
	No	n (%)	n (%)	n (%)
	Missing	n (%)	n (%)	n (%)
On-scene Vital Signs				
Systolic Blood Pressure[1] (mmHg)	Zero	n (%)	n (%)	n (%)
	Non-zero	n (%)	n (%)	n (%)
	Missing	n (%)	n (%)	n (%)

	Mean (SD)	...	...	...
Diastolic Blood Pressure[1] (mmHg)	Zero	n (%)	n (%)	n (%)
	Non-zero	n (%)	n (%)	n (%)
	Missing	n (%)	n (%)	n (%)
	Mean (SD)	...	...	...
Heart Rate[1] (bpm)	Zero	n (%)	n (%)	n (%)
	Non-zero	n (%)	n (%)	n (%)
	Missing	n (%)	n (%)	n (%)
	Mean (SD)	...	...	...
Respiratory Rate[1] (/min)	Zero	n (%)	n (%)	n (%)
	Non-zero	n (%)	n (%)	n (%)
	Missing	n (%)	n (%)	n (%)
	Mean (SD)	...	...	...
Cardiac Arrest[2]	Yes	n (%)	n (%)	n (%)
	No	n (%)	n (%)	n (%)
	Missing	n (%)	n (%)	n (%)
Capillary oxygen saturation (SpO_2) (%)	Mean (SD)	...	...	...
	Missing	n (%)	n (%)	n (%)
GCS	Median (IQR)	...	...	...
	Missing	n (%)	n (%)	n (%)
Suspected at time of injury	Alcohol	n (%)	n (%)	n (%)
	Other illicit substances	n (%)	n (%)	n (%)
	Missing	n (%)	n (%)	n (%)
**Medical History**				
ISS[3]	Median (IQR)	...	...	...
	Missing	n (%)	n (%)	n (%)
NISS[3]	Median (IQR)	...	...	...
	Missing	n (%)	n (%)	n (%)
Comorbidities	Yes	n (%)	n (%)	n (%)
	No	n (%)	n (%)	n (%)
	Missing	n (%)	n (%)	n (%)
Anticoagulant medication	Yes	n (%)	n (%)	n (%)
	No	n (%)	n (%)	n (%)
	Unknown	n (%)	n (%)	n (%)
	Missing	n (%)	n (%)	n (%)
Antiplatelet medication	Yes	n (%)	n (%)	n (%)
	No	n (%)	n (%)	n (%)
	Unknown	n (%)	n (%)	n (%)
	Missing	n (%)	n (%)	n (%)

[1] Systolic blood pressure, Heart rate and Respiratory rate are summarised as continuous variables only for participants with non-zero on scene measurements

[2] Defined at those with a heart rate of 0 and blood pressure of 0

[3] ISS and NISS will only be available for those participants who are TARN eligible, hence the number of missing participants refers to the number of TARN eligible participants missing their ISS or NISS.

Appendix D2a: Prehospital timeline by group

The duration in minutes between the time of the 999 call and prehospital events. Values above the diagonal correspond to the PHBP group and values below the diagonal correspond to the Crystalloid group.

Prehospital event	999 Call	On-scene attendance	Randomisation Capillary Lactate	Treatment box opening	Left Scene*	Arrival at ED**
999 call		Median (IQR) (n=N)	Median (IQR) (n=N)	Median (IQR) (n=N)	Median (IQR)	Median (IQR)

					(n=N)	(n=N)
**On-scene attendance**	Median (IQR) (n=N)		Median (IQR) (n=N)	Median (IQR) (n=N)	Median (IQR) (n=N)	Median (IQR) (n=N)
**Randomisation Capillary Lactate**	Median (IQR) (n=N)	Median (IQR) (n=N)		Median (IQR) (n=N)	Median (IQR) (n=N)	Median (IQR) (n=N)
**Treatment box opening**	Median (IQR) (n=N)	Median (IQR) (n=N)	Median (IQR) (n=N)		Median (IQR) (n=N)	Median (IQR) (n=N)
**Left Scene***	Median (IQR) (n=N)	Median (IQR) (n=N)	Median (IQR) (n=N)	Median (IQR) (n=N)		Median (IQR) (n=N)
**Arrival at ED****	Median (IQR) (n=N)	Median (IQR) (n=N)	Median (IQR) (n=N)	Median (IQR) (n=N)	Median (IQR) (n=N)	

*Added in v4.0 of pre-hospital CRF (active in all sites from 29 th August 2019)

**These numbers are lower than the totals of randomised participants due to deaths on-scene or during transit to hospital.

Appendix D2b: Mode of transport by IDS

**Mode of transport**	**Midlands Air Ambulance (MAA) (N=)**	**MAGPAS (N=)**	**The Air Ambulance Service (TAAS) (N=)**	**East Anglian Air Ambulance (EAAA) (N=)**	**...**
**Air**	n (%)	n (%)	n (%)	n (%)	
**Ground**	n (%)	n (%)	n (%)	n (%)	
**Missing**	n (%)	n (%)	n (%)	n (%)	

Appendix D3: Description of the interventions

Not applicable.

Appendix D4: Adherence to allocated intervention

		**PHBP**	**Crystalloid**	**Total**
**Number received intervention (n)** [1]				
	**Yes, n(%)**	n (%)	n (%)	n (%)
	**No, n(%)**	n (%)	n (%)	n (%)
	**Missing, n(%)**	n (%)	n (%)	n (%)
**No. units of PRBC given**				
	**0, n(%)**	n (%)		n (%)
	**1, n(%)**	n (%)		n (%)
	**2, n(%)**	n (%)		n (%)
	**Missing, n(%)**	n (%)		n (%)

**No. units of LyoPlas given**				
	**0, n(%)**	n (%)		n (%)
	**1, n(%)**	n (%)		n (%)
	**2, n(%)**	n (%)		n (%)
	**Missing, n(%)**	n (%)		n (%)
**No. units of saline given**				
	**0, n(%)**		n (%)	n (%)
	**1, n(%)**		n (%)	n (%)
	**2, n(%)**		n (%)	n (%)
	**Missing, n(%)**		n (%)	n (%)
**Total number of units given (n)**				
	**0, n(%)**	n (%)	n (%)	n (%)
	**1, n(%)**	n (%)	n (%)	n (%)
	**2, n(%)**	n (%)	n (%)	n (%)
	**3, n(%)**	n (%)	n (%)	n (%)
	**4, n(%)**	n (%)	n (%)	n (%)
	**Missing n(%)**	n (%)	n (%)	n (%)
**Total number of units given (n)**				
	**All 4 units, n(%)**	n (%)	n (%)	n (%)
	**1-3, n(%)**	n (%)	n (%)	n (%)
	**No units, n(%)**	n (%)	n (%)	n (%)
	**Missing**	n (%)	n (%)	n (%)
**Per-protocol population (n)**				
**Received at least one unit of allocated intervention[2], n(%)**	**Yes, n(%)**	n (%)	n (%)	n (%)
	**No, n(%)**	n (%)	n (%)	n (%)

[1] This is the number of participants receiving at least one unit of intervention.

[2] The per-protocol population is defined in section 5.4

Reasons for non-adherence will be provided.

Appendix D5: Protocol deviations

Protocol deviation	PHBP (N=)	Crystalloid (N=)
...		
...		
...		

Appendix D6: Primary outcome results

The primary outcome is a composite measure consisting of episode mortality and lactate clearance.

Composite outcome, n (%)	PHBP (N=)	Crystalloid (N=)	Relative Risk[1] (95%CI)	p-value

Yes				
No				
Missing				

[1]Output from log-binomial regression model adjusted for IDS. Values of relative risk <1 indicate fewer negative events (episode mortality or failure to clear lactate concentration) with PHBP.

Composite outcome, n (%)	PHBP (N=)	Crystalloid (N=)	Absolute Risk Difference[1] (95%CI)	p-value
Yes				
No				
Missing				

[1]Output from a binomial regression model with an identity link adjusted for IDS. Values of absolute risk difference <0 indicate fewer negative events (episode mortality or failure to clear lactate concentration) with PHBP.

Appendix D6a: Qualifying event for primary outcome

Qualifying Event for Primary Outcome	PHBP (N=)	Crystalloid (N=)	Total (N=)
Both episode mortality and failure to clear lactate	n (%)	n (%)	n (%)
Episode mortality alone	n (%)	n (%)	n (%)
Failure to clear lactate alone	n (%)	n (%)	n (%)
Missing/Not available	n (%)	n (%)	n (%)

Appendix D7: Secondary outcomes results

Outcome	Time-point	PHBP (N=)	Crystalloid (N=)	Adjusted treatment effect and 95%CI	p-value
Episode Mortality[1]	Up to discharge from acute care	n/N (%)	n/N (%)	Relative risk (95% CI)[2]	p-value
Episode Mortality[1]	Up to discharge from acute care	n/N (%)	n/N (%)	Absolute risk difference (95% CI)[3]	
Failure to achieve lactate clearance[4]	2 hours post-randomisation	n/N (%)	n/N (%)	Relative risk (95% CI)[5]	p-value
Failure to achieve lactate clearance[4]	2 hours post-randomisation	n/N (%)	n/N (%)	Absolute risk difference (95% CI)[6]	
All-cause mortality	Within 3 hours of randomisation	n/N (%)	n/N (%)	Relative risk (95% CI)[7]	p-value
All-cause mortality	Within 3 hours of randomisation	n/N (%)	n/N (%)	Absolute risk difference (95% CI)[8]	
All-cause mortality	Within 3 hours of randomisation[9]	n/N (%)	n/N (%)	Relative risk (95% CI)[7]	p-value
All-cause mortality	Within 3 hours of randomisation[9]	n/N (%)	n/N (%)	Absolute risk difference (95% CI)[8]	
All-cause mortality	Within 30 days of randomisation	n/N (%)	n/N (%)	Relative risk (95% CI)[7]	p-value
All-cause mortality	Within 30 days of randomisation	n/N (%)	n/N (%)	Absolute risk difference (95% CI)[8]	
Pre-hospital time	Time to ED arrival from 999 call	(N=..) Mean (SD)	(N=..) Mean (SD)	Mean difference (95% CI)[10]	p-value
Pre-hospital time	Time to ED arrival from randomisation	(N=..) Mean (SD)	(N=..) Mean (SD)	Mean difference (95% CI)[10]	p-value
**Pre-hospital fluid type and volume**					
Type & Total volume of Fluids given prior to intervention	Prior to intervention up to ED arrival	(N=..) Mean (SD)	(N=..) Mean (SD)	Mean difference (95% CI)[11]	p-value
Type & Total volume of Fluids given post intervention	Post intervention up to ED arrival	(N=..) Mean (SD)	(N=..) Mean (SD)	Mean difference (95% CI)[11]	p-value
**Vital Signs**					
Systolic blood pressure	On-scene, ED arrival, 2, 6, 12, 24 hours after ED arrival	(N=..) Mean (SD)	(N=..) Mean (SD)	Mean difference (95% CI)[12]	p-value
Diastolic blood pressure	On-scene, ED arrival, 2, 6, 12, 24 hours after ED arrival	(N=..) Mean (SD)	(N=..) Mean (SD)	Mean difference (95% CI)[12]	p-value
Heart rate	On-scene, ED arrival, 2, 6, 12, 24 hours after ED	(N=..) Mean (SD)	(N=..) Mean (SD)	Mean difference (95% CI)[13]	p-value

	arrival				
Capillary Oxygen Saturation	On-scene, ED arrival, 2, 6, 12, 24 hours after ED arrival	(N=..) Mean (SD)	(N=..) Mean (SD)	Mean difference (95% CI)[14]	p-value
Respiratory rate	On-scene, ED arrival, 2, 6, 12, 24 hours after ED arrival	(N=..) Mean (SD)	(N=..) Mean (SD)	Mean difference (95% CI)[15]	p-value
**Other**					
Venous/arterial lactate concentration	2 hours after randomisation, ED arrival, 2 hours after ED arrival	(N=..) Mean (SD)	(N=..) Mean (SD)	Mean difference (95% CI)[16]	p-value
Trauma-induced coagulopathy (INR>1.5)	ED arrival, 2, 6 hours after arrival at ED	n/N (%)	n/N (%)	Relative risk (95% CI)[17]	p-value
Haemoglobin concentration	ED arrival	(N=..) Mean (SD)	(N=..) Mean (SD)	Mean difference (95% CI)[18]	p-value
Cumulative total blood product receipt	6, 12 and 24 hours after arrival at ED	Mean (SD)	Mean (SD)	Mean difference (95% CI)[11]	p-value
ARDS	Within 7 days after ED admission	n/N (%)	n/N (%)	Relative risk (95% CI)[19]	p-value
Transfusion-related complications	24 hours after arrival at ED	n/N (%)	n/N (%)	Relative risk (95% CI)[20]	p-value
Organ Failure Free Days (OFFS)	Up to day 30	(N=..) Mean (SD)	(N=..) Mean (SD)	Mean difference (95% CI)[21]	p-value
**ROTEM**					
**EXTEM**					
A05 (mm)	First blood sample taken in hospital	(N=..) Mean (SD)	(N=..) Mean (SD)	Mean difference (95% CI)[22]	p-value
CFT (seconds)	First blood sample taken in hospital	(N=..) Mean (SD)	(N=..) Mean (SD)	Mean difference (95% CI)[23]	p-value
MCF (mm)	First blood sample taken in hospital	(N=..) Mean (SD)	(N=..) Mean (SD)	Mean difference (95% CI)[24]	p-value
CT (seconds)	First blood sample taken in hospital	(N=..) Mean (SD)	(N=..) Mean (SD)	Mean difference (95% CI)[25]	p-value
α angle (degree)	First blood sample taken in hospital	(N=..) Mean (SD)	(N=..) Mean (SD)	Mean difference (95% CI)[26]	p-value
Ly30 (%)	First blood sample taken in hospital	(N=..) Mean (SD)	(N=..) Mean (SD)	Mean difference (95% CI)[27]	p-value
Ly60 (%)	First blood sample taken in hospital	(N=..) Mean (SD)	(N=..) Mean (SD)	Mean difference (95% CI)[28]	p-value
**FIBTEM**					
A05 (mm)	First blood sample taken in hospital	(N=..) Mean (SD)	(N=..) Mean (SD)	Mean difference (95% CI)[22]	p-value
CFT (seconds)	First blood sample taken in hospital	(N=..) Mean (SD)	(N=..) Mean (SD)	Mean difference (95% CI)[23]	p-value
MCF (mm)	First blood sample taken in hospital	(N=..) Mean (SD)	(N=..) Mean (SD)	Mean difference (95% CI)[24]	p-value
CT (seconds)	First blood sample taken in hospital	(N=..) Mean (SD)	(N=..) Mean (SD)	Mean difference (95% CI)[25]	p-value
α angle (degree)	First blood sample taken in hospital	(N=..) Mean (SD)	(N=..) Mean (SD)	Mean difference (95% CI)[26]	p-value
Ly30 (%)	First blood sample taken in hospital	(N=..) Mean (SD)	(N=..) Mean (SD)	Mean difference (95% CI)[27]	p-value

Ly60 (%)	First blood sample taken in hospital	(N=..) Mean (SD)	(N=..) Mean (SD)	Mean difference (95% CI)[28]	p-value
**Multiplate (Area under Curve)**					
TRAP	First blood sample taken in hospital	(N=..) Mean (SD)	(N=..) Mean (SD)	Mean difference (95% CI)[29]	p-value
ADP	First blood sample taken in hospital	(N=..) Mean (SD)	(N=..) Mean (SD)	Mean difference (95% CI)[29]	p-value
ASPI	First blood sample taken in hospital	(N=..) Mean (SD)	(N=..) Mean (SD)	Mean difference (95% CI)[29]	p-value
**Exploratory**					
ITU length of stay	Up to discharge from ITU	(N=..) Mean (SD)	(N=..) Mean (SD)	Mean difference (95% CI)[30]	
Hospital length of stay	Up to discharge from hospital	(N=..) Mean (SD)	(N=..) Mean (SD)	Mean difference (95% CI)[30]	
**Any Organ Failure by system (SOFA ≥3)**					
Respiratory	During hospital stay (up to day 30)	n/N (%)	n/N (%)	Relative risk (95% CI)[31]	
Neurological	During hospital stay (up to day 30)	n/N (%)	n/N (%)	Relative risk (95% CI)[31]	
Cardiovascular	During hospital stay (up to day 30)	n/N (%)	n/N (%)	Relative risk (95% CI)[31]	
Liver	During hospital stay (up to day 30)	n/N (%)	n/N (%)	Relative risk (95% CI)[31]	
Coagulation	During hospital stay (up to day 30)	n/N (%)	n/N (%)	Relative risk (95% CI)[31]	
Renal	During hospital stay (up to day 30)	n/N (%)	n/N (%)	Relative risk (95% CI)[31]	
Use of tranexamic acid	2, 6, 12 and 24 hours after arrival at ED	n/N (%)	n/N (%)	Relative risk (95% CI)[32]	
Surgery	2, 6, 12 and 24 hours after arrival at ED	n/N (%)	n/N (%)	Relative risk (95% CI)[33]	

[1]mortality between time of injury/recruitment and discharge from the primary receiving facility to non-acute care
[2]Relative risk < 1 indicates fewer episode mortality events with PHBP
[3]Absolute risk difference < 0 indicates fewer episode mortality events with PHBP
[4]a failure to achieve lactate clearance ≥ 20% per hour in the first 2 hours after randomisation
[5]Relative risk < 1 indicates fewer lactate clearance failures with PHBP
[6]Absolute risk difference < 0 indicates fewer lactate clearance failures with PHBP
[7]Relative risk < 1 indicates fewer mortality events with PHBP
[8]Absolute risk difference < 0 indicates fewer mortality events with PHBP
[9]Based only on participants providing an exact time of death.
[10]Difference < 0 indicates shorter time from 999 call or randomisation to ED arrival with PHBP.
[11]Difference < 0 indicates less fluid administered with PHBP.
[12]Difference > 0 indicates higher blood pressure with PHBP.
[13]Difference > 0 indicates higher heart rate with PHBP.
[14]Difference > 0 indicates higher capillary oxygen saturation with PHBP.
[15]Difference > 0 indicates higher respiratory rate with PHBP.
[16]Difference < 0 indicates lower venous lactate concentration with PHBP.
[17]Relative risk < 1 indicates lower rate of trauma-induced coagulopathy with PHBP
[18]Difference < 0 indicates lower haemoglobin concentration with PHBP.
[19]Relative risk < 1 indicates lower rate of ARDS with PHBP
[20] Relative risk < 1 indicates lower rate of transfusion-related complications with PHBP.
[21]Difference > 0 indicates higher number of days free from organ failure with PHBP

[22] Difference > 0 indicates higher amplitude after 5 minutes with PHBP.
[23]Difference < 0 indicates lower clot formation time with PHBP.
[24]Difference > 0 indicates higher maximum clot firmness with PHBP.
[25]Difference < 0 indicates lower coagulation time with PHBP.
[26]Difference < 0 indicates lower speed of clot formation time with PHBP.
[27]Difference < 0 indicates lower lysis index at 30 minutes with PHBP.
[28]Difference < 0 indicates lower lysis index at 60 minutes with PHBP.
[29]Difference > 0 indicates higher agonist AUC with PHBP.
[30]Difference > 0 indicates longer length of stay with PHBP.
[31]Relative risk < 1 indicates fewer organ failure events with PHBP.
[32]Relative risk < 1 indicates lower rate of tranexamic acid use with PHBP.
[33]Relative risk < 1 indicates lower rate of surgery with PHBP.

Appendix D8: Safety

	PHBP (N=)	Crystalloid (N=)	p-value
Transfusion-related lung injury	n (%)	n (%)	p-value
Any thromboembolism	n (%)	n (%)	p-value
Deep-vein thrombosis	n (%)	n (%)	p-value
Pulmonary embolism	n (%)	n (%)	p-value
Stroke	n (%)	n (%)	p-value
Other	n (%)	n (%)	p-value
Infection (suspicion, or clinical evidence of)	n (%)	n (%)	p-value
Intra-abdominal	n (%)	n (%)	p-value
Meningitis	n (%)	n (%)	p-value
Respiratory	n (%)	n (%)	p-value
UTI	n (%)	n (%)	p-value
Soft tissue	n (%)	n (%)	p-value
Indwelling device	n (%)	n (%)	p-value
Blood-born	n (%)	n (%)	p-value
Other	n (%)	n (%)	p-value

	PHBP (N=)	Crystalloid (N=)	p-value
Total number of SAEs	n	n	
Total number of participants experiencing an SAE	n (%)	n (%)	<insert p-value>
Total number of SUSARs	n	N	
Total number of participants experiencing an SUSAR	n (%)	n (%)	<insert p-value>

A line by line listing of each SAE may be appropriate for some studies and can be included in an Appendix to the main report; the table below provides a guide for how the line listing of SAE data could be presented.

Summary of SAE	Reason for Reporting	Causality	Action taken
**PHBP**			
1 <insert description of			

SAE>				
2				
3				
4				
**Crystalloid**				
1				
2				
3				
4				

Appendix D9: Subgroup analysis for primary outcome

Subgroup description	PHBP (N=)	Crystalloid (N=)	Adjusted relative risk (95% CI)	p-value for interaction
**IDS**				
Site 1	n (%)	n (%)	Relative risk (95% CI)[1]	p-value
Site 2	n (%)	n (%)	Relative risk (95% CI)[1]	
Site 3	n (%)	n (%)	Relative risk (95% CI)[1]	
Site 4	n (%)	n (%)	Relative risk (95% CI)[1]	
**Mode of transport**				
Air	n (%)	n (%)	Relative risk (95% CI)[1]	p-value
Ground	n (%)	n (%)	Relative risk (95% CI)[1]	
**Initial Lactate Concentration**				

≤2.2 mmol/L	n (%)	n (%)	Relative risk (95% CI)[1]	p-value
>2.2 mmol/L	n (%)	n (%)	Relative risk (95% CI)[1]	
**Cardiac Arrest**				
Yes	n (%)	n (%)	Relative risk (95% CI)[1]	p-value
No	n (%)	n (%)	Relative risk (95% CI)[1]	
**Time to ED from injury**				
≤ 1 hour	n (%)	n (%)	Relative risk (95% CI)[1]	p-value
>1 hour	n (%)	n (%)	Relative risk (95% CI)[1]	
**Mode of injury**				
Blunt	n (%)	n (%)	Relative risk (95% CI)[1]	p-value
Penetrating	n (%)	n (%)	Relative risk (95% CI)[1]	
Crush	n (%)	n (%)	Relative risk (95% CI)[1]	
**Volume of pre-hospital fluid given**				
4 units	n (%)	n (%)	Relative risk (95% CI)[1]	p-value
< 4 units	n (%)	n (%)	Relative risk (95% CI)[1]	
**Age**				
<50 years	n (%)	n (%)	Relative risk (95% CI)[1]	p-value
50 – 70 years	n (%)	n (%)	Relative risk (95% CI)[1]	
>70 years	n (%)	n (%)	Relative risk (95% CI)[1]	
**Head Injury**				
Positive	n (%)	n (%)	Relative risk (95% CI)[1]	p-value
Negative	n (%)	n (%)	Relative risk (95% CI)[1]	
**Compressible Haemorrhage**				
Compressible Haemorrhage	n (%)	n (%)	Relative risk (95% CI)[1]	p-value
Non-Compressible Haemorrhage	n (%)	n (%)	Relative risk (95% CI)[1]	
**Pre-morbid drug history**				
Anticoagulant/antiplatelet medication	n (%)	n (%)	Relative risk (95% CI)[1]	p-value
No anticoagulant/antiplatelet medication	n (%)	n (%)	Relative risk (95% CI)[1]	
**Age of blood products**				
<8 days	n (%)	n (%)	Relative risk (95% CI)[1]	p-value
≥8 days	n (%)	n (%)	Relative risk (95% CI)[1]	

[1]Output from log-binomial regression model. Values of relative risk <1 indicate lower rate of negative events (episode mortality or failure to clear lactate concentration) with PHBP.

Appendix D10: Exploratory Bayesian analysis for primary outcome

The primary outcome is a composite measure consisting of episode mortality and lactate clearance.

PHBP (N=)	Crystalloid (N=)	Median Relative Risk Ratio	95% HDI	Probability of Relative Risk Ratio		
				< 1.0	< 0.8	<0.7

[1]Output from Bayesian log-binomial regression model adjusted for IDS. Values of relative risk <1 indicate fewer negative events (episode mortality or failure to clear lactate concentration) with PHBP.

PHBP (N=)	Crystalloid (N=)	Median Risk Difference	95% HDI	Probability of Risk Difference		
				< 0%	< -10%	< -20%

[1]Output from Bayesian binomial regression model with an identity link adjusted for IDS. Values of risk difference < 0 indicate fewer negative events (episode mortality or failure to clear lactate concentration) with PHBP.

Appendix D10a: Exploratory Bayesian analysis for episode mortality

PHBP (N=)	Crystalloid (N=)	Median Relative Risk Ratio	95% HDI	Probability of Relative Risk Ratio		
				< 1.0	< 0.8	<0.7

[1]Output from Bayesian log-binomial regression model adjusted for IDS. Values of relative risk <1 indicate fewer events of episode mortality with PHBP.

PHBP (N=)	Crystalloid (N=)	Median Risk Difference	95% HDI	Probability of Risk Difference		
				< 0%	< -10%	< -20%

[1]Output from Bayesian binomial regression model with an identity link adjusted for IDS. Values of risk difference < 0 indicate fewer events of episode mortality with PHBP.

Appendix D10b: Exploratory Bayesian analysis for lactate clearance

PHBP (N=)	Crystalloid (N=)	Median Relative Risk Ratio	95% HDI	Probability of Relative Risk Ratio		
				< 1.0	< 0.8	<0.7

[1]Output from Bayesian log-binomial regression model adjusted for IDS. Values of relative risk <1 indicate fewer failures to clear lactate concentration with PHBP.

PHBP (N=)	Crystalloid (N=)	Median Risk Difference	95% HDI	Probability of Risk Difference		
				< 0%	< -10%	< -20%

[1]Output from Bayesian binomial regression model with an identity link adjusted for IDS. Values of risk difference < 0 indicate fewer failures to clear lactate concentration with PHBP.

S3 Further Secondary and Exploratory Outcomes

Further Secondary and Exploratory Outcomes that do not appear in the main manuscript are presented in Table 1.

Table 1: Secondary and Exploratory Outcomes

Outcome	PRBC / LyoPlas	0·9% saline	Adjusted risk ratio (95% CI)	Adjusted average difference (95% CI)
**Pre-hospital fluid type and volume**				
Fluids given prior to intervention	142/209 68%)	159/223 (71%)	0.95 (0.83, 1.07)[1]; P=0.40	
Saline[5]	140/209 (67%)	159/223 (71%)		
Hartmann's[5]	1/209 (0.5%)	2/223 (1%)		
Other[5]	7/209 (3%)	4/223 (2%)		
Volume given prior to intervention	422 (499), 209	437 (482), 223		-17 (-108, 74)[2]; 0.71
Fluids given after intervention	40/207 (19%)	52/221 (24%)	0.84 (0.58, 1.21)[1]; P=0.35	
Saline[5]	33/207 (16%)	39/221 (17%)		
Hartmann's[5]	3/207 (1%)	6/221 (3%)		
Other[5]	5/207 (2%)	15/221 (7%)		
Volume given after intervention	123 (310), 207	160 (389), 221		-34 (-101, 32)[2]; P=0.31
**Vital signs**				
**Heart Rate (bpm)**				
On scene	115 (31) ,185	109 (33), 198		5.83 (-0.61, 12.27)[2]; P=0.08
ED arrival	107 (29), 157	105 (24), 154		-0.80 (-5.83, 4.23)[3]; P=0.76
2 hrs after ED arrival	95 (22), 147	91 (22), 147		3.80 (-1.09, 8.70)[3]; P=0.13
6 hrs after ED arrival	88 (21), 148	86 (21), 137		2.57 (-2.34, 7.49)[3]; P=0.31
12 hrs after ED arrival	90 (21), 149	89 (23), 139		1.23 (-3.81, 6.28)[3]; P=0.63
24 hrs after ED arrival	90 (20), 144	90 (22), 134		-1.05 (-5.94, 3.84)[3]; P=0.67
**Systolic Blood Pressure (mmHg)**				
On scene	73 (16), 128	73 (20), 148		-0.05 (-4.23, 4.14)[2]; P=0.98
ED arrival	114 (27), 111	114 (29), 124		-1.19 (-8.19, 5.82)[3]; P=0.74
2 hrs after ED arrival	114 (24), 113	115 (21), 121		0.04 (-5.75, 5.83)[3]; P=0.99
6 hrs after ED arrival	109 (21), 116	114 (23), 117		-5.22 (-10.87, 0.43)[3]; P=0.07
12 hrs after ED arrival	113 (22), 110	115 (24), 118		-2.27 (-8.23, 3.69)[3]; P=0.45
24 hrs after ED arrival	114 (20), 109	117 (21), 114		-3.24 (-8.59, 2.12)[3]; P=0.24
**Diastolic Blood Pressure (mmHg)**				

Outcome	PRBC / LyoPlas	0·9% saline	Adjusted risk ratio (95% CI)	Adjusted average difference (95% CI)
On scene	47 (13), 125	46 (16), 147		0.77 (-2.70, 4.24)[2]; P=0.66
ED arrival	75 (24), 107	72 (24), 123		2.26 (-3.77, 8.29)[3]; P=0.46
2 hrs after ED arrival	67 (17), 111	65 (15), 119		2.07 (-1.97, 6.12)[3]; P=0.31
6 hrs after ED arrival	64 (15), 114	67 (15), 117		-2.76 (-6.57, 1.04)[3]; P=0.15
12 hrs after ED arrival	62 (13), 108	62 (13), 118		-0.36 (-3.60, 2.88)[3]; P=0.83
24 hrs after ED arrival	61 (14), 107	62 (12), 114		-1.44 (-4.73, 1.84)[3]; P=0.36
**Respiratory Rate (/min)**				
On scene	24 (9.5), 172	23 (10.6), 191		0.98 (-1.10, 3.05)[2]; P=0.36
ED arrival	20 (6.5), 128	19 (5.6), 126		0.59 (-0.79, 1.97)[3]; P=0.40
2 hrs after ED arrival	19 (4.8), 121	19 (4.7), 123		0.45 (-0.72, 1.62)[3]; P=0.45
6 hrs after ED arrival	19 (6.3), 133	18 (4.1), 129		0.62 (-0.66, 1.91)[3]; P=0.34
12 hrs after ED arrival	19 (5.2), 140	18 (3.8), 133		0.49 (-0.59, 1.58)[3]; P=0.37
24 hrs after ED arrival	18 (4.11), 140	18 (3.7), 129		0.38 (-0.56, 1.31)[3]; P=0.43
**Oxygen Saturation (%)**				
On scene	92 (7.6) ,131	91 (9.3), 144		0.92 (-1.10, 2.94)[2]; P=0.37
ED arrival	97 (5.2), 105	97 (5.2), 114		0.48 (-0.86, 1.82)[3]; P=0.48
2 hrs after ED arrival	98 (3.9), 104	98 (4.9), 108		0.03 (-1.14, 1.20)[3]; P=0.96
6 hrs after ED arrival	98 (4.4), 109	98 (6.0), 103		0.48 (-0.94, 1.90)[3]; P=0.51
12 hrs after ED arrival	97 (6.9), 108	98 (3.9), 102		-0.38 (-1.91, 1.15)[3]; P=0.63
24 hrs after ED arrival	97 (2.6), 105	98 (2.4), 96		-0.02 (-0.70, 0.65)[3]; P=0.95
**Laboratory Results**				
**Lactate Concentration (mmol/L)**				
2 hours post-randomisation based on time	5.42 (4.45) (n=168)	5.78 (4.68) (n=169)		-0.37 (-1.28, 0.53)[3]; P=0.42
2 hours post-randomisation based on CRF	4.91 (4.14) (n=153)	5.40 (4.41) (n=152)		-0.34 (-1.24, 0.55)[3]; P=0.46
Arrival at ED	7.04 (4.50) (n=157)	6.93 (4.58) (n=161)		-0.08 (-0.97, 0.82)[3]; P=0.87
2 hrs after ED arrival	4.45 (3.57) (n=134)	4.46 (3.33) (n=138)		-0.07 (-0.84, 0.70)[3]; P=0.86
**International normalised ratio (INR) >1.5**				
ED arrival	12/84 (14%)	12/74 (16%)	0.91 (0.44, 1.90)[1]; P=0.80	

Outcome	PRBC / LyoPlas	0·9% saline	Adjusted risk ratio (95% CI)	Adjusted average difference (95% CI)
2 hrs after ED arrival	1/27 (4%)	4/29 (14%)	0.27 (0.03, 2.25)[4]; P=0.23	
6 hrs after ED arrival	3/48 (6%)	3/46 (7%)	0.81 (0.17, 3.88)[1]; P=0.79	
**Haemoglobin (g/L)** arrival at ED	133 (19), 154	118 (23), 152		15 (10, 19)[2]; P=<0.0001
**Calcium (mmol/L)** arrival at ED	1.21 (0.42), 152	1.24 (0.37), 156		-0.03 (-0.12, 0.05)[2]; P=0.44
**Total Blood Product Receipt**				
**Red Blood Cells (Units)**				
6 hrs after arrival at ED	5.61 (5.92), 132	5.31 (5.84), 137		0.09 (-1.27, 1.45)[2]; P=0.89
12 hrs after arrival at ED	6.03 (7.62), 144	5.26 (6.08), 143		0.55 (-1.00, 2.10)[2]; P=0.49
24 hrs after arrival at ED	5.63 (6.14), 139	5.31 (6.33), 134		0.18 (-1.22, 1.59)[2]; P=0.80
**Plasma (units)**				
6 hrs after arrival at ED	4.31 (4.68), 143	3.97 (4.75), 144		0.16 (-0.90, 1.22)[2]; P=0.77
12 hrs after arrival at ED	4.72 (5.69), 144	4.26 (5.17), 143		0.30 (-0.92, 1.51)[2]; P=0.63
24 hrs after arrival at ED	4.50 (4.76), 139	4.31 (5.40), 134		0.12 (-1.03, 1.26)[2]; P=0.84
**Crystalloid (volume)**				
6 hrs after arrival at ED	1417 (1610), 142	1037 (1175), 144		382 (61, 702)[2]; P=0.02
12 hrs after arrival at ED	2388 (2031), 143	1782 (1550), 143		628 (221, 1034)[2]; P=0.003
24 hrs after arrival at ED	3620 (2479), 139	2947 (2115), 134		708 (180, 1236)[2]; P=0.009
**Cryoprecipitate (bags)**				
6 hrs after arrival at ED	0.66 (1.23), 143	0.64 (1.38), 144		0.001 (-0.30, 0.30)[2]; P=0.99
12 hrs after arrival at ED	0.89 (1.73), 144	0.80 (1.65), 143		0.05 (-0.33, 0.43)[2]; P=0.79
24 hrs after arrival at ED	0.96 (2.03), 139	0.88 (2.00), 134		0.06 (-0.41, 0.52)[2]; P=0.82
**Platelets (bags)**				
6 hrs after arrival at ED	0.54 (0.97), 143	0.42 (0.87), 144		0.10 (-0.11, 0.31)[2]; P=0.37
12 hrs after arrival at ED	0.63 (1.14), 144	0.55 (1.02), 143		0.06 (-0.18, 0.31)[2]; P=0.62
24 hrs after arrival at ED	0.71 (1.19), 139	0.67 (1.31), 134		0.02 (-0.27, 0.31)[2]; P=0.90
**Colloid (volume)**				
6 hrs after arrival at ED	28 (155), 142	83 (317), 144		-55 (-113, 3)[2]; P=0.06
12 hrs after arrival at ED	31 (144), 143	128 (499), 142		-98 (-183, -13)[2]; P=0.02
24 hrs after arrival at ED	105 (368), 138	197 (701), 134		-89 (-221, 43)[2]; P=0.18
**ARDS**	9/142 (6%)	3/129 (2%)	2.71 (0.75, 9.81)[1]; P=0.13	

Outcome	PRBC / LyoPlas	0·9% saline	Adjusted risk ratio (95% CI)	Adjusted average difference (95% CI)
**Transfusion-related complications (in first 24 hours in ED)**	11/148 (7%)	9/137 (7%)	1.05 (0.46, 2.42)[1]; P=0.90	
**Organ failure-free days[a],**	12.9 (13.0), 202	12.1 (13.1), 212		0.86 (-1.64, 3.36)[2]; P=0.50
**ROTEM**				
**EXTEM**				
A05 (mm)	35.8 (9.9), 32	33.2 (11.9), 23		2.61 (-3.07, 8.29)[2]; P=0.37
CFT (seconds)	107 [84.5, 131.5], 32	110 [79, 145], 22		-3 (-36, 30)[5]; P=0.86
MCF (mm)	55.7 (12.4) ,32	54.9 (6.01), 20		0.64 (-5.10, 6.37)[2]; P=0.83
CT (seconds)	78 [73, 107], 33	78 [69, 122], 3		3 (-22, 28)[4]; P=0.81
α angle (degree)	70 [66, 73], 28	71 [65, 74] ,23		-1 (-6, 4)[4]; P=0.67
Ly30 (%)	100 [100, 100] ,32	100 [100, 100], 23		0 (-0, 0)[5]; -
Ly60 (%)	99.5 [99, 100], 22	98.5 [97, 100], 18		1 (-0.29, 2.29)[4]; P=0.13
**FIBTEM**				
A05 (mm)	8.73 (3.78), 30	5.86 (2.71), 22		2.89 (1.06, 4.71)[2]; P=0.002
CFT (seconds)	76 (-), 1	-		-
MCF (mm)	12.0 (9.6), 29	7.85 (3.34), 20		4.21 (-0.13, 8.54)[2]; P=0.06
CT (seconds)	73 [67, 101], 31	84 [70, 121], 22		-9 (-33, 15)[4]; P=0.46
α angle (degree)	63 (7.3), 15	60 (9.0), 7		2.51 (-3.90, 8.93)[2]; P=0.44
Ly30 (%)	100 [100, 100], 29	100 [100, 100], 22		0 (-0, 0)[5]; -
Ly60 (%)	100 [100, 100], 21	100 [100, 100], 17		0 (0, 0)[5]; -
**Multiplate**				
TRAP	93.4 (49.8), 21	77.6 (44.2), 10		11.0 (-24.2, 46.2)[2]; P=0.54
ADP	53.5 (40.4), 22	42.8 (24.6), 10		6.36 (-19.7, 32.4)[2]; P=0.63
ASPI	66.2 (41.8), 21	51.4 (36.5), 10		12.8 (-17.1, 42.7)[2]; P=0.40
**All-cause mortality ≤ 3 hrs of randomisation**				
Using time of death only	6/171 (4%)	6/168 (4%)	0.94 (0.32, 2.82)[1]; P=0.92	-0.001 (-0.04, 0.04)[6]; P=0.98

Data are n/N (%); mean (SD); median [IQR]; or mean (SD), N, or median [IQR], N, when N is different to the total number of participants, unless otherwise specified.
ARDS = Acute Respiratory Distress Syndrome, EXTEM = Tissue factor activation, FIBTEM = Tissue factor activation + platelet inhibition evaluating the contribution of fibrinogen to clot formation, PRBC = Packed Red Blood Cells, ROTEM = Rotational Thromboelastometry.

[a]Organ failure-free days. The presence of organ failure is defined as any Sequential Organ Failure Assessment component score of ≥ 3. Organ failure will be assumed to be absent if the participant is discharged from hospital and will be assumed to be present if the participant has died.
[1]Output is from a log-binomial regression model adjusted for IDS. Values of risk ratio <1 indicate lower event rates in the PRBC / LyoPlas group.
[2]Output is from a linear regression model adjusted for IDS. Values of mean differences <0 indicate lower average values the PRBC / LyoPlas group
[3]Output is from a linear regression model adjusted for IDS and the on scene value of the outcome variable. Values of mean differences <0 indicate lower average values in PRBC / LyoPlas group.
[4]Output is from a quantile regression model adjusted for IDS. Values of median differences <0 indicate lower average values in the PRBC / LyoPlas group.
[5]Output is from Hodges-Lehmann estimation of the location shift between the two groups and asymptotic confidence intervals. Estimates are not adjusted for IDS. Values of mean differences <0 indicate lower average values in the PRBC / LyoPlas group.
[6]Output is from a binomial regression model with identity link adjusted for IDS. Values of absolute risk difference <0 lower event rates in the PRBC / LyoPlas group.

Exploratory Bayesian Analyses

Bayesian models were fitted using three different prior distributions: non-informative prior, sceptical prior such that the probability of observing a treatment effect at least as large as the specified relative risk ratio of 0.82 is less than 5%, and informative prior reflecting current knowledge. For each set of the priors, the table below provides summary statistics (the mean value, and upper and lower 2.5% quantiles) of the primary outcome event rates in each treatment group.

Table 2: Priors for Bayesian analysis of primary outcome

**Prior**	**Treatment Group**	**Summary Statistics for Prior Distributions**		
		**2.5%**	**Mean**	**97.5%**
**Non-informative**	0·9% saline	2.5%	50%	97.5%
	PRBC / LyoPlas	2.5%	50%	97.5%
**Sceptical**	0·9% saline	55.7%	66.7%	76.8%
	PRBC / LyoPlas	55.7%	66.7%	76.8%
**Informative**	0·9% saline	40%	70%	93.2%
	PRBC / LyoPlas	19.4%	60%	92.5%

PRBC = Packed Red Blood Cells

Bayesian Analysis of the Composite Primary Outcome

For each set of priors, the median risk ratios and associated 95% higher posterior density intervals for the primary outcome are presented in Table 3, along with the posterior probabilities that the risk ratio is less than 1, 0.8, and 0.7.

Table 3: Bayesian analysis of primary outcome using all recorded 2hr post-randomisation lactates: Risk Ratio adjusted for IDS

**Composite Outcome**	**PRBC / LyoPlas (N=209)**	**0·9% saline (N=223)**	**Priors**	**Median Risk Ratio**	**95% HDI**	**Probability of Risk Ratio**		
						**< 1.0**	**< 0.8**	**<0.7**
Yes	128 (64%)	136 (65%)	Non-informative	1.01[1]	(0.88, 1.16)[1]	43.5%	0.1%	0%
No	71 (36%)	74 (35%)	Sceptical	1.01[1]	(0.87, 1.16)[1]	44%	0.1%	0%
Missing	10	13	Informative	1.01[1]	(0.87, 1.16)[1]	44%	0.1%	0%

PRBC = Packed Red Blood Cells
[1]Output from Bayesian log-binomial regression model adjusted for IDS. Values of risk ratio <1 indicate fewer negative events (episode mortality or failure to clear lactate concentration) with PRBC / LyoPlas.

For each set of priors, the median absolute risk differences and associated 95% higher posterior density intervals for the primary outcome are presented in Table 4, along with the posterior probabilities that the absolute risk difference is less than 0, -10%, and -20%.

Table 4: Bayesian analysis of primary outcome using all recorded 2hr post-randomisation lactates: Absolute Risk Difference adjusted for IDS

Composite Outcome	PRBC / LyoPlas (N=209)	0·9% saline (N=223)	Priors	Median Absolute Risk Difference	95% HDI	Probability of Absolute Risk Difference		
						< 0.0	< -0.1	< -0.2
Yes	128 (64%)	136 (65%)	Non-informative	0.002[1]	(-0.09, 0.09)[1]	48.2%	1.3%	0.007%
No	71 (36%)	74 (35%)	Sceptical	0.006[1]	(-0.07, 0.08)[1]	44.1%	0.3%	0%
Missing	10	13	Informative	-0.004[1]	(-0.09, 0.08)[1]	53.4%	1.6%	0%

PRBC = Packed Red Blood Cells

[1]Output from Bayesian binomial regression model with identity link adjusted for IDS. Values of absolute risk difference <0 indicate fewer negative events (episode mortality or failure to clear lactate concentration) with PRBC / LyoPlas.

Bayesian Analysis of Episode Mortality

For each set of priors, the median risk ratios and associated 95% higher posterior density intervals for Episode Mortality are presented in Table 5, along with the posterior probabilities that the risk ratio is less than 1, 0.8, and 0.7.

Table 5: Bayesian analysis of episode mortality: Risk Ratio adjusted for IDS

Episode Mortality	PRBC / LyoPlas (N=209)	0·9% saline (N=223)	Priors	Median Risk Ratio	95% HDI	Probability of Risk Ratio		
						< 1.0	< 0.8	<0.7
Yes	88 (43%)	99 (45%)	Non-informative	0.97[1]	(0.77, 1.18)[1]	63.1%	4.2%	0.2%
No	115 (57%)	119 (55%)	Sceptical	0.97[1]	(0.77, 1.17)[1]	63.6%	4.6%	0.2%
Missing	6	5	Informative	0.97[1]	(0.77, 1.17)[1]	64.0%	4.6%	0.2%

PRBC = Packed Red Blood Cells

[1]Output from Bayesian log-binomial regression model adjusted for IDS. Values of risk ratio <1 indicate fewer episode mortality events with PRBC / LyoPlas.

For each set of priors, the median absolute risk differences and associated 95% higher posterior density intervals for Episode Mortality are presented in Table 6, along with the posterior probabilities that the absolute risk difference is less than 0, -10%, and -20%.

Table 6: Bayesian analysis of episode mortality: Absolute Risk Difference adjusted for IDS

Episode Mortality	PRBC / LyoPlas (N=209)	0·9% saline (N=223)	Priors	Median Absolute Risk Difference	95% HDI	Probability of Absolute Risk Difference		
						< 0.0	< -0.1	< -0.2
Yes	88 (43%)	99 (45%)	Non-informative	-0.03[1]	(-0.12, 0.06)[1]	71.2%	5.9%	0.009%
No	115 (57%)	119 (55%)	Sceptical	-0.05[1]	(-0.12, 0.03)[1]	88.2%	8.4%	0%
Missing	6	5	Informative	-0.04[1]	(-0.13, 0.05)[1]	80.7%	9.5%	0.013%

PRBC = Packed Red Blood Cells

[1]Output from Bayesian binomial regression model with identity link adjusted for IDS. Values of absolute risk difference <0 indicate fewer episode mortality events with PRBC / LyoPlas.

Bayesian Analysis of Failure to Clear Lactate

For each set of priors, the median risk ratios and associated 95% higher posterior density intervals for Failure to clear lactate are presented in Table 7, along with the posterior probabilities that the risk ratio is less than 1, 0.8, and 0.7.

Table 7: Bayesian analysis of failure to clear lactate: Risk Ratio adjusted for IDS

Failure to clear lactate	PRBC / LyoPlas (N=209)	0·9% saline (N=223)	Priors	Median Risk Ratio	95% HDI	Probability of Risk Ratio		
						< 1.0	< 0.8	<0.7
Yes	98 (50%)	113 (55%)	Non-informative	0.94[1]	(0.78, 1.13)[1]	73.5%	4.3%	0.09%
No	98 (50%)	93 (45%)	Skeptical	0.94[1]	(0.77, 1.12)[1]	74.4%	4.4%	0.14%
Missing	13	17	Informative	0.94[1]	(0.77, 1.12)[1]	74.5%	4.5%	0.12%

PRBC = Packed Red Blood Cells

[1]Output from Bayesian log-binomial regression model adjusted for IDS. Values of risk ratio <1 indicate fewer failures to clear lactate with PRBC / LyoPlas.

For each set of priors, the median absolute risk differences and associated 95% higher posterior density intervals for Failure to clear lactate are presented in Table 8, along with the posterior probabilities that the absolute risk difference is less than 0, -10%, and -20%.

Table 8: Bayesian analysis of failure to clear lactate: Absolute Risk Difference adjusted for IDS

Failure to clear lactate	PRBC / LyoPlas (N=209)	0·9% saline (N=223)	Priors	Median Absolute Risk Difference	95% HDI	Probability of Absolute Risk Difference		
						< 0.0	< -0.1	< -0.2
Yes	98 (50%)	113 (55%)	Non-informative	-0.04[1]	(-0.13, 0.05)[1]	81.3%	11.5%	0.05%
No	98 (50%)	93 (45%)	Skeptical	-0.04[1]	(-0.11, 0.04)[1]	84.6%	5.6%	0%
Missing	13	17	Informative	-0.05[1]	(-0.14, 0.04)[1]	86.9%	15.3%	0.07%

PRBC = Packed Red Blood Cells

[1]Output from Bayesian binomial regression model with identity link adjusted for IDS. Values of absolute risk difference <0 indicate fewer failures to clear lactate with PRBC / LyoPlas.

S4 Sensitivity Analyses

Sensitivity analyses for the primary outcome are presented in Table 9.

Table 9: Sensitivity Analyses for Primary Outcome

PRBC / LyoPlas	0·9% saline	Sensitivity Analysis	Adjusted Risk Ratio (95% CI); p-value	Adjusted Risk Difference (95% CI); p-value
128/199 (64%)	136/210 (65%)	Original analysis	1.01 (0.88, 1.17)[1]; P=0.86	-0.00025 (-0.09, 0.09)[2]; P=0.996
77/130 (59%)	87/151 (58%)	Further Covariate adjustment	1.02 (0.95, 1.08)[3]; P=0.63	0.02 (-0.10, 0.13)[4]; P=0.79
125/192 (65%)	130/203 (64%)	Per-protocol analysis	1.04 (0.90, 1.20)[1]; P=0.63	0.014 (-0.08, 0.11)[2]; P=0.76
125/196 (64%)	126/198 (64%)	Secondary per-protocol analysis	1.03 (0.89, 1.19)[1]; P=0.68	0.012 (-0.08, 0.11)[2]; P=0.80
95/151 (65%)	103/153 (67%)	Timing of Lactate Concentration	0.99 (0.85, 1.17)[1]; P=0.94	-0.014 (-0.12, 0.09)[2]; P=0.80
-	-	Missing responses	1.01 (0.87, 1.17)[5]; P=0.90	-0.001 (-0.094, 0.091)[6]; P=0.98

PRBC: Packed Red Blood Cells

[1]Output is from a log-binomial regression model adjusted for IDS. Values of risk ratio <1 indicate fewer negative events (episode mortality or failure to clear lactate concentration) with PRBC / LyoPlas.

[2]Output is from a binomial regression model with an identity link adjusted for IDS. Values of absolute risk difference <0 indicate fewer negative events (episode mortality or failure to clear lactate concentration) with PRBC / LyoPlas.

[3]Log-binomial regression failed due to lack of model convergence. Output is from a Poisson regression model with robust standard error adjusted for IDS, age, capillary lactate, cardiac arrest, and GCS. Values of risk ratio <1 indicate fewer negative events (episode mortality or failure to clear lactate concentration) with PRBC / LyoPlas.

[4]Binomial regression with an identity link adjusted for IDS, age, capillary lactate, cardiac arrest, and GCS failed due to lack of model convergence. Output is from a binomial regression model with an identity link adjusted only for IDS. Values of absolute risk difference <0 indicate fewer negative events (episode mortality or failure to clear lactate concentration) with PRBC / LyoPlas.

[5]Output is from estimates pooled over 50 imputed datasets, each analysed using a log-binomial regression model adjusted for IDS. Values of risk ratio <1 indicate fewer negative events (episode mortality or failure to clear lactate concentration) with PRBC / LyoPlas.

[6]Output is from estimates pooled over 50 imputed datasets, each analysed using a binomial regression model with identity link adjusted for IDS. Values of absolute risk difference <0 indicate fewer negative events (episode mortality or failure to clear lactate concentration) with PRBC / LyoPlas.

S5 Post-hoc subgroup analyses

The post-hoc subgroup analyses, for the Primary Outcome, of time from scene to arrival at ED (< 20 minutes .vs. ≥ 20 minutes) and injury severity (NISS bands of <16 .vs. 16-30 .vs. >30) are displayed in Figure 1.

	PRBC / LyoPlas (n/N)	0·9% saline (n/N)	Risk Ratio [95% CI]	p-value
**Transport Time (Scene to ED)**				
< 20 minutes	12 / 17	8 / 15	1·12 (0·91-1·37)	0·64
≥ 20 minutes	20 / 33	22 / 39	1·05 (0·91-1·22)	
**Injury Severity (NISS)**				
< 16	1 / 7	1 / 7	0·95 (0·07-12·30)	
16 to 30	11 / 26	8 / 24	1·32 (0·64-2·70)	0·82
> 30	68 / 107	69 / 111	1·04 (0·86-1·27)	
**All Patients**	**128 / 199**	**136 / 210**	**1·01 (0·88-1·17)**	**0·86**

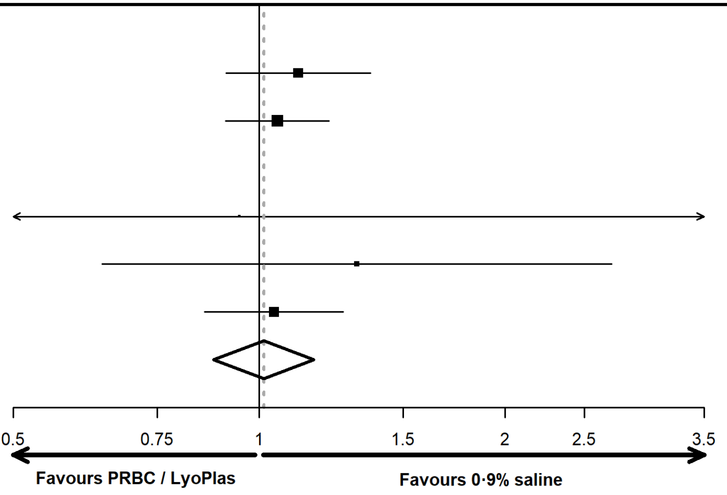

Figure 1: Post-hoc subgroup analyses of Primary Outcome

S6 Intervention Delivery Sites

A list of intervention delivery sites and principal investigators are provided in Table 10.

Table 10: Intervention Delivery Sites and Principal Investigators

Intervention Delivery Site	Principal Investigator
East Anglian Air Ambulance	Alistair Wilson
MAGPAS Air Ambulance	Simon Lewis
The Air Ambulance Service	Caroline Leech
West Midlands Air Ambulance	Mark Nash

S7 Haemoglobin Concentration on ED arrival

Figure 3: Haemoglobin concentration on ED arrival.

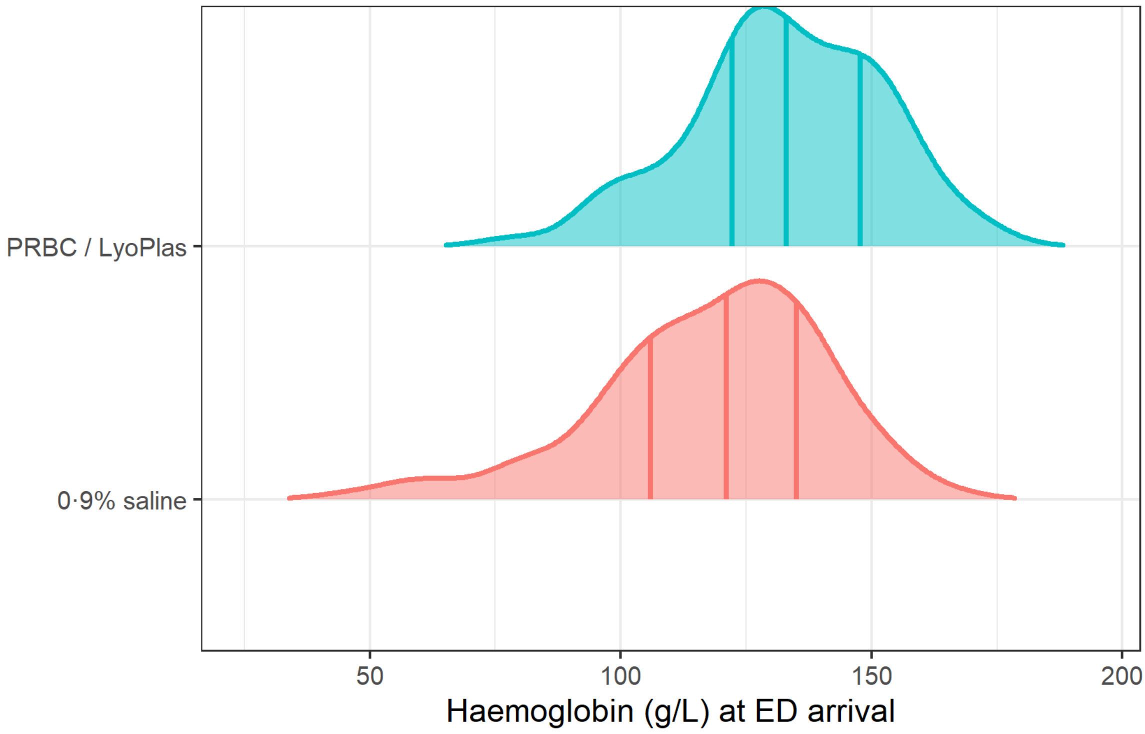

Ridgeplots display the estimated density plots for Haemoglobin concentration on ED arrival by treatment group. Vertical lines within plots denote quartiles. ED = Emergency Department. PRBC = packed red blood cells.

S8 – The RePHILL Collaborative Group

Trial Management Group

Prof Gavin Perkins, Dr Nicholas Crombie, Iain Smith, Dr Heidi Doughty, Dr David Naumann, Hazel Smith, Dr Margaret Grant, Gemma Slinn, Dr Rebekah Wale, Emily Dixon, Dr Karen Piper, Deborah Papoola, Amisha Desai, Natalie Ives, Dr Jon Bishop, Professor Mark Midwinter, Aisling Crombie.

Data Monitoring Committee

Prof John Nicholl (Chair), Dr Jan Jansen, Prof Fiona Lecky

Trial Steering Committee

Prof Ian Roberts (Chair), Prof John Holcomb, Dr Simon Stanworth, Prof Jason Smith, Prof Timothy Coats, Andrew Cox, Timothy Marshall

Blood Banks and Pharmacies

Mike Herbert, Mindy Sahota (New Cross Hospital); Camran Khan, Zeeshan Parvez (Worcester Acute Hospital); Tina Taylor, Julie Nortcote, Mojid Khan (University Hospitals Coventry & Warwickshire); Claire Newsam, Katherine Philpott, Lynne Whitehead (Addenbrokes Hospital); Debbie Asher, Gail Healey (Norfolk and Norwich University Hospital)

Blood Bikers

Midland Freewheelers, Warwickshire & Solihull Blood Bikers

Air Ambulance Sites (Intervention Delivery Sites)

Alistair Wilson (PI), Pam Chrispin (PI), Richard Hinson (East Anglian Air Ambulance); Caroline Leech (PI), Mark Beasley, Sam Cooper (The Air Ambulance Service); Mark Nash (PI) (West Midlands Ambulance Service); Simon Lewis (PI), Oliver Robinson, Rod Mackenzie (MAGPAS Air Ambulance)

Receiving Hospital Sites

David Yeo (PI) (Queen Elizabeth Hospital); Caroline Leech (PI), (University Hospitals Coventry & Warwickshire); Tom James (University Hospitals of North Midlands); Jason Kendall (PI), Johannes Von Vopelius-Feldt (PI) (Southmead Hospital Bristol); Christopher Gough (PI) (Queens Medical Centre Nottingham); Gary Mills (PI) (Northern General Hospital Sheffield); Aquib Hafeez (PI) (Oxford University Hospitals); Sarah Hazleman (PI) (Addenbrookes Hospital); Francoise Sheppard (PI) (Norfolk and Norwich University Hospital); Ben Bloom (PI) (Royal London Hospital)

Birmingham Clinical Trials Unit

Smitaa Patel, Emma Hayes, Emma Homer, Lisa Holden, Anthony Prigg, Adrian Wilcockson
